# Supplementary material for: Mirubactin C rescues the lethal effect of cell wall biosynthesis mutations in Bacillus subtilis
Source: Front Microbiol. 2022 Oct 13;13:1004737. doi: 10.3389/fmicb.2022.1004737 (PMC9609785; doi:10.3389/fmicb.2022.1004737)
Supplement: Supplementary file 1 [file Data_Sheet_1.PDF]

Gene	Lb_Fitness	Lb_200_Fitness	LB_vs_LB_200
aadK	16621.66667	10971.33333	586.4373977
aag	15289	10336.66667	784.3950959
aapA	15087.33333	10019.66667	593.3925297
abfA	16991.33333	12913.33333	2297.476812
abh	16946	11643	1055.466869
abnA	17608.33333	14643	3641.653809
abnB	8215.333333	3855.333333	-1277.448081
abrB	13967	7987	-739.3115331
ackA	11663.66667	8688	1400.766596
acoA	16190.66667	11246.33333	1130.717864
acoB	24083	11962.66667	-3083.926063
acoC	20959	13292.33333	197.555398
acoL	15166	9372.333333	-103.0902158
acoR	23167.5	12758	-1716.606032
acpK	19777.66667	9061	-3295.703711
acsA	13459.66667	8764.666667	355.3271904
acuA	20742.33333	12783.33333	-176.0754581
acuB	18341.33333	13960.33333	2501.022916
acuC	20367.33333	16884.66667	4159.550624
adaA	14457.66667	9702.333333	669.4627545
adaB	14128.33333	9475	647.8905198
adcA	0	1017.333333	1017.333333
adcB	16686.66667	8776.666667	-1648.840012
adcC	9641.666667	5632.666667	-391.2602347
addA	0	0	0
addB	0	0	0
adeC	6427	3508	-507.4653271
adhA	17560	10980.33333	9.184874739
adhB	15446	11114.33333	1463.971198
ahpC	15335.33333	8790.333333	-790.8864082
ahpF	17648	11551	524.870843
ahrC	11133.33333	8401.333333	1445.441942
albA	16443.66667	10918.66667	644.9816892
albB	17396.33333	10905.33333	36.4406434
albC	16611	12597	2218.768391
albE	18084	13352.33333	2053.799807
albG	718	0	-448.5925167
ald	12791.66667	9893.333333	1901.347341
aldX	24564.33333	16398.33333	1051.012844
aldY	27530	15001	-2199.211678
alkA	14396.33333	9933.666667	939.1159685
alrB	15765.33333	9221.333333	-628.5420937
alsD	19267	12393.33333	355.6842814
alsR	12776.66667	8575.333333	592.7190505
alsS	106.6666667	0	-66.64327082
alsT	17630.66667	11463	447.7003745

amhX	14422.33333	9771	760.2050046
ampS	17271	10094	-696.5868467
amtB	17794	11529.33333	411.9861994
amyC	15303.66667	9198.333333	-363.1016872
amyD	20495.33333	10047	-2758.087967
amyE	19269	10885	-1153.898613
amyX	20130.33333	16110.33333	3533.290308
ansA	12472.33333	8939	1146.527299
ansB	6703	6953.666667	2765.761876
ansR	14226	10634.66667	1746.536942
ansZ	15922.33333	8587	-1360.965991
appA	15320.6	8069.333333	-1502.681306
appB	15008	9188.666667	-188.0415376
appC	17063.66667	10548.33333	-112.7156557
appD	14338.66667	9184.666667	226.1449868
appF	17035.66667	10224	-419.5551305
aprE	16603	10306.33333	-66.90002988
aprX	14338	8324.333333	-633.7718261
apt	15597	11099.66667	1354.962651
araA	13735.66667	10058	1476.22106
araB	10975.33333	7975.333333	1118.157286
araD	13776	9840.333333	1233.354907
araE	15209	10497.66667	995.377549
araL	12620.66667	9139	1253.851501
araM	13470.66667	9452	1035.787936
araN	14832.66667	9268.333333	1.170005495
araP	12365	9194.666667	1469.253757
araQ	11974.66667	9147	1665.45981
araR	19901.66667	13782.66667	1348.490153
arfM	25229.66667	13008	-2755.007891
argB	31621	16256.66667	-3499.522707
argC	14792	9146	-95.75558084
argD	13348	9695.666667	1356.094365
argF	0	0	0
argG	13667.33333	10352	1812.914406
argH	13286.33333	9699.333333	1398.289172
argI	13853	9727.666667	1072.580129
argJ	13263.66667	9862	1575.117534
aroA	12209.66667	8489.333333	860.969687
aroB	11973.5	8309.333333	828.5220538
aroC	19219.5	9861.333333	-2146.638637
aroD	16488.33333	7657	-2644.591847
aroH	17313.5	12003.66667	1186.526642
arsB	13715.33333	9696	1126.924934
arsC	15845.66667	11103.66667	1203.600526
arsR	19145.33333	13001	1039.365929
artP	0	0	0

artQ	14136.66667	9992.666667	1160.350681
artR	17225	9998	-763.8469362
aseR	15008.66667	11757.33333	2380.208609
asnH	23263.66667	15903.33333	1368.644228
asnO	0	1102	1102
asnS	18435	12354	836.1684605
aspB	13464.5	9983	1570.640751
atpA	6393.666667	5125.666667	1131.027362
atpC	8944.333333	5191	-397.2465184
atpD	5586.666667	4828.333333	1337.892024
atpE	2881.666667	4653.666667	2853.257053
atpG	0	2660.666667	2660.666667
atpH	7545.333333	5806.666667	1092.488297
atpI	15702	11929.33333	2119.027348
azlB	14401.66667	10253.33333	1255.450472
azlC	17441	13138	2241.20044
azlD	20620	12848	-34.97729022
bacA	14914.66667	10158.66667	840.2713244
bacB	18692.5	13116.66667	1437.954106
bacC	15204.66667	10234	734.4182652
bacD	22294	14354.66667	425.806545
bacE	15460	9849.333333	190.224269
bacF	15681	10059.33333	262.1477423
bcd	11679.66667	9701.666667	2404.436772
bceA	14774	8651	-579.5095289
bceB	22525.66667	15526.33333	1452.732358
bceR	13914.66667	9986.666667	1293.051988
bceS	15400.33333	9082.333333	-539.4971514
bcrC	14692	10103	923.7224856
bcsA	12271	8985	1318.316473
bdbA	14176	9852.666667	995.7759748
bdbB	392.5	0	-245.2264106
bdbD	13603	9732.666667	1233.775295
bdhA	18491	11251.66667	-301.15259
besA	13591	14210	5718.605997
bglA	15193.33333	10350.66667	858.1657794
bglC	14572.33333	10627.66667	1523.154572
bglH	18895	14104	2298.769355
bglP	14920.66667	9470.666667	148.5226404
bglS	13731.33333	9949	1369.928443
bhlA	14676.66667	10994	1824.302456
bhlB	15160.5	6712.333333	-2759.653922
bioB	17719.66667	13562.66667	2491.761562
bioD	10888.33333	7735.333333	932.5132043
bioF	12030.66667	8684	1167.472092
bioI	12817.33333	8915.333333	907.3113035
bioK	13306.33333	13690.33333	5376.793559

bioW	12600.33333	8447.333333	574.8887076
bioY	18330	10314.66667	-1137.562903
bioYB	16642.66667	9253.333333	-1144.682996
bipA	17743.66667	8601.666667	-2484.233174
bkdAA	14392.66667	10330.66667	1338.406831
bkdAB	11493.33333	7645.666667	464.8542359
bkdB	15697.66667	10858.66667	1051.068065
bkdR	16202.66667	12674.33333	2551.220496
blt	20903	11702.66667	-1357.123551
bltD	14226.66667	10335	1446.453755
bltR	12941.33333	9758.666667	1673.171835
blyA	18218.33333	11371.33333	-11.12906235
bmr	19628	13745.33333	1482.138462
bmrA	18737	11342.66667	-363.8486334
bmrR	18500	10822	-736.4422827
bmrU	16147.66667	10959.66667	870.9167658
bofA	14939.5	10854	1520.089271
bofC	13558.33333	10006.33333	1535.348832
bpr	15150.33333	10233.66667	768.0313479
braB	19438.66667	15155.66667	3010.763601
brnQ	15272	8989.333333	-552.3169662
bsaA	24375.5	14970.33333	-259.0077403
bscR	23589.33333	12299.33333	-2438.826008
bsdD	17337.33333	10594.66667	-237.3639641
bsn	14525	11230.33333	2155.39419
buk	15273.66667	10454.66667	911.975066
cah	22074.33333	12292.66667	-1498.949969
carA	0	0	0
carB	0	0	0
catD	17687	9225.333333	-1825.16227
catE	20228	11847.33333	-790.7299366
cccA	18111.33333	11905.33333	589.7224687
cccB	10441.33333	9383.666667	2860.123494
ccdA	27371.66667	15196.66667	-1904.621406
ccpA	12810	7949.333333	-54.1069716
ccpB	14777.33333	10138	905.4078689
ccpC	19286	10582.33333	-1467.186551
ccpN	10164.33333	8506.666667	2156.187738
cdd	18454	11661.66667	131.9642945
cdoA	15615.33333	10603.33333	847.1750059
cgeA	18756	12430	711.6138673
cgeB	12709	9291	1350.662542
cgeC	18281.5	14329.66667	2907.738959
cgeD	18737	13217.66667	1511.151367
cgeE	21478	12829.66667	-589.3724332
cggR	32181.33333	15940	-4166.274806
cheA	0	237.3333333	237.3333333

cheB	21520	12852.33333	-592.9465544
cheC	10858.33333	8811	2026.923291
cheD	11858	6771	-637.6491129
cheR	19830.33333	12095.33333	-294.2754926
cheV	21110.66667	12213	-976.536336
cheW	11886	8711	1284.857029
cheY	19114.33333	10693	-1249.265871
cidA	4050.5	1969	-561.6740792
cimH	18268.33333	10251.33333	-1162.368096
cinA	16173.33333	9046.333333	-1058.452605
citA	18737.66667	12307.33333	600.4015129
citB	18547	11058.66667	-529.1403072
citH	25305	14662.33333	-1147.741367
citM	0	731	731
citR	28952.5	16683.33333	-1405.628839
citS	14970	8959.666667	-393.2998724
citT	21035.33333	13706.33333	563.8638074
citZ	13507.33333	8871.333333	432.2126454
clpC	15273.5	9332	-210.5874705
clpE	23068	11352	-3060.440356
clpP	7772.666667	6995.333333	2139.121493
clpQ	19826.66667	13993	1605.682036
clpX	11129	10226	3272.815991
clpY	20432.33333	12483	-282.7267856
clsA	14170.33333	9567.333333	713.9830652
clsB	11852.66667	9217.333333	1812.016384
coaA	16421	11209	949.4767176
coaX	28826.5	21220.33333	3210.093525
codY	15885	10508.33333	583.6924868
coiA	24066.5	11163.66667	-3872.617182
comA	12975.66667	12823.66667	4716.721032
comC	13106	9384.666667	1196.291285
comEA	19887	12761.66667	336.6536031
comEB	15418.66667	10173.33333	540.0485364
comEC	21303.66667	14329.66667	1019.547663
comER	19377.66667	11646.66667	-460.1247787
comFA	16998.33333	10863	242.7700143
comFB	19296.66667	11006	-1050.184212
comFC	17360.33333	11002.33333	155.9327473
comGA	0	0	0
comGB	14876.66667	10829.66667	1535.01299
comGC	14088	10576.33333	1774.42334
comGD	0	0	0
comGE	16295.33333	11566.66667	1385.657488
comGF	35728	18779.66667	-3542.496894
comGG	12070.33333	10553.66667	3012.355793
comK	18458.5	9835.666667	-1696.847218

comN	13818	10424.66667	1791.447452
comQ	12440	9908.333333	2136.061874
comS	20847	11053	-1971.802501
comZ	14414.5	8376.333333	-629.5675469
copA	22210	11564.66667	-2311.711879
copB	16024	10998	986.5146412
copZ	15115	10007.33333	563.773598
cotA	16550.5	11146	805.5676216
cotB	17282.33333	13897.66667	3099.998972
cotC	27646	14195.66667	-3077.019568
cotD	12425	8861.666667	1098.766917
cotE	14997.33333	9005.666667	-364.3772105
cotF	23872	13738.66667	-1176.097343
cotG	13818	11062	2428.780786
cotH	193	0	-120.5826681
cotI	18351.33333	11864.66667	399.1084427
cotJA	27073.5	15529	-1385.999305
cotJB	0	0	0
cotJC	18913.5	10987.33333	-829.4557539
cotM	15276	9566	21.85057782
cotP	12959.33333	8994	897.2591159
cotR	16634	10253.33333	-139.2682305
cotS	14274.33333	10471.33333	1553.005876
cotSA	14103	10543.66667	1732.384963
cotT	14518.33333	8158.333333	-912.4406058
cotU	17403	9413.666667	-1459.391228
cotV	15287.33333	9245.666667	-305.563603
cotW	19734.33333	11575.66667	-753.9632155
cotX	15306	8628.666667	-934.2261754
cotY	17163.33333	10609.66667	-113.6521286
cotZ	23154.66667	12640	-1826.588013
coxA	17538.66667	12042.33333	1084.513529
cpgA	11205.33333	2719	-4281.8756
crcBA	395.5	0	-247.1007526
crh	20814.66667	14063.33333	1058.732074
csaA	19652	11177.33333	-1100.856274
csbA	13359.66667	9214.333333	867.4719235
csbB	18129.33333	12621.66667	1294.80975
csbC	23285	14420	-128.0177596
csbD	10779.66667	7961.333333	1226.406036
csbX	22193.33333	14343	477.0344652
csfB	27327	15689	-1384.381203
csgA	15666.33333	12820.66667	3032.644525
cshA	6512	3291.666667	-776.9050168
cshB	10900.66667	6595.333333	-215.1924239
csn	16232	11050.66667	909.2269298
csoR	15315.33333	10142.66667	573.9425384

cspB	0	84.33333333	84.33333333
cspC	23299.66667	14710.33333	153.152124
cspD	12957	8810.333333	715.0502708
cspR	0	26.33333333	26.33333333
csrA	16441.33333	11290.33333	1018.106177
cssR	17123.33333	11757.66667	1059.339098
cstA	18778.33333	12559.33333	826.9937659
ctaA	7645.333333	4953	176.343564
ctaB	17750	11112.33333	22.47654859
ctaC	16217.66667	8975.666667	-1156.817881
ctaD	14911.66667	8505.333333	-811.187667
ctaE	16318.66667	9219.333333	-976.2540611
ctaF	16856.66667	8493.666667	-2038.052725
ctaG	15596	8743	-1001.079235
ctaO	22932.66667	11412.33333	-2915.553372
ctc	30216	14771	-4107.372541
ctpA	12767.5	9028.333333	1051.446207
ctpB	26330.66667	18567.33333	2116.441932
ctrA	20169.33333	11218	-1383.409471
ctsR	25727.5	15068	-1006.044531
cueR	15159.66667	8866	-605.4666049
cwlA	16727	10999.66667	548.9605011
cwlC	17479.66667	9261.333333	-1659.624412
cwlD	16338.33333	9058	-1149.874747
cwlH	14149	9096.666667	256.6450527
cwlJ	14914.66667	9386.333333	67.93799105
cwlK	15190.33333	8646.666667	-843.9598786
cwlO	0	728.6666667	728.6666667
cwlS	20525	11581.66667	-1241.95646
cycB	212.5	43.33333333	-89.43255775
cydA	16027.66667	10200.66667	186.8904454
cydC	15781	9057.666667	-801.9969908
cydD	19615.66667	12517.66667	262.1774233
cyeA	16703.66667	9928.666667	-507.4612834
cyeB	877	0	-547.9326423
cymR	16985	9550.333333	-1061.566244
cypA	19978.66667	12898	415.7153756
cypB	17179	11019.33333	286.2263077
cypC	14496.66667	10333.33333	1276.096309
cypX	17989	12429	1189.820637
cysC	12152.33333	9154.333333	1561.790445
cysE	20141.5	11741	-843.0197425
cysH	12183.33333	9657.666667	2045.755578
cysI	124.3333333	0	-77.68106255
cysJ	11175	8650	1668.076081
cysK	23621.5	11155.33333	-3602.92312
cysL	14456.66667	10054.33333	1022.087535

cysP	12629	8965.333333	1074.978329
czcD	0	0	0
czrA	14092.66667	10199.33333	1394.507697
dacA	21227.66667	10733.66667	-2528.969007
dacB	17604.66667	9844	-1155.055328
dacC	13285	8512	211.7888797
dacF	14579.66667	8769	-340.0938199
dat	15694.66667	8706.333333	-1099.390927
dck	147	1205.666667	1113.823909
dctB	17846.33333	12551.33333	1401.289345
dctP	15127	10071.66667	620.6095634
dctR	21185.33333	12239.66667	-996.5199589
dctS	14873.66667	8989.333333	-303.4460017
deaD	19022.33333	12549.33333	664.5472839
defA	14392.66667	10128.33333	1136.073498
defB	17589.33333	7794.333333	-3195.142025
degA	0	0	0
degQ	10821	6277	-483.7515644
degR	19703.5	8889	-3421.365812
degU	24482	13529.66667	-1766.213548
deoC	15272.66667	10702.33333	1160.266513
deoD	14375	9519	537.777956
deoR	13624.66667	9914.333333	1401.905048
des	13715.33333	10036.33333	1467.258267
desK	15694.66667	9015.666667	-790.0575935
desR	14739.66667	10590.33333	1381.274607
dgk	16823	10689	178.3148907
dgkA	16612	10133.33333	-245.5230559
dhaS	13470.66667	9200.333333	784.1212698
dhbA	23049.33333	15586.33333	1185.55555
dhbB	17723.66667	11466.66667	393.2624394
dhbC	17268	12176.66667	1387.954162
dhbE	10038.33333	6397.666667	125.9101019
dinB	13973.66667	9745.666667	1015.189929
dinF	23298.33333	14249.33333	-307.0148351
dinG	0	2237	2237
disA	19765	11536.33333	-812.4564892
divIVA	17095.33333	11378	697.1662899
dltA	14172.5	7934.333333	-920.3706262
dltC	14271.5	8786.666667	-129.8905786
dltD	15823.5	9337	-549.2168357
dltE	19419.33333	11764.66667	-368.1573064
dnaJ	13909.33333	7893.666667	-796.6158481
dnaK	16228.66667	8937	-1202.357135
dppA	15321.66667	8411	-1161.681072
dppB	14642.83333	8227	-921.5591318
dppC	16648.66667	9500.666667	-901.0983469

dppD	0	24.33333333	24.33333333
dppE	15526	9460.666667	-239.6779215
dprA	20863.66667	11348.33333	-1686.882179
dps	17420.33333	11952.33333	1068.445907
drm	1683.666667	0	-1051.922378
dsdA	14320.66667	10176	1228.724372
dtd	0	0	0
dtpT	11581.66667	8084	847.9986106
dusB	25845.66667	12826	-3321.87278
dusC	26843	12084.33333	-4686.654029
eag	0	0	0
ebrA	15188.33333	8832.333333	-657.0436506
ebrB	15009.66667	9185	-192.7495054
ecsA	12269.66667	5896.666667	-1769.18382
ecsB	17878.5	11956.33333	786.1922333
ecsC	14306	8971.333333	33.22115516
efeM	17529	11852.66667	900.8864087
efeU	19005.66667	12934.66667	1060.293628
efp	13758.66667	8464	-132.1488948
eglS	17324	11583	759.2997781
endB	10060.66667	9040.666667	2754.956667
epr	0	761	761
epsA	12491.33333	10007.66667	2203.323133
epsB	12081.5	8544.666667	996.3790754
epsC	12573	9236.333333	1380.966046
epsF	12230	8165.666667	524.5991468
epsG	17798.33333	10024.33333	-1095.721183
epsH	0	449.3333333	449.3333333
epsJ	22396	14426.33333	433.745584
epsK	15042.33333	8594	-804.159007
epsL	13681.33333	8026.333333	-521.4991901
epsM	14658	8325.333333	-832.7016385
epsN	15278.66667	8501.666667	-1044.148837
epsO	12101.66667	9110.666667	1549.779332
estA	0	930.6666667	930.6666667
estB	17958.66667	11016.33333	-203.8943499
etfA	24157.33333	14337.66667	-755.3680921
etfB	18993.33333	13187	1320.33259
exlX	13440.33333	9045.333333	648.0729499
exoA	17121.5	10339.33333	-357.8488041
exuM	0	285.6666667	285.6666667
exuR	24484	11527	-3770.129776
exuT	14852.33333	8951.333333	-328.1173476
ezrA	11839.33333	9687.333333	2290.346793
fabHA	15431.5	10422	780.6971846
fabHB	26695	14473	-2205.519824
fabI	13925	9052	351.9292548

fabL	12896.5	7971.666667	-85.81716569
fadA	28448.33333	16861	-912.9685877
fadB	20813	13832	828.4400416
fadF	37111.33333	16399	-6787.443479
fadG	21901.33333	14363.33333	679.8037524
fadH	22142	13171.33333	-662.5601274
fadM	13039	9401.333333	1254.818256
fadN	12544	9654	1816.751352
fadR	13476	8781.666667	362.1224396
fapR	21854	11725	-1928.95663
fbaA	11476.33333	7560.666667	390.4755072
fbp	20873	18663	5621.953202
fdhD	14932	9900.333333	571.1084595
fer	10563	9240	2640.441847
feuA	14771.66667	4425.666667	-4803.385041
feuB	13184.66667	2490.666667	-5746.858127
feuC	14005.33333	2487.333333	-6262.928125
fhuD	24552.33333	11367.33333	-3972.489788
fhuG	16170.66667	10573.66667	470.5468105
flgB	10935.33333	9989.333333	3157.148513
flgC	0	307	307
flgD	15115	10714	1270.440265
flgE	23100	14897	464.5666632
flgK	19362	13913.66667	1816.663452
flgL	15434.33333	12249.33333	2606.260306
flgM	5987.666667	6141.333333	2400.354978
flhA	11783.66667	2109.666667	-5252.540417
flhB	16095.66667	9116	-940.2613064
flhF	9844	9126.333333	2975.992478
flhO	20870.33333	14529.66667	1490.28595
flhP	23225	14660.33333	149.8024136
fliD	24826.66667	16980	1468.778717
fliE	18685.66667	15010	3335.556774
fliF	21289.66667	12591.33333	-710.0387415
fliG	4613	7337.666667	4455.553464
fliH	23681.66667	15020.33333	224.4859104
fliI	18121.33333	9849.666667	-1472.192005
fliJ	13634	10323.33333	1805.073761
fliK	12152.33333	9555	1962.457112
fliL	15142.33333	11198.33333	1737.69626
fliM	3951.333333	0	-2468.716663
fliP	15679	12721	2925.06397
fliQ	19135.33333	14127.66667	2172.280402
fliR	31337	19955	376.2483345
fliS	22478.33333	17047.66667	3003.638643
fliT	24373.33333	16011.33333	783.3459512
fliW	21319.33333	14583.33333	1263.426099

fliY	15296	12175.33333	2618.688298
fliZ	20788.66667	13501.66667	513.3097045
fmnP	24599	14973	-395.979552
fmt	7229.666667	5519.666667	1002.710727
fni	1866.5	38.33333333	-1127.819776
fnr	19411.33333	13174.33333	1046.507606
folD	9957.333333	6396	174.850669
folEB	16224.66667	12178.33333	2041.475321
fosB	14994.33333	8949.666667	-418.5028685
frlB	16515	10542.33333	224.0806685
frlD	17026.33333	12011	1373.276156
frlM	15324	10440.33333	866.1944393
frlN	19138	13809.66667	1852.61432
frlO	21667.33333	11238	-2299.330906
frlR	16284.33333	11803	1628.863408
fruA	15375.33333	8782.666667	-823.5443015
fruK	14805	8506.333333	-743.5443961
fruR	15856.66667	8531	-1375.938728
ftsE	14465	9084.333333	46.8810296
ftsH	0	0	0
ftsR	16293.66667	11386.33333	1206.365455
ftsX	7224	6296.333333	1782.917817
fumC	9018.333333	9357	3722.519712
fur	9015.333333	472.3333333	-5160.272612
gabD	13326	8871	545.1728725
gabP	18375.33333	11036.66667	-443.8862933
gabR	17844	11917.66667	769.0804995
gabT	18418.33333	10756.33333	-751.0851951
galE	11259	8268	1233.594505
galK	11643	6183.666667	-1090.654603
galT	15783	10148.33333	287.4201145
ganA	20332.66667	13048.33333	344.8763539
ganB	15574.33333	10730	999.4576797
ganP	23209	14331	-169.5344291
ganQ	21816.66667	14427	796.3685153
ganR	23069	14518	104.9348638
gapB	11427	8583.333333	1443.964687
garD	13785.66667	9577.333333	964.3153606
gbsA	19029	13754	1865.048746
gbsB	11731.66667	8785.333333	1455.614844
gcvPA	15677	10124.33333	329.6468649
gcvPB	14730	9418	214.9808203
gcvT	17322.66667	11489	666.132819
gdh	13985.66667	10054.66667	1316.692561
gerAA	12465.66667	8859	1070.692504
gerAB	13080	9119.333333	947.2022491
gerAC	14538	9891.666667	808.6053745

gerBA	15051.33333	9738	334.217967
gerBB	16368.66667	11131.33333	904.5069057
gerBC	19283.5	12137.66667	89.70873379
gerD	12131	8626	1046.785766
gerE	26361.33333	14120.66667	-2349.384675
gerKA	12845.33333	10231.33333	2205.817445
gerKB	12245.33333	10203.33333	2552.685843
gerKC	11965.33333	8926.333333	1450.624429
gerM	16360.33333	9799	-422.6199221
gerPA	0	0	0
gerPB	15957	11150	1180.374946
gerPC	15856.5	9874.333333	-32.50126426
gerPD	0	0	0
gerPE	15150.66667	11516.66667	2050.823088
gerPF	0	0	0
gerT	13330	9487.333333	1159.007083
ggaA	14064.66667	11516.33333	2729.001555
ggaB	12369	10749	3021.087968
ggt	15688.33333	9027.666667	-774.1006493
glcD	19551.33333	12025.33333	-189.9616874
glcF	0	0	0
glcK	13517	8919.666667	474.5064323
glcP	14848	9021.666667	-255.0766314
glcR	0	0	0
glcT	20070	10857	-1682.347925
glcU	14833	9512.333333	244.9617453
glgA	17077	10977.33333	307.9539354
glgB	13838.66667	9715.666667	1069.535319
glgC	12804	9380.666667	1380.975046
glgD	20775.66667	14847.66667	1867.431853
glgP	15519	9864.666667	168.6955432
glnA	5194	2689.333333	-555.7774351
glnH	14568.33333	8788.666667	-313.3463057
glnJ	12393	9834.666667	2091.759899
glnK	16736	10734	277.6708085
glnL	20115.33333	11475.66667	-1092.004648
glnM	12436.33333	9270.333333	1500.352736
glnP	16703.33333	9357	-1078.91969
glnQ	18556.66667	10622	-971.8465203
glnR	16356.33333	9943.666667	-275.4541328
glnT	19385	12432.66667	321.2934964
glpD	19265	11673.33333	-363.0661573
glpF	13455.33333	9669.333333	1262.70124
glpG	12772.33333	9324.333333	1344.426433
glpK	22164.5	14350.33333	502.3823077
glpP	15412.5	11107	1477.568017
glpQ	18069.33333	11007.66667	-281.7034101

glpT	18303	12165.33333	729.9728414
glsA	18215.66667	11753.66667	372.8703528
glsB	19457.66667	10815.33333	-1341.440565
gltA	13162	9325.333333	1101.970235
gltB	21967.66667	14651	926.026635
gltC	13136	10136.66667	1929.547865
gltP	12562.33333	9839	1990.29704
gltR	17451.33333	10684.66667	-218.5889598
gltT	16088.5	10913.33333	861.5496217
glxK	19220.33333	11801.66667	-206.8259543
glyA	9990.666667	8215.666667	1973.691314
gmuA	11523.66667	7534	334.2358891
gmuB	14403.66667	9691	691.867577
gmuC	14424	9748.666667	736.8303702
gmuD	0	0	0
gmuE	15180	10339.33333	855.1628549
gmuF	23969.33333	12820.66667	-2154.909327
gmuG	19581	11692.33333	-541.4968471
gmuR	14460	10261	1226.6716
gndA	18646	15308.33333	3658.673074
gntK	20648.66667	13116.66667	215.7789974
gntP	13657.66667	9895.666667	1362.620619
gntR	25895.33333	16625	446.0964473
gntZ	14038	11388	2617.32904
gpr	19075	14255.66667	2337.975502
gpsA	0	4333	4333
gpsB	12055	8467	935.2690963
greA	13756.33333	9059.333333	464.6422601
grpE	15568	8968	-758.585376
gsaB	13941.5	9882	1171.620374
gsiB	14157	9610.666667	765.6468074
gspA	15410.33333	10471	842.9217086
gtaB	14161.66667	9797.333333	949.397831
guaA	8675.5	7296	1875.71535
guaB	12252.5	9078.333333	1423.208249
guaC	15711	11819	2003.070989
guaD	18367.33333	9517.666667	-1957.888048
gudB	15761	10325.33333	478.1652891
gudD	14778.66667	9861	627.574828
gudP	0	1021.333333	1021.333333
gutB	23465.5	13558.33333	-1102.457336
gutP	20441	11705.33333	-1065.808218
gutR	16647	9689	-711.7237124
hag	22790	15606.66667	1367.915336
helD	13803.33333	10119	1494.944236
hemA	0	0	0
hemAT	21716	11128.33333	-2439.403565

hemB	31	0	-19.36820058
hemC	0	0	0
hemD	0	0	0
hemE	0	0	0
hemH	0	0	0
hemL	504	0	-314.8894546
hemN	14541.66667	9481.666667	396.314512
hemX	0	0	0
hemY	0	0	0
hemZ	15135	9226	-230.0553486
hepS	20026.66667	12042	-470.2740963
hepT	8038.5	6447.666667	1425.3673
hinT	16915.5	7880.333333	-2688.143987
hisA	14984.33333	10376.66667	1014.744938
hisB	21243	9693.333333	-3578.882311
hisC	15599	11177.66667	1431.71309
hisD	17555	11397.33333	429.3087781
hisF	17714	12215.66667	1148.301986
hisG	16016.66667	10407	400.0963661
hisH	711	0	-444.2190521
hisI	20439	12584.66667	-185.2253234
hisJ	13931.33333	9996.333333	1292.305644
hmp	18423.33333	9429	-2081.542432
hom	19980.33333	12668.33333	185.0074078
hprK	7269.666667	4617.666667	75.71950012
hprT	7899.666667	2835.666667	-2099.892318
hrcA	15204.66667	10109	609.4182652
hslO	23045.5	14607.66667	209.2838761
htpG	16000	10096	99.50937714
htpX	16117.33333	8579.333333	-1490.464887
htrA	0	0	0
htrB	9101.333333	4787	-899.3370826
htrC	31164.33333	23031	3560.127129
hutG	15689.66667	10712.66667	910.0663098
hutH	23107.33333	13339.66667	-1097.348395
hutI	25290.66667	14216.66667	-1584.452845
hutM	17986	12153.66667	916.3616452
hutP	15408.66667	10703.33333	1076.296343
hxlA	16321.66667	11800.66667	1603.20493
hxlB	14023.33333	10539	1777.49249
hxlR	13764	10729.33333	2129.852275
icd	8177.333333	8067.666667	2958.626917
ilvA	15230	10384.33333	868.9238217
ilvB	0	565	565
ilvC	25010.33333	13754.33333	-1871.639332
ilvD	13670	9476	935.2483241
ilvE	0	205	205

ilvH	15241	10618.66667	1096.384568
ilvK	16309	11305.33333	1115.785485
immA	20881	12853.33333	-192.7117102
immR	14393.66667	10312.66667	1319.78205
iolB	30061	15744	-3037.531538
iolC	16390	10767	526.8449182
iolD	17094	10590.33333	-89.66733587
iolE	22549.33333	10725.33333	-3363.054118
iolG	21438.33333	13376.66667	-17.58946687
iolH	15718	10830.33333	1010.030858
iolI	13113.33333	8856.333333	663.376227
iolJ	14604.66667	9838	713.2866635
iolR	14872.33333	10597.33333	1305.387039
iolS	8845.333333	6360.666667	834.273434
iolT	16916	10215.66667	-353.1230444
ipi	14511.5	9116.333333	49.82872873
iscS	20595.66667	12407.66667	-460.1076274
iseA	16962.33333	10029.33333	-568.4045485
ispA	12133.83333	7970.833333	389.8488873
kamA	14143	10305.66667	1469.393737
kapB	11970.33333	9028.666667	1549.833859
kapD	14023	10121.33333	1360.034083
katA	18593.5	9846.666667	-1770.192608
katE	15019.66667	9870	486.002688
katX	16028	10394.66667	380.6821852
kbaA	14395.66667	11775.66667	2781.532489
kbl	39074.66667	18928	-5485.096183
kdgA	12408.5	9248.666667	1496.075798
kdgK	12753	9343.666667	1375.83886
kdgR	0	0	0
kdgT	17287.66667	10079	-721.9998578
kduD	14810.5	10011.33333	758.0193102
kduI	13829	9382.666667	742.5748652
khtS	20336.33333	10662	-2043.747842
khtT	16974.33333	9598.666667	-1006.568583
kinB	16139.66667	9299.333333	-784.4183223
kinC	13533.66667	8469.333333	13.76008794
kinD	19669	10305	-1983.810879
kinE	15681.33333	10394.66667	597.2728154
kipA	14050.33333	9544.666667	766.2900782
kipI	13949	9445.333333	730.2678522
kipR	15902.66667	11884.66667	1948.988028
ksgA	15820	10330.66667	446.6365633
ktrA	13996.66667	10073	1328.153307
ktrB	14056.66667	9505.666667	723.333134
ktrC	14304.66667	8359.333333	-577.945804
ktrD	8203.666667	4895.666667	-229.82564

lcfA	23773	14903.66667	50.75594308
lcfB	12318.5	7783.333333	86.97272472
lctP	14523.66667	10102.66667	1028.560564
ldh	14094.33333	10152	1346.133062
lepA	17462.33333	11487	576.8717862
leuA	0	0	0
leuB	0	67.66666667	67.66666667
leuC	3152.333333	725.3333333	-1244.18358
leuD	14532.33333	9824	744.4791316
levB	21261.33333	13364.66667	80.99671065
levD	14149.33333	9840	999.7701258
levE	14723.33333	10692	1493.146025
levF	14532.66667	10734.33333	1654.604205
levG	25250.66667	14279	-1497.128285
levR	14773	10032.33333	802.4485851
lexA	13387.33333	6061	-2303.147008
lgt	12495	7542.333333	-264.3010625
liaF	11975.66667	7702.666667	220.5016957
liaG	0	0	0
liaH	25514	15186.33333	-754.3205262
liaI	12601.66667	10074.33333	2201.055667
liaR	17709	12627.33333	1563.092556
liaS	13444.33333	6915	-1484.759506
licA	15653.66667	10000	219.8917471
licB	18087.66667	12961.66667	1660.842278
licH	11498.33333	9160.666667	1976.730333
licR	13480	10240	1817.95665
licT	14938.33333	9778	444.818182
ligB	27827.5	14762.66667	-2623.417259
lipA	16166	10678.33333	578.1291203
lipC	17716	8370.333333	-2698.280909
lmrA	13264.33333	8209	-78.29898658
lmrB	16323	9041.666667	-1156.628111
lonA	10054	7515.666667	1234.121872
lonB	31501.33333	18470.66667	-1210.757288
lpdV	20680.33333	13011	90.32760973
lplA	14427.33333	9490	476.0811013
lplB	15485	6692.666667	-2982.061914
lplC	14283	9327	403.2577771
lplD	15767	10414.66667	563.7499385
lrgA	27825	18199.66667	815.1446928
lrgB	14041.33333	9403	630.2464376
lrpA	13691.66667	10597	2042.71141
lrpB	20401.33333	11436	-1310.358585
lrpC	12619.66667	8375.333333	490.8096148
lspA	11327	9396.333333	2319.442753
ltaSA	17779.66667	11529	420.6080556

luxS	13693	9616	1060.878369
lysA	21499	14524.33333	1092.17384
lysC	18515.33333	14144	2575.977747
lysP	13322.66667	9507	1183.255475
lytA	9187.666667	9575.333333	3835.056853
lytB	15734.5	11451.33333	1620.721977
lytC	11603.66667	8986.333333	1736.586769
lytD	14643	8691	-457.6632619
lytE	0	957	957
lytF	0	0	0
lytG	16361	9810	-412.0364425
lytH	18046	10343	-931.7918613
lytR	13718	9792.333333	1221.592186
lytS	14482	11422.33333	2374.259758
lytT	14968.66667	9635	282.8665019
maeA	22912	14572.66667	257.6920947
maeN	14701	11169	1984.09946
maf	0	0	0
malA	15808	11209.33333	1332.800598
malK	17425.33333	13595.33333	2708.322004
malL	12237.66667	10750	3104.142495
malP	16022	10855	844.7642025
malQ	0	53.33333333	53.33333333
malR	19524	11214.33333	-983.8843492
malS	18530.66667	12498.66667	921.0644436
manA	10188.66667	5559.333333	-806.3485912
manP	16711.33333	8790	-1650.917935
manR	12930.66667	7669.333333	-409.4971717
mapA	22742.66667	15498.33333	1289.154954
mapB	17608	10258.33333	-742.8045971
mccA	27567.33333	14349.66667	-2873.870156
mccB	17123	11076	377.8806915
mcpA	14871.33333	10429	1137.678486
mcpB	13866.66667	10068.33333	1404.708127
mcpC	25597	14280.33333	-1712.177321
mcsA	19991.33333	13350.33333	860.1348205
mcsB	20171.5	9287.666667	-3315.096496
mdh	11202.33333	8881.333333	1882.332076
mdr	12360	6371.666667	-1350.622339
mdxD	12117	9324.333333	1753.866029
mdxE	12192	10434.33333	2817.007479
mdxF	11267.33333	9590.666667	2551.054666
mdxG	14648	10669.66667	1517.879501
mdxR	10556.33333	9052.666667	2457.273718
mecA	27407	13276	-3847.363656
mecB	0	230.6666667	230.6666667
med	0	0	0

melA	17409.33333	11475.33333	598.3184948
menF	19631.33333	13569.66667	1304.389193
menH	12062.5	8616.666667	1080.249908
metA	12276.5	7783.666667	113.5468459
metC	14144.66667	8065.333333	-771.9808977
metE	14495.33333	8097.333333	-959.0706505
metI	20047	10179.33333	-2345.644636
metN	17703.66667	7955.333333	-3105.575281
metQ	11670.33333	6304.333333	-987.0652749
mfd	18772	12701.33333	972.9507101
mgsA	18619.5	11557	-76.10357203
mgsR	13058.33333	9689	1530.40583
mhqA	18745	9714.333333	-1997.180212
mhqR	15636.66667	8111	-1658.486982
miaA	18433.33333	9081	-2435.790238
minC	28836.33333	17296.33333	-720.0501519
minD	23223.66667	13438.33333	-1071.364546
minJ	20599	15278	2408.143104
mleA	13116	10841.33333	2646.710145
mleN	22634	15903.66667	1762.381119
mlpA	20724.66667	11440.33333	-1508.037666
mmgA	1099.666667	256.3333333	-430.7171368
mmgB	16901	10602.66667	43.2486656
mmgC	17105.33333	10794.33333	107.2518166
mmr	21144.5	12981.33333	-229.3414151
mmsA	20411	15130.33333	2377.935202
mntA	0	1046.666667	1046.666667
mntB	14058.66667	10663.66667	1880.083573
mntC	17622	9992.666667	-1017.218193
mntD	20555.33333	17081.66667	4239.091859
mntH	72	0	-44.9842078
mntR	16822.33333	8907	-1603.268589
moaA	18191.66667	10567.66667	-798.1349113
moaB	15855	10950.66667	1044.76924
moaC	0	0	0
moaD	14845.66667	8645.666667	-629.6188098
moaE	15163.33333	8904.666667	-569.0908007
mobA	14814.33333	8581.333333	-674.3756823
mobB	16521	9011.333333	-1310.668015
moeA	14976.33333	8467	-889.9234832
moeB	11761	7606.333333	258.2879449
motA	14756.33333	8315.666667	-903.8050705
motB	22121.33333	13883.33333	62.35200634
mpr	16239.66667	10959.66667	813.4369447
mprF	14186.33333	9880	1016.653241
mraW	11444	7838	688.010082
mraZ	12251.66667	7853	198.3955658

mreBH	19344.33333	12225.33333	139.3679101
mrgA	13977.33333	9957.333333	1224.565733
mrnC	17637	11823	803.7434303
mscL	14989.33333	13011.66667	3646.621035
msmE	17072.66667	9813.333333	-853.3386817
msmR	10505.66667	9201	2637.262605
msmX	22107.33333	10927	-2885.234398
msrA	18306.33333	11204.33333	-233.1097608
msrB	13544.5	9518.333333	1055.991631
mstX	25111.66667	14596.33333	-1092.950439
mta	18738.66667	11939	231.4433989
mtbP	29886.33333	17060	-1612.403182
mtlA	13931	9909.666667	1205.847237
mtlD	13144	10274	2061.882953
mtlF	12389	9645.666667	1905.259021
mtlR	13331.66667	9048	718.6324487
mtnA	19272.33333	9632	-2408.981215
mtnB	18836.66667	9774	-1994.785106
mtnD	15875.33333	10449	530.3986999
mtnE	15161.33333	8885	-587.507906
mtnK	22058.33333	12433.33333	-1348.286812
mtnN	10469.33333	7842.333333	1301.296302
mtnU	21298	10971.33333	-2335.245247
mtnW	0	1041.333333	1041.333333
mtnX	19153.33333	9828.333333	-2138.298983
mtrB	19190.66667	7851	-4138.957461
murAB	17382	8595	-2264.9375
murQ	0	576.3333333	576.3333333
mutL	25242.33333	13871.33333	-1899.588446
mutM	13999.66667	9464	717.2789652
mutS	22983	10958.66667	-3400.667332
mutSB	19876.66667	13868.33333	1449.776337
mutT	19579	11900	-332.5806191
nadA	14695.66667	10159.33333	977.7649565
nadB	16260.33333	11211.33333	1052.191478
nadC	17359	12617.66667	1772.099122
nadR	32206.66667	20757.66667	635.5640837
nagA	18063	11409	123.5868674
nagBA	16195	10572	453.6771477
nagBB	12805.66667	9055.333333	1054.600411
nagP	17517	10691.66667	-252.6162234
nap	0	35.33333333	35.33333333
narG	13777	8972	364.396793
narH	14409	9959	956.5354134
narI	14082.33333	9596.333333	797.9637637
narJ	14800.66667	10011.33333	764.1629867
narK	0	0	0

nasA	0	1064	1064
nasB	0	130	130
nasC	16371	10125.33333	-102.9509158
nasD	14182	9125.333333	264.6939575
nasE	14426.66667	9627	613.4976217
nasF	19440.33333	11143.33333	-1002.611034
natA	15992.16667	9136.333333	-855.2631743
natB	533.6	497.3333333	163.9503711
natK	15931.33333	9555.666667	-397.9223506
natR	14139.66667	9994.333333	1160.143006
ndh	7582.666667	3153	-1584.503514
ndhF	172	960	852.5377258
ndk	11651	9799.333333	2520.013818
ndoA	13869	8066.666667	-598.4163614
nfo	20717.66667	13112.66667	168.6691316
nfrA	0	207.6666667	207.6666667
nhaC	19664.5	10133.66667	-2152.332699
nhaK	15469.66667	10470.66667	805.5180559
nhaX	20102.5	11569.66667	-989.98663
nifS	22911	13696.33333	-618.0164579
nin	13508.66667	10028	1588.046271
norM	14522.5	9685	611.6228081
nosA	20266	13793.66667	1131.861731
nprB	0	0	0
nprE	21109.66667	11102.66667	-2086.244889
nrdEB	15702	14725	4914.694015
nrdIB	17264	8437	-2349.213382
nrdR	20288.33333	12594.66667	-81.09170335
nrnA	16917	9820	-749.4144917
nsrR	17366.5	10311.33333	-538.9200668
ntdA	22338.66667	16097.66667	2140.899675
ntdB	14779	8994.333333	-239.3000989
ntdC	13370.33333	9752.333333	1398.807596
nth	16725.5	10773.66667	323.8976721
nucA	15522.66667	9004.333333	-693.9286526
nucB	18811	13081.33333	1328.584264
nudF	13155.66667	9487	1267.593846
nupC	22875.33333	12386.66667	-1905.399281
nusB	0	0	0
nusG	10655	6368.333333	-288.7046408
oatA	17903	9604	-1581.448226
odhA	3145.5	6988.333333	5023.085755
odhB	0	11339.66667	11339.66667
ogt	16095.66667	8738.333333	-1317.927973
ohrA	21323.33333	12881	-441.4063572
ohrB	17102.33333	9527.666667	-1157.540508
ohrR	62	0	-38.73640116

oppB	15277	8444	-1100.774203
oppC	13578.33333	9053	569.5198849
oppD	17083	9229.666667	-1443.461415
oppF	14376.33333	8771	-211.0550849
opuAA	19114.33333	9076.666667	-2865.599204
opuAB	13577.33333	7910.333333	-572.5220011
opuAC	14748	9070	-144.2652316
opuBA	14162	9886.333333	1038.189571
opuBC	453	483	199.9743592
opuBD	0	245.6666667	245.6666667
opuCA	14728.5	8959	-243.0820087
opuCB	17871.33333	8959	-2206.663505
opuCC	19223.5	10606.66667	-1403.804426
opuCD	21712.5	13469.66667	-95.88349889
opuD	20834	13962.66667	945.9863144
opuE	16610.66667	10543.66667	165.6433184
oxaAB	15850.66667	9313.333333	-589.8567104
oxdC	13885.66667	10482.66667	1807.170628
pabA	18601	11355.33333	-266.2117964
pabB	0	0	0
pabC	13641.5	8901.666667	378.7212397
padC	14782.66667	9707	471.0757054
padR	22682.66667	13324	-847.6915397
paiA	12014.66667	9318.333333	1811.801916
paiB	14884.66667	10395.33333	1095.681411
panB	6692.5	5934	1752.655407
panC	5723.5	4641.666667	1065.734537
panD	10232.5	9211.333333	2818.26519
panE	21682	13159	-387.4943553
patB	12164.33333	7764.666667	164.6264104
pbpA	12687.33333	8531.333333	604.5327898
pbpC	18041.33333	9650.666667	-1621.209551
pbpD	16279.33333	8298	-1873.012688
pbpE	18803.5	12066.66667	318.6034525
pbpF	19044.5	12532.33333	633.6979791
pbpG	13512	9210	767.963669
pbpH	20999.33333	12074	-1045.977422
pbpI	13936.66667	9329	621.640147
pbpX	18004	10201.66667	-1046.884407
pbuE	16811.66667	10467.33333	-36.27092842
pbuG	13710	9069.333333	503.5904309
pbuO	21341	10630.66667	-2702.777482
pbuX	15331	9761.666667	183.154308
pckA	17294	10548	-256.956802
pcp	20271.33333	13420	754.8629013
pcrB	19135.5	12828.33333	872.8429387
pdaA	20339.5	12501.33333	-206.3929807

pdhA	6636.666667	4152	5.538993724
pdhB	5100	1642	-1544.381386
pdhC	10603	7961.666667	1337.117287
pdhD	1463.666667	1049	134.5293682
pdp	14361	9068	95.52488531
pdxK	21735	15321.66667	1742.058936
pdxS	10052.5	8369	2088.392376
pdxT	0	1202.666667	1202.666667
pel	17815	10695.66667	-434.8008612
pelB	13573.66667	8256.333333	-224.2311386
penP	14395	10037.33333	1043.615676
pepA	18009.33333	12217.33333	965.4500964
pepF	19438.5	12450	305.2010642
pepT	15621.33333	10073.66667	313.7596552
perR	0	40.66666667	40.66666667
pfkA	8961.666667	7596	1996.92395
pgcA	11965	6931.333333	-544.1673106
pgcM	12048.66667	9545	2017.226041
pgdS	15998	10768.33333	773.0922718
pgi	13534	11585.66667	3129.885161
pgsA	0	2107.333333	2107.333333
pgsB	13530.5	9810	1356.405227
pgsC	19224.5	12657.33333	646.2374596
pgsE	13082.33333	9264.333333	1090.744428
pheA	15869	10620	705.3556441
phoA	18599.5	11180.33333	-440.2746254
phoB	14508.33333	10407	1342.473867
phoD	14058.33333	8212.333333	-571.0415004
phoE	14371.5	10483	1503.964688
phoH	14588.33333	9542.666667	428.158081
phoP	13063	9948	1786.490187
phoR	12020	9522.333333	2012.469753
phrA	15741.33333	10790	955.1193089
phrC	8998	5705	83.22358597
phrE	17568.33333	13740.66667	2764.311703
phrF	14456	11915.66667	2883.837389
phrG	12803	11816.66667	3817.599826
phrH	17936.5	9819.333333	-1387.045045
phrI	10248.66667	8077.333333	1674.164569
phrK	22056.33333	17208	3427.629416
phy	20048	12379	-146.6027504
pit	13635	8748.333333	229.4489807
pksA	23154.66667	11612.66667	-2853.921346
pksB	14629.66667	9439.333333	299.0004803
pksC	24044	12823.66667	-2198.559617
pksD	15692.66667	8501	-1303.474699
pksE	18580.33333	9857.666667	-1750.966329

pksF	17151.66667	8775	-1941.029687
pksG	17815	9189.333333	-1941.134195
pksH	11419.33333	7475	340.4213384
pksI	24865	12395.33333	-3139.837875
pksJ	24430	12301.66667	-2961.724953
pksL	13525	10065.66667	1615.508187
pksM	22068	12096.33333	-1691.326358
pksN	15225	8179.333333	-1332.952275
pksR	16483	8629.333333	-1668.92635
pksS	22191	11372.66667	-2491.841047
pnbA	15949	9905.666667	-58.96014234
pncA	16150.66667	10183	92.3757571
pnpA	8004.333333	6932	1931.047306
polA	18753	14120	2403.488209
polX	19308.33333	13471.66667	1408.193347
polYA	15170.66667	10676.66667	1198.327474
polYB	16643	10503	104.7754102
ponA	15560.33333	9894.333333	172.5379424
ppaX	13361	9402.666667	1054.972216
ppiB	19944.66667	11148.66667	-1312.375415
ppnKB	16784.66667	12191.66667	1704.931483
pps	18677.66667	10691	-978.4449806
ppsA	12607.66667	10144	2266.973649
ppsB	12017.66667	9922.666667	2414.260908
ppsC	12665.33333	9914	2000.944631
ppsD	14491.33333	10467.33333	1413.428472
ppsE	13808.33333	9863	1235.820332
prkA	12127	7694	117.2848885
prkC	13768	9399.666667	797.6864857
prmA	16901.33333	10878.66667	319.0404054
prmC	14897	10778	1470.642449
proA	14008.66667	7854.666667	-897.6773941
proB	18883.33333	10247	-1550.941537
proG	17832.66667	10661.66667	-479.838653
proH	14154.33333	11132.66667	2289.312889
proI	0	0	0
proJ	18846.66667	13928.66667	2153.633754
prpB	16118.33333	11247	1176.576999
prpC	16213.16667	10508.66667	378.9936323
prpD	21106.33333	13568.33333	381.5043802
prpE	17040.66667	9885	-761.6790338
prsW	15394.33333	9498.333333	-119.7484674
psd	16440.33333	11205	933.3976248
psiE	21159.33333	13908.66667	688.7243384
pspA	18592.5	10566.66667	-1049.567827
pssA	10863.33333	7948.666667	1161.466054
pstA	16189.33333	10263	148.2175714

pstBA	15988.33333	9525	-464.2015151
pstBB	13712.66667	8305	-262.4089842
pstC	14101.66667	10586	1775.551337
pstS	15588.66667	9406.666667	-332.8308431
pta	16611	10543.33333	165.1017248
ptb	15686.33333	10631.33333	830.8155787
ptkA	0	0	0
ptsG	0	0	0
ptsH	14328.5	6577.833333	-2374.33641
ptsI	16916	8787.333333	-1781.456378
pucA	16958.33333	10483.33333	-111.9054258
pucC	16078	10214	168.7764853
pucE	18834.33333	12276.33333	509.0060487
pucF	28404.33333	15731.33333	-2015.144905
pucG	16644.66667	10348.66667	-50.59922421
pucH	18939	11905	72.27900585
pucI	13861.66667	9359.666667	699.1653635
pucJ	27839.66667	15059	-2334.685424
pucK	12391.33333	9408.333333	1666.467866
pucL	13560	9478.333333	1006.30753
pucM	14142.33333	9859	1023.14359
pucR	17542	11589	629.0975934
pupG	12653.33333	8806.666667	901.1086658
purA	15949.33333	10638	673.1649308
purB	12656	11551	3643.775917
purC	18365.5	8115.333333	-3359.07595
purD	16775	11204	723.3043626
purE	12583.5	7660	-201.9274846
purF	17703.5	10145.66667	-915.1378172
purH	18451.5	12358.66667	830.5262462
purK	15512	7730.666667	-1960.930992
purL	18862	14557	2772.387117
purM	18164.5	10938	-410.8283699
purN	18030.5	12188.66667	923.5589057
purQ	14432.5	8571.333333	-445.8135988
purR	18583.66667	10058.66667	-1552.048932
purS	16252.5	9034.333333	-1119.914407
purT	19652.33333	10917.33333	-1361.064534
purU	27989.66667	14869.33333	-2618.06919
pycA	22112	13228.33333	-586.8167075
pyk	6432.333333	6215	2196.202509
pyrAA	16320.66667	11876	1679.163044
pyrAB	12885.66667	9727	1676.284625
pyrB	12149.66667	9403.333333	1812.456527
pyrC	14545.66667	10374	1286.148723
pyrD	25631.66667	16674.33333	660.1636157
pyrE	9681.666667	8525	2476.081872

pyrF	9961.666667	9067	2843.143286
pyrK	14236	10466	1571.622468
pyrP	17771.33333	13367.33333	2264.147894
pyrR	21011.33333	11274.66667	-1852.808123
qcrA	17339	11737	903.9280681
qcrB	19265.5	12010	-26.71188093
qcrC	15877	11346.66667	1427.024065
qodI	20542.33333	11803	-1031.452659
qoxA	0	0	0
qoxB	12621	7395.333333	-490.0234261
qoxD	5874.333333	3017.666667	-652.5032135
queA	15595	11207.66667	1464.212213
queC	14563	7414.666667	-1684.014142
queD	14672.33333	10338.66667	1171.676505
queE	15128	7780.333333	-1671.348551
queF	16910.33333	8771	-1794.249287
racA	30163	16958.33333	-1886.925833
racX	14053	9942	1161.95733
radA	22031.5	14756.33333	991.478136
radC	15195	16336	6842.457812
rapA	18134.33333	10228.66667	-1101.314153
rapB	15880	10693.33333	771.8163901
rapC	17212	10066	-687.7247875
rapD	19309	12264	200.1101602
rapE	16960.66667	11115	518.3034193
rapF	27726	14501	-2821.668688
rapH	17998.5	11017.66667	-227.4481131
rapI	7980	8052	3066.250302
rapJ	14513	10311.33333	1243.891558
rapK	14574	10679.66667	1574.113271
rbfA	9237.666667	6508	736.4844868
rbfK	11506.66667	9734	2544.85716
rbsA	17777	10616.66667	-490.059196
rbsB	14477.66667	9864.666667	819.3004745
rbsD	17104	10569.33333	-116.9151425
rbsK	14548.33333	9330.666667	241.1493076
recA	17627.33333	9790.333333	-1222.88369
recF	13062	8172	11.11496776
recG	34060.33333	20908.33333	-371.9043403
recJ	13771.66667	9616	1011.728957
recN	16817	10855.33333	348.396908
recO	10589.66667	7800.666667	1184.447696
recQ	13919	9409	712.6779388
recR	10539	7809.333333	1224.769916
recU	12947.5	7425.666667	-663.6809796
recX	18058.5	12072.33333	789.7317138
relA	14597.66667	10081	960.6601282

resA	19301	9142	-2916.891594
resB	21378.66667	17689.66667	4332.689113
resC	15434.5	14134	4490.822843
resD	7980.5	7584.333333	2598.271245
resE	11777.33333	6103.666667	-1254.583473
rex	12262.66667	7495.333333	-166.1436882
rghRA	15319	15452.33333	5881.318343
rghRB	13384.66667	9621	1258.519074
rhgT	17565	10519.33333	-454.9390286
rho	24219.66667	15305.33333	173.3539132
ribAB	20372.66667	14142.33333	1413.885127
ribD	12070	9295.333333	1754.23072
ribE	18835	12734.66667	966.9228616
ribH	20768.66667	13205.33333	229.4719844
rimI	20315	14483.66667	1791.247479
rimM	3511	1739.666667	-453.9382444
rimO	19975.33333	12319	-161.2020222
ripX	9722	8234	2159.882385
rlmB	17115.33333	11269.33333	576.00401
rluB	19328	10023.66667	-2052.094006
rluD	17758.66667	10805.33333	-289.9382172
rnhB	44585.33333	23508.33333	-4347.720828
rnhC	14964	12773.33333	3424.115478
rnjB	0	494	494
rnmV	13348.33333	8087.666667	-252.1138957
rnr	15412.66667	9071	-558.5361129
rocA	16134	13387.33333	3307.122102
rocB	17641.33333	12348	1326.036047
rocC	14876.66667	10397.66667	1103.01299
rocD	10329.66667	8632	2178.224002
rocE	15357	11063	1468.243344
rocG	9543	8345.333333	2383.051457
rocR	21419.66667	15050	1667.406439
rok	17126	9170	-1529.99365
rpe	10414.66667	6589	82.1176454
rph	27155	17179	213.081071
rplA	6982	4635.666667	273.4480711
rplGA	19200.66667	8880.666667	-3115.538601
rplGB	14684.66667	9593.666667	418.9708771
rplK	4738.666667	4153	1192.372694
rplO	11343.33333	5365	-1722.095331
rpmB	18158.33333	12176.33333	831.3577775
rpmC	9482	7144	1219.829745
rpmEA	19400.66667	10040.33333	-2080.828067
rpmEB	17789.33333	12685	1570.568509
rpmF	17834.33333	9331.666667	-1810.879954
rpmGA	14747.66667	10295.66667	1081.609695

rpmGB	20342.66667	13045.66667	335.9618806
rpmI	9146	8634.333333	2920.089381
rpmJ	10505	7333.666667	770.3457921
rpoE	20706	12735	-201.7084273
rpoZ	10660.66667	9265.333333	2604.754935
rpsNB	0	0	0
rpsT	18268	12533	1119.506831
rpsU	14854.33333	7388	-1892.700242
rsbP	19736	13121.66667	790.9954834
rsbQ	29654.33333	15921	-2606.454068
rsbRA	14817	10284.66667	1027.291569
rsbRB	21903	14415.33333	730.7624513
rsbRC	12303	8928.333333	1241.656825
rsbRD	14052.33333	10238.33333	1458.707184
rsbS	13703.33333	10464.33333	1902.755635
rsbT	14543	10308.66667	1222.481471
rsbU	15503.66667	10847	1160.608847
rsbV	16858.66667	12300	1767.031047
rsbW	12876.33333	9228.666667	1183.782578
rsbX	12268.66667	9516	1850.774294
rseP	0	0	0
rsfA	16455	11743.66667	1462.900842
rsgI	14683	10632.33333	1458.678845
rsiV	20510	10939	-1875.251417
rsiW	14195.33333	7775	-1093.969785
rsiX	17854.5	5626.666667	-5528.479697
rsmB	14167.33333	9706.333333	854.8574072
rsmE	23646.66667	15672.33333	898.3532336
rsoA	24062.33333	13972.66667	-1061.013929
rtp	19932	11397.66667	-1055.461527
rtpA	14315.66667	10136.33333	1192.181609
ruvA	12477.66667	8079	283.1951357
ruvB	12116.66667	7543.333333	-26.92571127
sacB	18800.66667	13947	2200.706998
sacC	15370.66667	10189.33333	586.0380083
sacP	21209.33333	14208.66667	957.4853052
sacT	16170.33333	10696.33333	593.4217374
sacV	11617.33333	9947	2688.714767
sacX	14998.33333	10764	1393.331342
sacY	17105.33333	11501	813.9184833
safA	26635	15819.66667	-821.3663171
salA	8642	6702.333333	1302.978836
sapB	16313	10499.33333	307.2863627
sat	19222.33333	13510.66667	1500.924484
sbcC	0	0	0
sbcD	26511.5	16135.33333	-428.5392384
sboA	15717	10987.66667	1167.988972

sboX	15090	10352	924.0597813
sbp	13232.66667	7279	-988.5142656
scoA	19835.33333	13730.66667	1337.933937
scoC	24165.5	13531.33333	-1566.803801
scuA	26824.33333	15672.66667	-1086.658123
sda	17963.33333	12111	887.8566736
sdaAA	13763	9553.333333	954.4770557
sdaAB	13653	9094.666667	564.536262
sdhA	8750	0	-5466.830809
sdhB	0	469.6666667	469.6666667
sdhC	0	373	373
sdpI	11201.33333	6794.666667	-203.7098102
seaA	12621.5	9386.666667	1500.997517
secDF	11339.33333	6480.666667	-603.9295418
secG	24957.5	9598.666667	-5994.296753
senS	22514.5	12337.33333	-1729.290925
sepF	18216.33333	12423	1041.787166
serA	22833	13832	-433.6168995
serC	16459	11151.66667	868.4017191
sfp	21024.66667	12575.5	-560.3051989
sigB	17009.33333	11404.66667	777.5640937
sigD	24375.33333	16192	962.7630565
sigE	16142	8796.666667	-1288.54281
sigF	12197	8815.333333	1194.883575
sigG	17516	10426.66667	-516.9914427
sigH	13738.33333	9429.666667	846.2216454
sigI	19333.66667	9764	-2315.301096
sigL	17069	10037.66667	-626.7144859
sigM	0	0	0
sigO	12667	9951	2036.90333
sigV	16578.33333	10603.33333	245.5112265
sigW	13600.33333	7602.666667	-894.558623
sigX	18880	10551.66667	-1244.192268
sigY	15408.33333	12403.33333	2776.504603
sigZ	17151.66667	11010.33333	294.3036458
sinI	11849	9248.666667	1845.64058
sipS	12784.33333	8537.666667	550.2623988
sipT	16023.33333	9089.666667	-921.4021717
sipU	14791.66667	9731	489.4526794
sipV	22554	12031.66667	-2059.636428
sipW	14931	7918.666667	-1409.933426
sirB	10906	9015	2201.142079
sirC	16188.66667	11502	1387.634092
skfA	18577	10728	-878.5503938
skfB	15870.66667	10058.33333	142.6476763
skfC	15942.66667	10329.66667	368.9968019
skfE	14876.33333	9137.666667	-156.7787502

skfF	0	1041.333333	1041.333333
skfG	18191.33333	12546	1180.406682
skfH	17188	9277	-1461.730052
sleB	16040	11568.66667	1547.184817
slp	14001	8870.666667	123.112591
slrA	23011.33333	17635.66667	3258.630549
slrR	16117.33333	11017	947.2017792
smpB	10051	6466	186.3295468
sodA	20334.33333	13640.33333	935.8350528
sodC	12664.5	8836	923.4652817
sodF	12906.66667	7923.666667	-140.1691024
spbC	11421.33333	7125.333333	-10.49488962
speA	17923.66667	7865.666667	-3332.693693
speB	12304	8596.333333	909.0320444
speD	12828.33333	10105	2090.105383
speE	16256.33333	10888	731.3572669
splA	23415.33333	11363.33333	-3266.114173
splB	0	0	0
spmA	25046.66667	16553	904.3269708
spmB	17024	11085.66667	449.4006439
spo0B	21636.33333	12027.66667	-1490.296038
spo0E	20761	10729.66667	-2241.404697
spo0F	23471.5	14319	-345.5393534
spo0M	97.66666667	917.6666667	856.6464218
spoIIAA	20358	9672.333333	-3046.951423
spoIIAB	17858	10977.33333	-179.9997631
spoIIB	15729.66667	9970.333333	142.74175
spoIID	23634.33333	14018.33333	-747.9411382
spoIIE	15789.5	9127	-737.9742931
spoIIGA	8190	6539.666667	1422.713029
spoIIIAA	18946.66667	11690	-147.5109792
spoIIIAB	15974	9967.333333	-12.91299227
spoIIIAC	16876.33333	10131	-413.0067447
spoIIIAD	16020	9772.333333	-236.6529028
spoIIIAE	15622	9263.333333	-496.9901986
spoIIIAF	15294.66667	8702	-853.8119946
spoIIIAG	16426	10078.33333	-184.3138524
spoIIIAH	16878.66667	9573.333333	-972.1312329
spoIIIC	14074	10906	2112.836936
spoIIID	17144.5	9873.666667	-837.8854261
spoIIIE	18167.33333	9433.666667	-1916.931915
spoIIM	20132	9764.333333	-2813.750993
spoIIP	20258.33333	11445.66667	-1211.348283
spoIIQ	21356.5	11927.33333	-1415.794916
spoIIR	13702.5	7827.666667	-733.3903808
spoIISA	15678.66667	11308.33333	1512.605564
spoIISB	12973	8507.666667	402.3871135

spoIVA	15041	9204.666667	-192.6592995
spoIVB	22675.66667	12431	-1736.318075
spoIVCA	15872.33333	10087	170.2730419
spoIVCB	14515.33333	9598	529.1004028
spoIVFA	21239.66667	10845.66667	-2424.466375
spoIVFB	16511.33333	11511	1195.038198
spoVAA	14694	9236.666667	56.13959089
spoVAB	15619	9201	-557.4491899
spoVAC	13988.66667	9444	704.1515525
spoVAD	13286	9305.333333	1004.497432
spoVAEA	16033	10044.66667	27.55828189
spoVAEB	15348.33333	9281.333333	-308.0085569
spoVAF	15466.33333	8896.333333	-766.7326752
spoVB	14740.33333	9291.333333	81.8580868
spoVD	16699.33333	11364	930.5794328
spoVE	13347.33333	8324.666667	-14.48911502
spoVFA	22592.66667	11366	-2749.46128
spoVFB	15230	8412.666667	-1102.742845
spoVG	15300.66667	8096.333333	-1463.227345
spoVID	17254.33333	11957	1176.826164
spoVIF	13111.33333	7968	-223.707545
spoVK	23429.33333	11153.66667	-3484.527769
spoVM	19616.33333	10929.33333	-1326.572431
spoVR	15938.66667	8951.333333	-1006.837409
spoVS	26571.66667	15128.66667	-1472.796875
spoVT	20327.33333	11132.33333	-1567.791483
sppA	14718.66667	11191.33333	1995.395001
spsA	14493.66667	9929.333333	873.9706506
spsB	13508.5	9239.666667	799.817068
spsC	14046.5	9547.666667	771.6850708
spsD	15012	11050	1670.792673
spsE	13713	9953	1385.382756
spsF	13428.33333	9181	791.2369845
spsG	14576.33333	10841	1733.988782
spsJ	22750.33333	14141	-72.96836461
spsK	0	316.3333333	316.3333333
spsL	24318.5	14961.33333	-232.3952424
spxA	18924.5	6879	-4944.661675
sqhC	13313.66667	8123	-195.1214993
srfAA	17254.66667	10884.33333	103.9512375
srfAB	30544.33333	14974.33333	-4109.175526
srfAC	23154.66667	14978.66667	512.0786536
srfAD	15437.33333	9241.333333	-403.614036
ssbB	12692	9578	1648.283813
sspA	16457.33333	10797.66667	515.4430202
sspB	25674.5	15808.33333	-232.5978227
sspC	23566.5	14364.66667	-359.2268498

sspD	16418.33333	8620.666667	-1637.190534
sspE	16050.5	9757.666667	-270.3753797
sspF	14023	8845.333333	84.03408306
sspG	14616.66667	10620.33333	1488.122629
sspH	20384	11422.33333	-1313.19572
sspI	19066	11918	5.931861529
sspJ	7876.666667	4701.333333	-219.8556962
sspK	0	0	0
sspL	10863	10233.66667	3446.674314
sspM	15557.5	9560.333333	-159.6918457
sspN	24895	14326.66667	-1227.247962
sspO	0	0	0
sspP	24516.33333	12381.66667	-2935.66435
ssuA	13921.33333	10501.66667	1803.886784
ssuB	14765.33333	8979	-246.0947631
ssuC	17517	11044	99.71710995
ssuD	18855.5	11031	-749.5518087
stoA	33013.66667	14325.33333	-6300.967245
sucC	16067	10139.33333	100.982406
sucD	0	250	250
suhB	14670	8297	-868.5323398
sumT	16051.66667	10511	482.2290428
sunA	2642	1969	318.3294859
sunI	22459.33333	13914.66667	-117.4905247
sunT	120.5	1003	927.71393
swrAA	25029.33333	16121.5	483.6565023
swrC	16489.33333	10164.33333	-137.8832944
tadA	12007.5	7353.666667	-148.3871555
tagC	12900.33333	9834.666667	1774.787842
tasA	10765.66667	8217	1490.819632
tatAC	21309.33333	11773.33333	-1540.326095
tatAD	15573	9518.333333	-211.375946
tatAY	36670.33333	19869	-3041.915206
tatCD	22001.66667	10633	-3113.215908
tatCY	0	0	0
tcyA	14687	10235.33333	1059.179722
tcyB	16932.66667	9312.666667	-1266.536055
tcyC	15243	9186.333333	-337.1983269
tcyJ	14522.66667	8752.666667	-320.8146554
tcyK	16410.33333	9265	-987.8589553
tcyL	14775	9888.333333	657.1990238
tcyM	15598	9200	-545.328796
tcyN	13548.66667	9946.666667	1481.721711
tcyP	20719.5	12775.33333	-169.8096329
tdh	16592	9070.666667	-1295.694109
tdk	25074.33333	15373.66667	-292.2919609
tenA	23403.66667	14778	155.8416016

tenI	21038	11591.33333	-1552.802274
tepA	16416.33333	9359	-897.6076393
tetL	14043.66667	10312.66667	1538.455283
tgl	14524.33333	9452.333333	377.8107102
tgt	11692.33333	9029.666667	1724.522884
thiC	14400	8447.333333	-549.5082272
thiD	13480.33333	7628.666667	-793.5849433
thiE	15930.33333	12307.66667	2354.70243
thiF	15378	8985.333333	-622.5437166
thiG	14874	11093.66667	1800.679071
thiI	16998.66667	10826.66667	206.2284208
thiL	7188	6966.666667	2475.743254
thiN	15957	10995	1025.374946
thiO	0	0	0
thiQ	1588.333333	0	-992.3599545
thiS	18418.66667	10350	-1157.626789
thiT	22556.66667	10943.66667	-3149.302509
thiU	15011.66667	8104	-1274.999067
thiV	21593.66667	10463.33333	-3027.972063
thiW	189.6666667	68.33333333	-50.16673259
thiX	15674.33333	8250.666667	-1542.35372
thrB	14910	10481.33333	1165.853634
thrC	15592.33333	10391.33333	649.5449611
thrS	17269.66667	13163	2373.246194
thrZ	20148.33333	13323.33333	735.0442563
thyA	17049.33333	10388.33333	-263.7604662
thyB	23447.33333	15719.33333	1069.892846
tig	13810.33333	6839.333333	-1789.095896
tkt	18608.66667	8694	-2932.335115
tlp	22224.33333	12157	-1728.333735
tlpA	15771.66667	10426.33333	572.5009621
tlpB	16156	11468.33333	1374.376927
tlpC	0	368.6666667	368.6666667
tmrB	16060.66667	11155.66667	1121.272684
tnrA	19817	11206.33333	-1174.945084
topB	15681	10494.66667	697.4810756
tpx	16603.66667	10799.33333	425.6834497
treA	15106	8299	-1138.936709
treP	14254	8093.333333	-812.2902503
treR	15940	10454.66667	495.6628836
trmB	13496.33333	9456	1023.751899
trmE	23734.66667	13084.33333	-1744.627465
trmFO	11827.66667	8426	1036.302567
trmK	11489.66667	8669	1490.478432
trpA	18017	11330.33333	73.66011132
trpB	16840	10919.33333	398.0269528
trpC	18746.33333	12145	432.6534138

trpD	14893.5	11850	2544.829182
trpE	16376	10973	741.5918475
trpF	18644.5	13078.33333	1429.610245
trpP	16547.66667	10121.66667	-216.9954998
truA	15378.33333	8381	-1227.08531
truB	16685	10900.66667	476.201289
tuaA	13546.75	10314.5	1850.752541
tuaD	21604.5	14279.33333	781.2594795
tuaE	14685.5	10795	1619.78356
tuaF	13475	9956.333333	1537.413887
tuaG	439	1242.666667	968.3879552
tuaH	0	2150.666667	2150.666667
tyrA	24582	14409.33333	-949.0249474
tyrZ	16512.66667	11814.66667	1497.871823
ubiD	19992.33333	12975	484.1767065
ubiX	17752.66667	11320.66667	229.1438002
udk	15552.66667	11655	1937.994594
ugtP	20947.66667	9154	-3933.697088
ung	23578.33333	16331.66667	1600.379912
upp	10524.33333	9085	2509.600033
uppP	15564.33333	10267.66667	543.3721531
ureA	17056.66667	8983.666667	-1673.008858
ureB	13296.66667	8865	557.499772
ureC	19422.33333	8388	-3746.698315
usd	14637	6758.333333	-2386.581245
uvrA	17469.33333	11647	732.4983216
uvrB	20191.66667	13832	1216.637094
uvrC	16990.66667	11244	628.5599994
uvrX	16115	10687.33333	618.9929341
uxaA	15313	10658.33333	1091.067027
uxaB	14378.33333	8261	-722.3046462
uxaC	14259.33333	7785.666667	-1123.289081
uxuA	15593.66667	8618.666667	-1123.954746
uxuB	15651.33333	8391.666667	-1386.983765
veg	15963.33333	8860	-1113.581999
vmlR	22422.66667	13183.33333	-825.9152337
vpr	26380	16705.33333	223.6194189
walH	21588.33333	12876	-611.9732331
wapA	16808.33333	9279	-1222.52166
whiA	13802.66667	7783.333333	-840.3059107
wprA	0	0	0
xepA	0	71.66666667	71.66666667
xhlA	22717.66667	12315	-1878.558863
xhlB	129.6666667	805.6666667	724.6534406
xkdA	20520	11425	-1395.499224
xkdD	14859	8074.666667	-1208.949219
xkdE	20002.33333	9768.666667	-2728.404433

xkdF	16861.83333	9830.166667	-704.7807584
xkdG	17219.33333	9749.166667	-1009.139846
xkdH	15976.33333	9022	-959.7041472
xkdI	19685.33333	11678.33333	-620.6822963
xkdJ	14979.66667	10932.33333	1573.327248
xkdK	20901	11911	-1147.540657
xkdM	0	0	0
xkdN	21121	10131.66667	-3064.325736
xkdO	22013.33333	11512	-2241.505015
xkdP	16029.33333	11481	1466.182478
xkdQ	24455	13674.33333	-1604.677803
xkdR	27355	15581.33333	-1509.541728
xkdS	22676.33333	12089.66667	-2078.067929
xkdT	22780	12891	-1341.503524
xkdU	25068.33333	12611.33333	-3050.87661
xkdV	20486.66667	10427	-2372.673202
xkdW	19934.33333	9939.666667	-2514.919348
xkdX	452.6666667	0	-282.8173805
xkzA	15622.66667	9656	-104.7400523
xkzB	21393.66667	10864.66667	-2501.682597
xlyA	22062	12606	-1177.911008
xlyB	15901.33333	8651.666667	-1283.178931
xpaC	18986.33333	10015.66667	-1846.627279
xpf	0	27.33333333	27.33333333
xpt	13304.5	5848.666667	-2463.727677
xsa	23989.66667	13747	-1241.279867
xseA	13910.66667	9641.333333	950.2177776
xseB	18050.66667	12086.33333	808.625829
xtmA	24450.33333	13473	-1803.095493
xtmB	21663.33333	12138.33333	-1396.49845
xtrA	21798.33333	14182.33333	563.1561608
xylA	16089	8589.333333	-1462.762769
xylB	21080.66667	11753	-1417.792916
xylR	15190	8094.333333	-1396.084952
xynA	13053.33333	9473	1317.529734
xynB	16590.66667	9250	-1115.527735
xynC	16595.33333	10304.66667	-63.77671146
xynD	16095.66667	10087.66667	31.40536029
xynP	28389.66667	14394	-3343.314789
yaaA	12976.33333	9624	1516.637845
yaaB	13585.5	9114.333333	626.3756235
yaaC	26950.33333	14599.33333	-2238.71382
yaaH	24770.5	12898.33333	-2577.796103
yaaI	25041	12185	-3460.132605
yaaK	8365.5	7749	2522.397356
yaaL	0	0	0
yaaN	24967.33333	14036.33333	-1562.773763

yaaO	26487	15950.33333	-598.2321122
yaaQ	25357	13971.66667	-1870.896629
yaaR	13130.33333	8755.333333	551.7549557
yaaT	20836	12073	-944.9299136
yabA	22615.66667	16034.33333	1904.502098
yabB	27202.66667	14380.66667	-2615.033474
yabC	22646.66667	10641	-3508.199436
yabD	17362.66667	8866.333333	-1981.525074
yabE	26601	14267	-2352.790441
yabG	17447	9288.333333	-1612.21491
yabJ	17012.33333	9214.666667	-1414.310248
yabK	14501.33333	8554.666667	-505.4860012
yabM	21825.66667	12363	-1273.254511
yabN	32792	19037.66667	-1450.140865
yabO	22019	11943.66667	-1813.378772
yabP	6464.666667	3071.333333	-967.6653987
yabQ	14313.33333	9485	542.306097
yabR	15164.66667	11921.66667	2447.076158
yabS	15450	9937	284.1387423
yabT	13520	9316.666667	869.6320903
yacD	17759.33333	11865	769.3119291
yacL	13750.66667	9682.666667	1091.516017
yacP	21477	11485.33333	-1933.080986
yazA	17102.33333	9030.666667	-1654.540508
yazB	0	2058.333333	2058.333333
ybaC	16559.66667	11399.33333	1053.173799
ybaE	12947	7735.666667	-353.3685892
ybaF	20216.33333	12342.33333	-288.4408289
ybaJ	16094.33333	12140.33333	2084.905068
ybaK	31690.33333	21233.33333	1433.825833
ybaN	22103	17037.33333	3227.806319
ybaR	14752.66667	8847	-370.1808747
ybaS	15704	9113.666667	-697.8888797
ybbA	13681	9324.666667	777.0424035
ybbB	14679	10845.66667	1674.511301
ybbC	20372	11365.33333	-1362.698352
ybbD	21293.33333	11169.33333	-2134.329604
ybbE	15433	6525.333333	-3116.906653
ybbF	14816.33333	6608	-2648.958577
ybbH	15095	8656.333333	-774.7307887
ybbJ	28530.33333	13526.33333	-4298.867269
ybbK	14395.66667	9942.666667	948.532489
ybbP	13345.66667	9193.666667	855.5521861
ybbR	16069	11316	1276.399511
ybcC	14859	9234.333333	-49.28255199
ybcF	22915.66667	13849	-468.2654344
ybcH	0	0	0

ybcI	16192.33333	9857.666667	-258.9901039
ybcL	14696.33333	9401	219.0151027
ybcM	19570.66667	12960.33333	732.9592198
ybdG	20845	11925.33333	-1098.219606
ybdJ	13173.66667	9711	1480.347794
ybdK	17331	9605.666667	-1222.40702
ybdM	14023.66667	10539.33333	1777.617563
ybdN	0	471.6666667	471.6666667
ybdO	14275.66667	11820.66667	2901.506169
ybdZ	17234.66667	11440.66667	672.7801841
ybeC	15027.33333	11232.33333	1843.546036
ybeF	14465	11071.66667	2034.214363
ybfA	13670.66667	10562.66667	2021.49847
ybfB	15258.33333	10378.33333	845.2217029
ybfE	20028.33333	11457.66667	-1055.648731
ybfF	13344	8013.333333	-323.7398461
ybfG	17442.33333	9664.666667	-1232.965934
ybfH	14116.33333	11839.33333	3019.721221
ybfI	13876.66667	10941.33333	2271.46032
ybfJ	227	96.66666667	-45.15854405
ybfK	0	0	0
ybfM	13225.33333	8931.666667	668.734126
ybfN	23220.66667	12567.33333	-1940.490204
ybfO	17147.66667	12356.33333	1642.802768
ybfP	14930.66667	10602	1273.608167
ybfQ	13342.66667	10241.33333	1905.093195
ybgA	12208.66667	8552.333333	924.5944676
ybgB	15843.66667	10864.33333	965.5167543
ybgF	12782.33333	8719.666667	733.5119601
ybgG	13365	10128.66667	1778.473093
ybxA	13115.66667	9616	1421.585072
ybxB	17129.33333	11620.33333	918.2570807
ybxG	15758.66667	13108.33333	3262.623111
ybxH	15618	12088.33333	2330.508924
ybxI	18033	10597	-669.6697126
ybyB	13027.66667	9677.333333	1537.899104
ybzG	17740	11243.33333	159.7243552
ybzH	26547.66667	15345.66667	-1240.802139
ybzI	13437	10295.66667	1900.488885
ycbC	14277.66667	10577.66667	1657.256607
ycbD	19289	10460	-1591.394227
ycbG	18131	9147.333333	-2180.564884
ycbJ	16399	10507	261.2218922
ycbK	0	861	861
ycbL	12897.33333	9175	1116.995517
ycbM	12730	9399	1445.542148
ycbN	15367.66667	9390.333333	-211.0876497

ycbO	13879.33333	10115.33333	1443.794238
ycbP	16047	11946	1920.144686
ycbR	18432	10998.33333	-517.6238642
ycbU	14610.33333	9936	807.7462398
yccF	15880.66667	9216	-705.9334636
yccK	15752.33333	9145.666667	-696.0866118
ycdA	16732	8776.666667	-1677.163402
ycdB	15474.66667	11042.66667	1374.394153
ycdC	8237	4652.666667	-493.6516621
ycdF	18276.66667	10078	-1340.907934
ycdG	15506	10842	1154.151025
yceB	19238	11096	-923.5304127
yceC	12875	8909.666667	865.6156186
yceD	13406.33333	8877.333333	501.3154925
yceE	13514.33333	8529.333333	85.83918077
yceF	16322.66667	7316.333333	-2881.753184
yceG	14203.66667	9114.333333	240.1570431
yceH	15081	9828.666667	406.349474
yceI	17439	9138	-1757.549998
yceJ	16120	9698.333333	-373.1309692
yceK	12822.33333	9561.333333	1550.1874
ycgA	17279.33333	10763.33333	-32.46001892
ycgB	14917	11138	1818.146836
ycgE	14325.33333	8846	-104.191271
ycgF	22433.33333	12584.66667	-1431.246227
ycgG	16113.66667	11107.33333	1039.825975
ycgH	16617.33333	8858.333333	-1523.855219
ycgI	23114	13144	-1297.180266
ycgJ	0	0	0
ycgK	19646.66667	13346	1071.142556
ycgL	20416.66667	12442.33333	-313.6052219
ycgM	13299.33333	9990	1680.83369
ycgN	13582.66667	10726	2239.812502
ycgO	16113.33333	11019	951.7009019
ycgP	13893	9550.666667	870.5889027
ycgQ	9974.333333	7958.666667	1726.896064
ycgR	15494.33333	11549.66667	1869.1068
ycgS	15469	11313.33333	1648.601243
ycgT	17981.66667	11901.33333	666.7356948
yciB	18825.33333	10463.66667	-1298.037592
yciC	13908	10243.33333	1553.883859
yckA	18797.66667	12953.66667	1209.248006
yckB	21125	10680.66667	-2517.824859
yckC	18874.66667	11653.33333	-139.1934381
yckD	14451.33333	10159.33333	1130.419699
yclA	16970.66667	10723.33333	120.388946
yclD	14633	9045.666667	-96.74878861

yclE	26729	14191	-2508.762366
yclG	13134.33333	9667	1460.9225
yclH	13978.33333	7955	-778.3923806
yclI	23517.33333	12351.33333	-2341.841801
yclJ	12415	11493.33333	3736.681391
yclK	20980	12320.66667	-787.2316626
yclM	13120	9642.666667	1445.544356
yclN	17698	9329.666667	-1727.701524
yclO	20396	10455.66667	-2287.359755
yclP	0	161.3333333	161.3333333
yclQ	19988	11210	-1278.115911
ycnB	0	0	0
ycnC	12998	9968.666667	1847.767597
ycnD	18261.66667	12836	1426.463776
ycnE	12962	9965	1866.593034
ycnI	13005.66667	8552.666667	426.9776118
ycnJ	13018.66667	9272	1138.188797
ycnK	16451	8928	-1350.266702
ycnL	12575.33333	8908	1051.174891
ycsA	18933.33333	11547	-282.1805704
ycsD	22744	14611.33333	401.3219129
ycsE	18862.66667	11588	-197.0294035
ycsF	13261.33333	10355.33333	2069.908689
ycsG	12211.33333	8034	404.5950525
ycsI	16093.33333	10752.33333	697.5298485
ycsN	15370	9614.333333	11.45452874
ycxA	16081	9539.333333	-507.7645233
ycxB	20815.33333	13123.66667	118.6488868
ycxC	19863.33333	11919.66667	-490.5599212
ycxD	22573	14349.33333	246.1594065
yczC	18792.33333	9506	-2235.086497
yczE	17718.66667	11441	370.7196761
yczF	12929.33333	9451.333333	1373.335869
yczG	12724	10304	2354.290832
yczH	12645.66667	8951.333333	1050.565318
yczI	14440.66667	10726	1703.750692
yczJ	19481.33333	11882.33333	-289.2270409
yczK	12312.33333	8817.666667	1125.158872
yczL	15437	10565	920.2608909
yczM	12782	8593.666667	607.7202203
yczN	13602.33333	9329.666667	831.1918157
yczO	24032.66667	14186.66667	-828.4787693
ydaB	13556.66667	8793	323.0567993
ydaC	13812	9584.666667	955.1961365
ydaD	13878	9610.666667	939.9606127
ydaE	13994.66667	9710	966.4028685
ydaF	13350	10084.66667	1743.844803

ydaG	18765.66667	11746.33333	21.90765426
ydaH	13829	9538.333333	898.2415319
ydaJ	9506.666667	7061.333333	1121.751822
ydaK	11001.33333	8313.333333	1439.912989
ydaL	14800.33333	9633.666667	386.7045803
ydaM	14708.66667	10386.33333	1196.642808
ydaN	13858.66667	9849	1190.373039
ydaO	15815.66667	9368.333333	-512.9893871
ydaP	13319	9384.333333	1062.87967
ydaS	14147.33333	9097.333333	258.3530205
ydaT	21539.66667	11718.33333	-1739.233907
ydbA	19814.66667	12173.66667	-206.1539289
ydbB	16993.33333	10626	8.893917632
ydbC	14845.66667	8885.666667	-389.6188098
ydbD	27772.33333	17356.66667	5.049807807
ydbI	22763.33333	10543.66667	-3678.423847
ydbJ	17298.33333	8517.666667	-2289.997518
ydbK	21580.66667	12167.66667	-1315.516581
ydbL	14222.66667	9558.333333	672.2862105
ydbM	20659	11514.66667	-1392.677069
ydbN	14645.33333	10544.66667	1394.545583
ydbO	15208.33333	9282.666667	-219.2059306
ydbP	15711.33333	10622.66667	806.5293955
ydbS	14595.33333	9801.333333	682.4512831
ydbT	14681.66667	10415.33333	1242.511886
ydcA	11425.33333	8892.333333	1754.005988
ydcC	0	0	0
ydcF	11095	9377	2445.058534
ydcG	13697	10010.33333	1452.712579
ydcH	13066	9359.333333	1195.949178
ydcI	0	0	0
ydcK	14610.66667	10423.33333	1294.871313
ydcL	14188.33333	10227.66667	1363.070347
ydcO	19599	11455.33333	-789.742899
ydcP	19518.66667	11084.66667	-1110.218852
ydcQ	13642.33333	10675.66667	2152.200589
ydcR	14328.33333	10282.33333	1330.26772
ydcS	13154	10982.66667	2764.301813
ydcT	15429	11163.66667	1523.925803
yddA	15577.33333	11399.66667	1667.250004
yddB	12182.66667	9707.333333	2095.838765
yddC	16013.66667	13008.66667	3003.637375
yddD	13832	10727.33333	2085.36719
yddE	10636.33333	8951.333333	2305.957932
yddF	10979.66667	9317.333333	2457.449904
yddG	16649	9382.333333	-1019.63994
yddH	30118	18242	-575.1440362

yddI	21562	11904	-1567.520676
yddJ	19122	10160.66667	-1786.389189
yddK	26177.33333	15681.33333	-673.7583666
yddM	21698.66667	12512	-1044.907366
yddN	19377.33333	11035.33333	-1071.249852
yddQ	21510.66667	11705	-1734.448602
yddR	12294.66667	9724.666667	2043.196664
yddS	17688.33333	10871.33333	-179.9953105
yddT	15959	13614.33333	3643.458718
ydeA	20865	12418	-618.0485529
ydeB	19771.33333	13371	1018.253233
ydeC	14908.66667	10795.66667	1481.020008
ydeD	15656.33333	10982	1200.225665
ydeE	15919	9639.333333	-306.5500558
ydeF	12643.66667	9312	1412.481546
ydeG	16472	10279.66667	-11.72042957
ydeH	13814.66667	10036.66667	1405.530055
ydeI	12971.66667	10268.66667	2164.220154
ydeJ	12783	10111.66667	2125.09544
ydeK	14168.33333	11061.66667	2209.56596
ydeL	20541.66667	15670	2835.963862
ydeM	12981	9121	1010.722202
ydeN	0	0	0
ydeO	13538	9665.333333	1207.052705
ydeP	14257	9455.666667	548.168741
ydeQ	20111.66667	12035.33333	-530.0471194
ydeR	16741.66667	10690.66667	230.7970514
ydeS	12597	10021.66667	2151.304643
ydfA	25224	15639	-120.4674669
ydfB	15114.33333	10590	1146.856785
ydfC	16026	10500	487.2650799
ydfD	22885.33333	13552	-746.3137542
ydfE	13784.33333	9865.333333	1253.148402
ydfF	13282	9912.333333	1613.996555
ydfG	13797	10667.33333	2047.234513
ydfH	13983.66667	10509.66667	1772.942123
ydfI	22945.33333	10073.33333	-4262.467261
ydfJ	16933.66667	9609.666667	-970.1608361
ydfK	14242	10228.33333	1330.207118
ydfL	19565	12776.66667	552.8329769
ydfM	12862.66667	7374	-662.3454199
ydfN	12781.66667	9168	1182.261814
ydfO	13449	9521.333333	1118.658184
ydfP	15870.33333	10177.66667	262.1892699
ydfQ	13999	9970.666667	1224.362152
ydfR	21009.66667	13371	244.5665111
ydfS	12497	9756	1948.116043

ydgA	13569.33333	9715.666667	1237.809578
ydgB	15853	11800.66667	1896.018801
ydgC	18776.33333	13762.66667	2031.576661
ydgD	13896.33333	9553.666667	871.5063005
ydgE	18894.33333	14205	2400.185876
ydgF	17210	10107	-645.4752262
ydgG	15732	11078.66667	1249.617262
ydgH	20521.66667	12018	-803.5405249
ydgI	16699.66667	12321.33333	1887.704506
ydgJ	13609.33333	9355.333333	852.4850177
ydgK	21702	10661	-2897.989969
ydhB	16232	10406	264.5602631
ydhC	15712.33333	11379	1562.237948
ydhD	17397	9844.666667	-1024.642544
ydhE	0	0	0
ydhF	17365	10343.66667	-505.6495625
ydhG	17157	12289.33333	1569.971482
ydhH	27019.66667	16446.66667	-434.6986125
ydhI	15554.66667	11109.66667	1391.411699
ydhJ	16856.33333	10245.66667	-285.8444647
ydhK	13125.66667	9057	856.3372655
ydhU	6951.666667	4982.166667	638.8997513
ydiB	70.66666667	0	-44.15116692
ydiF	15540.66667	10373	663.4919621
ydiK	14009.33333	8838.666667	85.90608546
ydiL	13837.33333	9848.333333	1203.035026
ydiM	13590.66667	10151.66667	1660.480923
ydiR	20242.66667	12815.33333	168.1066136
ydiS	22351.33333	13875	-89.6808797
ydjA	20648.33333	12947.66667	46.98725764
ydjB	22656	12978.33333	-1176.697389
ydjC	22872.5	12568.33333	-1721.962402
ydjE	20442.33333	13634.33333	862.3587411
ydjG	15106.5	9926	487.7509004
ydjH	20370	12278.66667	-448.1154576
ydjI	23234	13849	-667.1539457
ydjJ	134	698	614.279391
ydjM	11570.5	7488.666667	259.6419947
ydjN	12901	8038.666667	-21.62867868
ydjO	14439	9353.666667	332.4586602
ydjP	22735.33333	16607.33333	2402.736679
ydzA	13437	9327.666667	932.4888855
ydzE	21774	12779	-824.9741764
ydzF	23192.33333	11622.33333	-2867.788085
ydzH	14372.66667	10480.66667	1500.902444
ydzJ	810.5	0	-506.3847281
ydzK	14715	10279.66667	1086.019197

ydzL	12634	10796	2902.521092
ydzM	12444	9967.666667	2192.896085
ydzN	18047.66667	11068.66667	-207.1664957
ydzO	12463.33333	10291.66667	2504.816992
ydzP	19113	11793.33333	-148.0994963
ydzQ	7580	6277	1541.162567
ydzR	20180.33333	12312.66667	-295.6153916
ydzS	15341.16667	10730	1145.135705
ydzT	17712.06667	12212.93333	1146.776562
ydzU	13193.33333	11302.66667	3059.727107
ydzV	12793	11944.33333	3951.5143
ydzW	16118.57143	11821.14286	1750.571098
ydzX	54.5	764.6666667	730.6161205
yeaA	16458	10792.66667	510.0264997
yeaB	15864	8870.666667	-1040.853786
yeaC	17057	10696.66667	39.78288203
yeaD	14164.66667	8487.333333	-362.476511
yebA	13268	8435.666667	146.0768177
yebC	17038.5	13096.66667	2451.341324
yebD	21457.66667	15318.33333	1911.998107
yebE	24335.33333	11320.33333	-3883.912384
yebG	18671.66667	13353.66667	1687.97037
yecA	16704	10525.66667	89.3304564
yeeA	15043.5	5955	-3443.887918
yeeB	20975.5	10913.33333	-2191.753483
yeeC	20679	11520.33333	-1399.506016
yeeD	19159.33333	12747.66667	777.2856662
yeeF	10996.33333	6536.666667	-333.6297741
yeeG	18194.5	11099.33333	-268.2384565
yeeI	15576	9499.333333	-232.250288
yeeK	15335	9428.666667	-152.3448147
yefA	18696.5	11307.33333	-373.8783498
yefB	0	0	0
yefC	16733.5	9707.666667	-747.1005732
yerA	16541	9211	-1123.496962
yerB	17667.66667	9405.666667	-1632.749843
yerC	21407.5	13591.33333	216.3412703
yerD	0	0	0
yerH	16868.33333	8966.333333	-1572.675166
yerI	19896.5	11419	-1011.94848
yerO	21151	11858	-1356.735823
yesE	14780	9573.666667	339.4084538
yesF	23200.5	13307.66667	-1187.557127
yesJ	15506.5	9563.666667	-124.4946985
yesK	16944	10074.66667	-511.6169029
yesL	20717	14039	1095.418985
yesM	16572.66667	10263.33333	-90.94834974

yesN	15634	9522	-245.8208999
yesO	15731.66667	9991.666667	162.825522
yesP	19800.33333	10455.33333	-1915.532073
yesQ	13856	8649	-7.960879401
yesR	17431	11028.33333	137.7815804
yesS	18374.5	11016.66667	-463.3656427
yesU	18499	11237.66667	-320.1508354
yesV	18213	11578	198.8697679
yesW	23598.66667	13992	-751.9906278
yesX	19703	11130.66667	-1179.386755
yesY	25128	12875	-2824.488523
yesZ	13795	8796.333333	177.4840744
yetA	16808	10479.33333	-21.98006599
yetF	18817.5	11797	40.18985651
yetG	20613.5	13080.33333	201.4171174
yetH	28836.5	15641	-2375.487615
yetI	17354.5	9968.666667	-874.0893655
yetJ	20269	11940.66667	-723.0126105
yetK	22209.33333	14704.33333	828.3713079
yetL	17346	11351.66667	514.2212702
yetM	15516.5	10216.33333	521.9241615
yetN	18265	12338.33333	926.7145067
yetO	24044	11580.66667	-3441.559617
yezA	25246	15192	-581.2126416
yezC	19850	11224.33333	-1177.562846
yezD	0	0	0
yezE	16240	9175	-971.4379822
yezF	16150.5	9702.333333	-388.1867795
yfhA	14191.33333	10570.66667	1704.196005
yfhB	16421	9723.333333	-536.189949
yfhC	21039	12756	-388.7603884
yfhD	20884.5	13180	131.7682242
yfhE	24857	15318.66667	-211.5062966
yfhF	0	0	0
yfhH	13548.5	9608.666667	1143.825841
yfhI	0	100	100
yfhJ	19125	10369.66667	-1579.263531
yfhK	20843	11455	-1567.303378
yfhL	15473.5	9936.333333	268.78973
yfhM	19768	11291	-1059.664165
yfhO	22411	14004.33333	2.373874019
yfhP	17783	12479.66667	1369.19212
yfhQ	20692	12911.66667	-16.29483135
yfhS	19020	10602	-1281.328228
yfiC	21956.33333	12399.66667	-1318.225851
yfiF	19083	11292.66667	-630.0227431
yfiG	14353	8823	-144.4768694

yfiH	19724.5	10532.33333	-1791.152872
yfiI	0	0	0
yfiJ	12375	8982	1250.339284
yfiK	19351	10596.33333	-1493.797294
yfiL	17226.33333	10851	88.32002294
yfiM	23207.5	12876	-1623.597258
yfiN	18697.5	11628.33333	-53.50313048
yfiQ	15616.5	9921	164.1127618
yfiR	13570.66667	9590.666667	1111.976537
yfiS	20830	12538.66667	-475.514563
yfiT	22858.5	13729	-552.5488064
yfiU	14152.66667	9566.333333	724.020857
yfiV	15590.5	9859.333333	118.6903923
yfiY	23504.33333	10703.66667	-3981.386319
yfiZ	16801	11485.66667	988.726732
yfjA	16476	10287.33333	-6.552885562
yfjB	0	0	0
yfjC	16237.33333	10719	574.2280996
yfjD	15178.5	8793.666667	-689.5666408
yfjE	13431.33333	7683.666667	-707.9706908
yfjF	16557	10449	104.5065473
yfjL	20763.66667	9052.666667	-3920.070779
yfjM	210.5	851.6666667	720.1503369
yfjO	17996.5	10474.33333	-769.5318851
yfjP	22187.5	9649.333333	-4212.987648
yfjQ	18278	10639	-780.7409753
yfjR	24149	12586.66667	-2501.161587
yfjT	26255.5	13082.33333	-3321.595388
yfkA	13849.66667	8355.666667	-297.3372685
yfkC	18504	10320.66667	-1240.274739
yfkD	19014.5	10414.33333	-1465.558601
yfkE	14446.5	9412	386.1061385
yfkF	0	0	0
yfkH	18893	11240.33333	-563.6477503
yfkI	16951.5	11207.66667	616.6972421
yfkJ	32190.33333	17756.66667	-2355.231165
yfkK	22220.33333	11779.66667	-2103.167946
yfkL	13141.33333	9065.666667	855.2157018
yfkM	22272.33333	13429.33333	-485.9898739
yfkN	15964.5	10703	728.6890907
yfkO	17267.5	10939	150.5998856
yfkQ	15262	9228.333333	-307.0691596
yfkR	20346.5	11370.33333	-1341.766445
yfkS	21335	14922	1592.304535
yfkT	16314.5	11013.33333	820.3491917
yflA	20305.66667	13122	435.4120985
yflB	17299	11520.33333	712.252628

yflD	15694	10729.66667	924.358927
yflE	8298.666667	6658	1473.15353
yflH	17374	10210.33333	-644.6059218
yflI	16356.33333	11801.66667	1582.545867
yflJ	15746	9173	-664.7963342
yflK	20153	11587	-1004.20472
yflL	18891	12958.66667	1155.935144
yflN	17448.5	11707.33333	805.8479188
yflP	17465	11153	241.2057045
yflS	14678.33333	8869	-301.7388454
yflT	15981	9607.666667	-376.9531236
yfmA	20432	12773.66667	8.148141268
yfmB	15925.5	9458.666667	-491.2777967
yfmC	15075.66667	8762	-656.9850292
yfmD	17914.66667	9946	-1246.737334
yfmE	18425.5	10614	-897.8961232
yfmF	0	0	0
yfmG	17438.5	11449	553.7623921
yfmI	18054	11085.66667	-194.1234399
yfmJ	13881	9626.666667	954.0862707
yfmK	14855.5	10296.33333	1014.90418
yfmL	15559.33333	8834.333333	-886.837277
yfmM	23277	13178	-1365.019514
yfmN	16741.5	9995.666667	-464.0988185
yfmO	13628.66667	10024.66667	1509.739258
yfmP	14328.5	9505	552.8302569
yfmQ	11657.5	7722.333333	438.9527436
yfmR	14861	9492.333333	207.4678867
yfmS	17607.33333	12508.33333	1507.611923
yfmT	22413.66667	15071.33333	1067.707792
yfnA	19388	11746.66667	-366.5808456
yfnB	15044	10619.33333	1220.133025
yfnC	14715	9320.333333	126.6858636
yfnD	15878	10993.33333	1073.065951
yfnE	16410.5	10432	179.0369146
yfnF	15020	9100.333333	-283.8722389
yfnG	13313.5	8820.333333	502.3159641
yfnH	19538	10950.66667	-1256.297945
yfzA	0	0	0
ygaB	19331	13180	1102.364986
ygaC	14370.5	11158	2179.589469
ygaD	17287.66667	10457.66667	-343.3331911
ygaE	18887	13097.66667	1297.434267
ygaF	15856	11384.66667	1478.144459
ygaJ	15327.5	6526.333333	-3049.992293
ygaK	20469.5	12244	-544.9478003
ygaN	16278.5	10007.66667	-162.8253711

ygaO	15245.66667	10355.33333	830.1355913
ygxA	20384.66667	10755	-1980.945574
ygxB	21182	10486.33333	-2747.77069
ygzA	22023.5	12026	-1733.856952
ygzB	20820	10482.33333	-2525.60009
ygzC	912	866.3333333	296.5333678
ygzD	17348.5	11180.33333	341.3259852
yhaA	17978.5	13171.33333	1938.714167
yhaH	19776.66667	10241.33333	-2114.745597
yhaI	20643	10725.66667	-2171.680579
yhaJ	15016.5	9412	29.98116011
yhaL	19153	13133.33333	1166.909277
yhaM	17786.5	11417.66667	305.0053877
yhaN	0	79.33333333	79.33333333
yhaR	17148	9995.666667	-718.0721584
yhaU	0	1096	1096
yhaX	26982	13237.33333	-3620.498541
yhaZ	16065.33333	8984.333333	-1052.976293
yhbA	0	0	0
yhbB	17066.33333	11010.66667	347.9515958
yhbD	14931	10118.66667	790.0665735
yhbE	15316.33333	9314.333333	-255.0155756
yhbF	0	1879.666667	1879.666667
yhbH	14019.5	8712.333333	-46.77918462
yhbI	0	0	0
yhbJ	15322	9414.333333	-158.5559994
yhcA	16200.66667	9807.666667	-314.1966094
yhcB	14973.5	10194	838.8467287
yhcC	14408.5	9787.666667	785.5144704
yhcD	21059	14425.66667	1268.410665
yhcE	21461	11002.33333	-2406.084495
yhcF	11223.5	7309.666667	297.4408851
yhcG	15545.5	8809.666667	-902.8611444
yhcH	14496.33333	10404.66667	1347.637902
yhcI	11495.33333	10216.66667	3034.604675
yhcJ	22041	10922	-2848.790614
yhcK	14888.33333	9363.333333	61.39054854
yhcM	15848	10938	1036.476038
yhcN	24574	14626	-727.3600354
yhcO	16132	10781.66667	702.7049962
yhcQ	17048	11513.33333	862.0725747
yhcR	28190.5	18187.66667	574.7873602
yhcS	15320	9334.333333	-237.3064381
yhcT	0	0	0
yhcU	15401.5	9927.666667	305.1072712
yhcV	16451	10229.33333	-48.93336896
yhcW	17667	10503.33333	-534.6666563

yhcX	19686	11334	-965.4321501
yhcY	17803	7720	-3402.97016
yhcZ	10973.66667	10036	3179.865254
yhdA	18118.5	10655	-665.0884594
yhdB	13583	8495	8.604241852
yhdC	20131.5	11902.66667	-675.1052692
yhdF	28209.5	16643.66667	-981.0834724
yhdH	16811.66667	10777.33333	273.7290716
yhdI	12824	9513.333333	1501.146099
yhdJ	17525	9785.333333	-1163.947802
yhdK	22297.66667	10154.33333	-3776.817651
yhdN	13250.66667	8571.666667	292.9063492
yhdP	17844.66667	10631	-518.0026876
yhdR	0	0	0
yhdT	19434	11277.66667	-864.3207561
yhdV	25769.5	12676	-3424.285319
yhdW	16158.5	10160.66667	65.14830857
yhdX	0	0	0
yhdY	19852.5	11351	-1052.458131
yhdZ	21180.5	13300.33333	67.16648098
yheA	0	0	0
yheB	7497	1859	-2824.980637
yheC	15892	10024	94.98568884
yheD	16213	10924	794.4310957
yheE	24934.5	13768.66667	-1809.926798
yheF	17988.5	10230.33333	-1008.53364
yheG	16766.5	10263.33333	-212.0516684
yheH	15229.66667	9401.333333	-113.8679181
yheI	15773.66667	9251.333333	-603.7485993
yheJ	20497.5	12547.66667	-258.7749922
yheN	0	0	0
yhfA	18375	11540.66667	60.32196697
yhfC	0	279.3333333	279.3333333
yhfE	16926.5	10636.66667	61.31675867
yhfF	13967	9127.666667	401.3551336
yhfH	17130	10910	207.5072269
yhfI	15776.5	10975.66667	1118.814522
yhfJ	16489	10627	324.9916325
yhfK	15969.5	11382	1404.565187
yhfM	12490	8486.666667	683.1561742
yhfN	14342.5	9491.333333	530.4166609
yhfO	14155	9628.333333	784.5630354
yhfP	21876.66667	12050	-1618.118325
yhfQ	21859.33333	12289.66667	-1367.622126
yhfS	294.6666667	918.3333333	734.2312977
yhfT	0	0	0
yhfW	0	1404.666667	1404.666667

yhgB	2256	0	-1409.505178
yhgC	16812.5	9859	-645.1249123
yhgD	12147	8320	730.7892753
yhgE	17591.5	12246	1255.17095
yhjA	0	0	0
yhjB	15036.33333	9834.333333	439.9230103
yhjC	0	0	0
yhjD	18857.66667	12590.33333	808.4278332
yhjE	16366.33333	10313.66667	88.29806058
yhjG	0	0	0
yhjH	20032.5	11680	-835.9186502
yhjM	0	0	0
yhjN	16600	10448.66667	77.30764544
yhjO	18357.33333	11789.33333	320.0264254
yhjP	17341.66667	9786.333333	-1048.40468
yhjQ	0	36.33333333	36.33333333
yhjR	14755	10264.33333	1045.694637
yhxA	16999.66667	10518.66667	-102.3963599
yhxC	12300.5	7967.333333	282.2187767
yhxD	0	0	0
yhzB	17720	11156.66667	85.55330184
yhzC	0	0	0
yhzD	15584.5	9625.666667	-111.2275903
yhzE	30317	17315.66667	-1625.808722
yhzF	19460	10126	-2032.23172
yhzG	18725.66667	11647	-52.43445251
yisB	13736.5	8493.666667	-88.63292339
yisI	26866	14060	-2725.357317
yisJ	15579.5	7283.333333	-2450.43702
yisK	14900.33333	9066.333333	-243.1068194
yisL	13415.5	8529	147.2550031
yisN	15364	9835	235.8698794
yisP	0	0	0
yisQ	0	933	933
yisR	23166.5	12528.33333	-1945.647918
yisS	0	0	0
yisT	135	0	-84.34538963
yisU	18465.5	10197	-1339.88735
yisV	0	1056.333333	1056.333333
yisX	0	0	0
yisY	0	0	0
yisZ	0	2248	2248
yitA	14807	9258.333333	7.206042536
yitB	0	175	175
yitC	0	0	0
yitD	16485.5	10207	-92.8216352
yitE	18732.5	10484.33333	-1219.370454

yitF	18348.5	11073.66667	-390.1213454
yitG	16572	9001	-1352.865163
yitH	22611.5	13721	-406.2279824
yitI	25701	14080.66667	-1976.821177
yitJ	22287.5	12418.66667	-1506.132381
yitK	0	0	0
yitL	14609	8777	-350.4207193
yitM	0	0	0
yitO	16634	10171	-221.6015638
yitP	0	43.66666667	43.66666667
yitQ	0	0	0
yitR	18384	10617.66667	-868.301059
yitS	713.5	0	-445.7810037
yitT	20178.33333	11894	-713.032497
yitU	0	0	0
yitV	15086.5	9628	202.2465136
yitW	0	0	0
yitY	20992.5	9661.666667	-3454.041421
yitZ	12330	8488.666667	785.1210804
yizA	0	0	0
yizB	16418.5	10467	209.0386693
yizC	21346	13972.66667	636.0986144
yizD	20958	13202.66667	108.513512
yjaU	17097	10831.33333	149.4583221
yjaV	0	0	0
yjaZ	18536.66667	10415.66667	-1165.68424
yjbA	15294	10891	1335.604526
yjbB	17470	8823.333333	-2091.584866
yjbC	22624.5	11787.33333	-2348.016798
yjbE	19543	10513.33333	-1696.755182
yjbH	20876.5	11561.33333	-1481.900197
yjbI	14351.66667	9445	478.3561715
yjbJ	16437.66667	8406.666667	-1863.269627
yjbK	20506	11660.33333	-1151.418961
yjbL	10650.33333	5671.333333	-982.7889977
yjbM	16965	9070.666667	-1528.737297
yjbO	15755	9142.666667	-700.7526935
yjbQ	16208	9928	-198.445001
yjcA	18491.66667	10032.33333	-1520.902444
yjcD	28419.33333	15931.33333	-1824.516615
yjcF	19473.66667	10667.66667	-1499.103722
yjcG	19817.33333	11401.66667	-979.8200106
yjcH	23069.66667	11828.66667	-2584.81499
yjcK	16704.66667	8579	-1857.752731
yjcL	22150.33333	13993.66667	154.5667004
yjcM	16794	8686.333333	-1806.233137
yjcN	13657	8296	-236.6295273

yjcO	15728.33333	8369.666667	-1457.091876
yjcP	18895.66667	10394	-1411.647165
yjcQ	0	0	0
yjcR	21810.33333	11047.33333	-2579.341207
yjcS	22367	12712	-1262.46911
yjcZ	15985.33333	8508.333333	-1478.99384
yjdA	24593.33333	12897	-2468.439128
yjdB	20638.33333	10388.5	-2505.931602
yjdF	15847.66667	9527.666667	-373.6490351
yjdG	16303.66667	8889.666667	-1296.549018
yjdH	22905.66667	11549	-2762.017628
yjdI	18161.33333	8115.333333	-3231.516565
yjdJ	18814.33333	9706.666667	-2048.165005
yjeA	21344.66667	12266	-1069.735011
yjfA	14043.66667	7886	-888.211384
yjfB	15717.66667	8542.666667	-1277.427549
yjfC	15063.66667	8343.666667	-1067.820995
yjgA	16966.66667	8935	-1665.445265
yjgB	17103.33333	8923.666667	-1762.165289
yjgC	25678	13229.66667	-2813.451222
yjgD	21181.66667	10092.33333	-3141.56243
yjhA	18103.33333	10469.66667	-840.9459527
yjhB	13255.66667	7724.333333	-557.5508875
yjiA	16271	8671.333333	-1494.472849
yjiB	22543.33333	11116.33333	-2968.305434
yjiC	22738.66667	13052.33333	-1154.345924
yjjA	19797	9106.333333	-3262.44947
yjkA	15951	9581.333333	-384.543037
yjkB	21253	11164.66667	-2113.796784
yjlA	22308.33333	11729	-2208.815311
yjlB	22295.66667	10966.66667	-2963.234756
yjmC	15680	8382.666667	-1413.894144
yjmD	17137.33333	8860.333333	-1846.741165
yjnA	22552.33333	10752.33333	-3337.92846
yjoA	15510.66667	8547.666667	-1143.097951
yjoB	13320.66667	7539.333333	-783.1616306
yjpA	0	0	0
yjqA	27272.66667	13499	-3540.434787
yjqB	21208.66667	11803	-1447.764841
yjqC	26582	15677	-930.9196086
yjzB	14523	8880	-193.6895822
yjzC	17297.5	10852.33333	45.18979902
yjzD	17195	11271.33333	528.2298171
yjzE	187.6666667	494.6666667	377.4161621
yjzF	16077.66667	11059	1013.984746
yjzG	20334	12334.33333	-369.956687
yjzH	20793.33333	11927	-1064.272605

yjzI	21618.66667	11081	-2425.924913
yjzK	13309.33333	7622.333333	-693.0807831
ykaA	0	0	0
ykbA	86	0	-53.7311371
ykcB	13815.66667	7416	-1215.761393
ykcC	678	114.6666667	-308.9346235
ykfA	15989.33333	9358	-631.8262958
ykfB	20547.66667	11748.33333	-1089.451489
ykfC	74.66666667	584.6666667	538.0163771
ykfD	19540.33333	11565.66667	-642.7557667
ykgA	15766.33333	9292.666667	-557.8335411
ykgB	14724	8708.666667	-490.603829
ykhA	20548.66667	11182.66667	-1655.742936
ykjA	12878.66667	7589.666667	-456.6752439
ykkA	15800.66667	8783	-1088.951011
ykkB	20785.33333	11472.33333	-1513.941027
ykkC	15596.66667	9230	-514.4957551
ykkD	15876	9533.666667	-385.3511539
yknT	0	1375.666667	1375.666667
yknU	20098.66667	10521.66667	-2035.591637
yknV	20627	11183.33333	-1704.017422
yknW	13277.66667	8028.333333	-267.2960621
yknX	17116.66667	11112.33333	418.1709691
yknY	24928.66667	11612	-3962.948911
yknZ	32244	16530.33333	-3615.094394
ykoA	21161.66667	12200.33333	-1021.066817
ykoG	16809.33333	9046	-1456.14644
ykoH	117.6666667	178.3333333	104.8174752
ykoI	15855.66667	7491.666667	-2414.64728
ykoJ	15364.66667	8692	-907.546641
ykoL	16919.33333	9138.333333	-1432.53898
ykoM	21484.33333	10208.33333	-3214.662711
ykoN	12945.33333	7810.666667	-277.3272881
ykoP	11275	7298.666667	254.2646809
ykoQ	15797	8734.666667	-1134.993481
ykoS	16627.33333	11185.66667	797.2303073
ykoT	17801.33333	9937.333333	-1184.595525
ykoU	16453.33333	9659.666667	-620.0578572
ykoV	13866	7909.666667	-753.5420194
ykoW	20568.33333	10996	-1854.696956
ykoX	23738.33333	12360	-2471.251661
ykoY	17895	10725	-455.449981
ykpA	17337.33333	11269.66667	437.6360359
ykpC	23754.33333	12331	-2510.248151
ykqA	15708.66667	9136.333333	-678.1378561
ykrA	18327.33333	9638	-1812.563488
ykrK	23018.66667	11833.66667	-2547.951176

ykrP	18015	9733	-1522.423661
yktA	19816	14363	1982.346364
yktB	25178.33333	13664.33333	-2066.602483
yktD	15257	8372	-1160.27859
ykuC	20574.66667	10996.33333	-1858.320567
ykuD	17377.66667	9365	-1492.230118
ykuE	15292.66667	8721	-833.5624332
ykuH	14105.66667	9161	348.0522148
ykuI	16258.33333	9660	-497.8922944
ykuJ	15606.33333	9325	-425.5353015
ykuK	21552.66667	13231.33333	-234.3560561
ykuL	16144.33333	9717	-369.6672987
ykuN	14212.33333	8376	-503.591056
ykuO	21083.66667	10189	-2983.667258
ykuP	0	0	0
ykuS	27220.66667	13675.66667	-3331.279526
ykuT	18555.33333	11617.66667	24.65318724
ykuU	28759.66667	12162	-5806.483634
ykuV	0	0	0
ykvA	18284.33333	9143	-2280.69792
ykvI	22232.33333	11326	-2564.331981
ykvN	23708.33333	14281.66667	-530.841574
ykvO	21508.33333	10243.66667	-3194.324113
ykvP	14515.66667	8744.666667	-324.4411907
ykvQ	12158	7371	-225.083312
ykvR	21641.33333	10704.33333	-2816.753275
ykvS	22260	13715.66667	-191.9509124
ykvT	0	0	0
ykvU	15866.66667	9850.333333	-62.85320101
ykvY	17064.66667	8971.333333	-1690.340436
ykvZ	21114.33333	11066	-2125.827198
ykwB	17261.66667	10661.66667	-123.0888939
ykwC	21296.33333	11152.66667	-2152.870613
ykwD	18125.66667	10436.66667	-887.8993875
ykyA	22017.66667	12059.66667	-1696.545732
ykyB	16407	9336	-914.7763531
ykzB	12802.33333	8109.666667	111.0163468
ykzC	15396.33333	8292	-1327.331362
ykzD	26078.33333	14390.33333	-1902.905081
ykzE	0	0	0
ykzF	16682.33333	8845.333333	-1577.465963
ykzG	14327.33333	7007.333333	-1944.107499
ykzH	21694	12435.33333	-1118.65839
ykzI	23371.66667	12243	-2359.165417
ykzK	13039	10497.33333	2350.818256
ykzL	19213.33333	12506	501.8808437
ykzM	17434	11399	506.5739051

ykzN	17722	10275	-797.3629262
ykzO	131	0	-81.84626697
ykzP	11865.66667	7667.333333	253.8942354
ykzQ	19712.66667	10867	-1449.092968
ykzR	24237.66667	11236	-3907.225472
ykzS	24380.66667	12797	-2435.569107
ykzT	17226	9275.166667	-1487.30505
ykzU	22293.33333	10540	-3388.443601
ykzV	17466.33333	9621	-1291.627336
ykzW	20711.33333	10660	-2280.040591
ylaA	15288.66667	8284.333333	-1267.729977
ylaB	15410	11099.66667	1471.796636
ylaC	19883.33333	9788	-2634.722201
ylaD	18037.66667	12171.33333	901.7479776
ylaE	25573.33333	12678.33333	-3299.390846
ylaF	23977	9747.333333	-5233.032646
ylaH	17257.66667	9200.333333	-1581.923105
ylaI	15039	7901.333333	-1494.743071
ylaJ	15470	10468.33333	802.9764624
ylaK	13273.33333	8907.333333	614.4113208
ylaL	9864	7495	1332.163531
ylaN	17129.33333	9089.333333	-1612.742919
ylbA	16884.66667	8877	-1672.21325
ylbB	17618	9340.666667	-1666.71907
ylbC	22585.33333	10756.66667	-3354.212888
ylbD	14670	10903	1737.46766
ylbE	16879	9226.666667	-1319.00616
ylbF	14381.33333	7218.333333	-1766.845655
ylbG	20699.33333	10155.66667	-2776.876556
ylbH	18366	9917.333333	-1557.38834
ylbJ	12837.66667	7840.333333	-180.39257
ylbK	19941	10551.66667	-1907.084553
ylbL	12926.33333	9701.333333	1625.210211
ylbM	12836	7494	-525.6846022
ylbN	0	0	0
ylbO	17099	9188	-1495.124573
ylbP	18035.33333	9179.333333	-2088.794201
ylbQ	15362	6731	-2866.880559
yllA	19256.66667	10618	-1413.192985
ylmA	14833.66667	9511.666667	243.8785582
ylmC	10962.66667	6948.666667	99.40450823
ylmD	14970	9510	157.033461
ylmE	15520.33333	9275	-421.8041644
ylmG	17149.66667	10308	-406.7801262
ylmH	17226.66667	10443	-319.8882373
yloA	33039	16970	-3672.128356
yloB	12525.66667	9071.666667	1245.872331

yloC	11172.66667	7988	1007.533902
yloN	14349.66667	9869.666667	904.2723995
yloU	0	48	48
yloV	0	468.3333333	468.3333333
ylqB	12301.33333	9040.666667	1355.031459
ylqC	14992	10231.66667	864.954953
ylqD	28375	16791.33333	-936.8180057
ylqG	13639.5	9147.666667	625.970801
ylqH	26061.33333	13877	-2405.617143
ylxF	15514.66667	11539	1845.736259
ylxH	15138.33333	6979.333333	-2478.804617
ylxL	12042.33333	9825	2301.182985
ylxM	24405.66667	13977.33333	-1270.85529
ylxP	15949	9229.333333	-735.2934757
ylxR	14063.66667	9865.666667	1078.959669
ylxS	12950	11840	3749.090402
ylxW	8724.666667	8129	2677.996967
ylxX	17564	10203.66667	-769.9809146
ylxY	20209	11383	-1243.192437
ylyA	16972	10670.33333	66.55590513
ylzA	24621	13591	-1791.724727
ylzH	13342.66667	6244	-2092.240139
ylzI	15711.33333	11561.33333	1745.196062
ylzJ	17944.66667	10299.66667	-911.8140873
ymaB	15104.33333	9286	-150.8954082
ymaC	24026.66667	12490.66667	-2520.730085
ymaD	19776.33333	10980.33333	-1375.537337
ymaE	14819.66667	8732.333333	-526.7078459
ymaF	6300.666667	2309.333333	-1627.20137
ymaG	15222.66667	7871	-1639.827787
ymaH	18749.66667	9642	-2072.429188
ymcA	14723.66667	6720	-2479.062235
ymcB	15228.33333	8145.333333	-1369.034877
ymcC	20127.66667	10648.33333	-1927.04361
ymdA	53.33333333	0	-33.32163541
ymdB	15220	9316.333333	-192.8283717
ymfC	15951.66667	9046	-920.2928908
ymfD	26790	19044.33333	2306.459347
ymfF	26540	14725	-1856.678821
ymfH	13623.33333	8508.333333	-3.261911594
ymfI	12578	6687.666667	-1170.824524
ymfJ	16923.66667	9510.333333	-1063.246363
ymfK	15920.16667	10123.16667	176.5543668
ymfM	17959.66667	9841	-1379.852464
ymxH	2717.333333	707	-990.7373241
ymzA	23923	10762.33333	-4184.29449
ymzB	14614	8126	-1004.544623

ymzC	26895.66667	14385	-2418.892477
ymzD	15700.33333	8621	-1188.264684
ymzE	18367.33333	9551.666667	-1923.888048
ynaB	15412.33333	8973.666667	-655.661186
ynaC	18602	9135.666667	-2486.503244
ynaD	14043.66667	8634.333333	-139.8780507
ynaE	16391.66667	9730.333333	-510.8630496
ynaF	20132.66667	11828.33333	-750.1675133
ynaG	17475.66667	9447.666667	-1470.791956
ynaI	17332.66667	9170.333333	-1658.781654
ynbA	23639.33333	9990.666667	-4778.731708
ynbB	15721.66667	8102.666667	-1719.926671
yncB	16770.33333	10947.33333	469.553339
yncC	16289.33333	9212.333333	-964.9271616
yncE	18143	9364	-1971.395586
yncF	32016.33333	19330.33333	-672.8526632
yncM	14460.66667	8682	-352.7449209
yndA	16217.33333	9735.333333	-396.9429538
yndB	20333.33333	11282.66667	-1421.206833
yndD	21090.66667	12245.66667	-931.374056
yndE	17235.66667	9440.333333	-1328.17793
yndF	17855.66667	9517.666667	-1638.208608
yndG	17466.33333	9504.666667	-1407.96067
yndH	15554.66667	10776.66667	1058.411699
yndJ	18634.33333	10109.33333	-1533.037819
yndK	23650.33333	14501.66667	-274.6042955
yndL	765.6666667	0	-478.3737283
yndM	21550.66667	12223	-1241.439828
yneA	15918.33333	9105.666667	-839.800202
yneB	0	106.3333333	106.3333333
yneE	21491.33333	12055.33333	-1372.036175
yneI	17774.33333	10603	-502.0597809
yneJ	18941.66667	9370.333333	-2464.053743
yneK	19756	10715.66667	-1627.50013
yneN	25575.33333	14418.66667	-1560.307074
yneP	14148.66667	10599	1759.186646
yneQ	18297.66667	11181.66667	-250.3616617
yneR	22187	11628.66667	-2233.341924
yneT	24393.66667	13773.66667	-1467.024589
ynfC	17757.33333	11178.33333	83.89482372
ynfE	17003.33333	9207.333333	-1416.020556
yngA	16692.66667	10277.33333	-151.9220294
yngB	21088.66667	11577	-1598.791161
yngC	16672	10160.33333	-256.0098957
yngD	11123	8279.666667	1330.231342
yngE	13066.33333	9065	901.4075849
yngF	12747.66667	9257	1292.504356

yngG	12909.66667	9893	1827.289889
yngHA	11967.66667	9492.666667	2015.499941
yngHB	13204	10489.66667	2240.06278
yngI	12461.66667	10860.33333	3074.52496
yngJ	17471	10570.33333	-345.2096462
yngK	13505	11095.66667	2658.0038
yngL	35791.33333	23300.33333	938.6003304
ynxB	19567.66667	9463	-2762.499772
ynzB	21672.66667	12443.33333	-1097.329736
ynzC	17455.66667	11549.33333	643.370324
ynzD	22270	13295.33333	-618.5320524
ynzE	12980	8703.333333	593.6803155
ynzF	0	71.33333333	71.33333333
ynzG	25697.33333	15705	-350.1969812
ynzI	23322	11853.33333	-2717.801311
ynzJ	17730.66667	9412.666667	-1665.111025
ynzK	9385.333333	4857.666667	-1006.108125
ynzL	27970	16269.66667	-1205.448503
yoaA	13507	9052	613.0875723
yoaB	16894.33333	9595.666667	-959.58613
yoaC	13360	8878	530.9303299
yoaD	24863.66667	13097.66667	-2436.671501
yoaE	15167.66667	10501	1024.53515
yoaF	13846.33333	9659	1008.078667
yoaG	14046	9714	938.3307945
yoaH	19331	10938	-1139.635014
yoaI	12811.33333	9260	1255.726654
yoaK	13779.33333	7935.666667	-673.3943618
yoaM	19636.33333	11573.66667	-694.7347105
yoaN	14269.66667	10008	1092.588186
yoaO	13998.66667	10577	1830.903746
yoaP	22578.66667	13684.66667	-422.047684
yoaQ	14862.33333	12064.33333	2778.634846
yoaR	34774.33333	17604.33333	-4121.997734
yoaS	17485	9594.666667	-1329.623242
yoaT	13868.33333	9501	836.3334924
yoaU	15576.66667	10043	310.9998582
yoaW	15413	10954.66667	1324.922294
yoaZ	16580	10530.33333	171.4699254
yobA	21334.66667	10194.33333	-3135.153871
yobB	15729.66667	10720.33333	892.74175
yobD	14468	9852.333333	813.0066876
yobE	20685.33333	11844.33333	-1079.46296
yobF	15036	10808	1413.797937
yobH	18741	11178.66667	-530.347756
yobI	22900	13880	-427.477204
yobJ	17363	10294.66667	-553.4000011

yobK	15597	10461.66667	716.9626514
yobL	21045.33333	11941.33333	-1207.383999
yobM	14737.66667	10409.33333	1201.524169
yobN	15887	11468.33333	1542.442925
yobO	16538.33333	10616.33333	283.5024531
yobQ	14880	9902.333333	605.5970541
yobR	14351.33333	10467.33333	1500.897765
yobS	14893	10772	1467.141572
yobT	18035.33333	13136.66667	1868.539132
yobU	18782.33333	10969.66667	-765.1720235
yobV	14329	10145.66667	1193.184533
yobW	15313.66667	11205	1637.317173
yocA	18706.33333	11861.33333	173.9779737
yocB	17678.33333	10517.33333	-527.7475038
yocC	12547.33333	8724	884.6687495
yocD	14025.66667	10158.33333	1395.368001
yocH	14288.33333	8512.666667	-414.4077198
yocI	22499	12938.33333	-1118.606824
yocJ	19879.33333	12360.33333	-59.88974513
yocK	1778.333333	414.3333333	-696.7349474
yocL	16708	11595.66667	1156.831334
yocM	16038.33333	11435.33333	1414.892785
yocN	13377.33333	9814	1456.100798
yocR	13122.33333	8379.333333	180.753201
yocS	16700.33333	9624.666667	-809.3786812
yodA	13847	9822.333333	1170.99548
yodB	15115	10224	780.4402647
yodC	13610.5	10508.33333	2004.756107
yodD	17710.5	11869	803.8220515
yodE	12810	8823.666667	820.2263617
yodF	13655	9330	798.620034
yodH	12646	9414.666667	1513.690391
yodI	12007.5	9303.666667	1801.612845
yodJ	14141.66667	8541.666667	-293.7732224
yodL	14114.5	9415.666667	597.1999856
yodM	12761.5	8749	775.8615573
yodN	11963	8340.666667	866.4155841
yodP	15521	8943	-754.2206848
yodQ	18220.5	11846.33333	462.5172462
yodR	12932	8838.666667	759.0031207
yodS	14690	9662	483.9720469
yodT	18864.5	12733.66667	947.491832
yoeA	17388	9575	-1288.686184
yoeC	16439.66667	9547.666667	-723.5191881
yoeD	14059.33333	10396	1612.000386
yogA	12528.33333	10559.33333	2731.872915
yojA	13244	9566.666667	1292.071554

yojB	19649	12215.33333	-60.98193221
yojE	15468.5	10186	521.5803
yojF	13058	9637	1478.61409
yojG	18299.5	12322	888.8262404
yojJ	12821.5	9455.666667	1445.041384
yojK	16698	10332.33333	-100.254193
yojN	14133.5	9695.333333	864.9958197
yojO	13140.5	9398.333333	1188.403019
yojW	14850	12098.66667	2820.673807
yokA	22135.5	13924.66667	94.83428026
yokB	19459	12774	616.3930606
yokC	17198	9839.666667	-905.3111916
yokD	15578	10540	807.1668173
yokE	16565.33333	9347.333333	-1002.366625
yokF	16918.66667	11221.66667	651.2108739
yokG	229.5	0	-143.3871624
yokH	15437	6785.333333	-2859.405776
yokI	14077.5	10137	1341.650204
yokJ	13450.5	9863	1459.38768
yokK	14677.66667	8743.666667	-426.6556583
yokL	13199	10820.33333	2573.85335
yokU	10942.66667	8464.333333	1627.566788
yolA	1115.5	0	-696.9428306
yolB	17806	12376.33333	1251.488831
yolC	14406.5	9728.333333	727.4306984
yolD	17072	9770.666667	-895.5888279
yolJ	14428.33333	10273.66667	1259.122987
yomD	15035.5	11125.66667	1731.776994
yomE	17464	10425.33333	-485.8361815
yomF	0	0	0
yomG	18355.5	12182	713.8385233
yomH	14629	11070	1930.083667
yomI	0	3518.333333	3518.333333
yomJ	336.5	2143.333333	1933.09464
yomK	17813.33333	10229.33333	-900.0928935
yomL	15845.33333	9690.666667	-209.1912135
yomM	23568.33333	15702	976.9610523
yomN	15406	10276.66667	651.2957582
yomO	19576	13286	1055.293723
yomP	14899.5	10704	1395.080498
yomQ	20782.66667	14134	1149.391722
yomR	15298.5	10523.33333	965.1263462
yomS	14341	10162	1202.020499
yomT	16689	11523.33333	1096.368833
yomU	13551.66667	10513.33333	2046.514036
yomV	13765.5	9615.666667	1015.248437
yomW	12966	9533.666667	1432.760578

yomX	15905	11804.33333	1867.196874
yomY	18295	13081	1650.637753
yomZ	15370	10488.33333	885.4545287
yonA	14918	9916	595.5220555
yonB	18619.5	11234	-399.103572
yonC	15899	10590.33333	656.9455575
yonD	1469	930.3333333	12.53053802
yonE	14828	8582.333333	-681.9143514
yonF	9911.5	8154.666667	1962.153116
yonG	19812.33333	14466.33333	2087.970559
yonH	19672	11476.33333	-814.3518875
yonI	19363	13172	1074.372004
yonJ	12210	8106.333333	477.7614268
yonK	16573	10759.66667	405.1767234
yonN	0	1044.666667	1044.666667
yonO	13821.66667	9596.333333	960.8232567
yonP	23404	15148.33333	525.9666747
yonR	19511.33333	11317.33333	-872.9704608
yonS	17858	9562.666667	-1594.66643
yonT	17365	10939.33333	90.01710421
yonU	13987.5	9683.666667	944.54713
yonV	16900.66667	10287.33333	-271.8764075
yonX	14846	9739.666667	464.17293
yopA	13702.5	9422.666667	861.6096192
yopB	13730.5	9849	1270.449094
yopC	18291.5	12980.33333	1552.157819
yopD	13674.5	10408.66667	1865.103478
yopE	12552.5	9068.333333	1225.774049
yopF	12484	9096.333333	1296.571525
yopG	12945	9368.333333	1280.547639
yopH	14961.33333	9328.333333	-19.21843993
yopI	12479.5	9161.333333	1364.383038
yopJ	15040.33333	10888.66667	1491.757221
yopK	16605.66667	9461.666667	-913.2327783
yopL	12791	9531.333333	1539.763861
yopM	15835	9375.333333	-518.06848
yopN	17059	13364.33333	2706.199987
yopO	12522	9719.666667	1896.163193
yopP	12787.66667	8484.333333	494.8464632
yopQ	11641	11311	4037.928291
yopR	315	0	-196.8059091
yopS	18071	12356	1065.588622
yopT	25071.33333	16177.33333	513.2490477
yopU	13256	9327.666667	1045.574186
yopV	12645.5	8844	943.3361143
yopW	14938	15046.66667	5713.693109
yopX	12155	9556.666667	1962.457697

yopY	10150.33333	11492.66667	5150.934668
yopZ	14074.5	9764.333333	970.8578789
yoqA	25865	14987	-1172.951873
yoqB	20199.5	13405.33333	785.0763123
yoqC	9008	6744.333333	1116.309113
yoqD	27083	18008.33333	1087.398612
yoqE	22223	12686.33333	-1198.167361
yoqF	24499	16864.66667	1558.165181
yoqG	24758.33333	16148.66667	680.1387289
yoqH	25634.33333	18604.33333	2588.497534
yoqI	16879	11816.33333	1270.660507
yoqJ	11994	9159	1665.380717
yoqK	18556	13360	1766.57
yoqL	21634.5	15002.33333	1485.51606
yoqM	21955.5	12079	-1638.371867
yoqN	15852	10690.33333	786.3102487
yoqO	25250.5	16295.66667	519.6425121
yoqP	20388	12336	-402.0281762
yoqR	20502	12154.33333	-654.9198385
yoqS	18060.66667	12455	1171.044689
yoqT	20388	12623	-115.0281762
yoqU	17114	10945.33333	252.8370509
yoqW	17502	12147.33333	1212.422153
yoqX	25885.66667	14057.66667	-2115.19734
yoqY	17966.66667	11131.33333	-93.89259526
yoqZ	14277	9639.333333	719.3397944
yorA	17020.33333	13551	2917.02484
yorB	13453	8845.666667	440.4923948
yorC	15300	10204.66667	645.5225086
yorD	19841	12691	294.726847
yorE	17810.66667	11033	-94.76014502
yorF	20819.33333	12016.33333	-991.1835692
yorG	0	0	0
yorH	203.5	0	-127.1428651
yorI	13279.5	9882.333333	1585.558507
yorJ	13516.5	9253.666667	808.8188227
yorK	17642.5	11527.33333	504.64047
yorL	16567	11868.33333	1517.592074
yorM	14254.66667	9432.666667	526.6265626
yorN	12402	9335	1586.470206
yorO	16359.66667	10511.66667	290.463265
yorP	13560.5	9743.666667	1271.328473
yorQ	15782.5	10399.66667	539.0658382
yorR	14320	12025.33333	3078.474226
yorS	0	358.6666667	358.6666667
yorT	22891	10029.33333	-4272.520845
yorV	12017	9158.666667	1650.677428

yorW	14142.66667	11055	2218.93533
yorX	22963	12611.33333	-1735.505052
yorY	0	107.3333333	107.3333333
yorZ	16516	11080.66667	761.7892212
yosA	18142	12302	967.229195
yosB	20797.66667	12718.33333	-275.6466548
yosC	10826.66667	9076	2311.708012
yosD	14916	9741.666667	422.4382835
yosE	14089	10558.33333	1755.798559
yosF	14564.5	10082.33333	982.7153535
yosG	16209	10351.33333	224.2635517
yosH	12608	7601.666667	-275.5679442
yosI	11604.5	8323.666667	1073.399452
yosJ	20325	13536.33333	837.666339
yosK	13620.5	8284.666667	-225.1583664
yosL	15684	10650.33333	851.2734003
yosP	15134	10395.66667	940.2360988
yosQ	13566	10432.33333	1956.558846
yosR	15237.5	10348	827.9046334
yosS	22333	13019.66667	-933.5599009
yosT	18200	11624.66667	253.6585832
yosU	16397	10448.33333	203.8047869
yosV	22909.33333	15672.66667	1359.358176
yosW	15429.33333	9820.333333	180.3842094
yosX	14772.5	11035	1805.427642
yotB	18011.33333	11540.33333	287.2005351
yotC	33513.66667	20254.66667	-684.024244
yotD	15935.5	12299	2342.80773
yotE	13810	9794	1165.779031
yotF	21581	13429.66667	-53.72484159
yotG	15221	9619	109.2135143
yotH	15300.33333	12464	2904.647582
yotI	13509	10503.66667	2063.504678
yotL	18130	12373.66667	1046.39323
yotM	0	1957.666667	1957.666667
yotN	14676.5	10616.66667	1447.073253
youA	7040.5	5972	1573.231736
youB	15611	9461	-292.4509446
yoxA	19212.33333	11092.66667	-910.827709
yoxB	13434.33333	9407	1013.488301
yoxC	18775.66667	10474.33333	-1256.340152
yoxD	30423	16631.66667	-2376.035472
yoyA	5966.666667	2463	-1264.857961
yoyB	15028.33333	10491.66667	1102.254589
yoyC	12403.5	9116.666667	1367.199702
yoyD	12647	9091	1189.398943
yoyE	17303.5	12734.33333	1923.441115

yoyF	15297	10358	800.7301839
yoyG	18543	12219.33333	634.0254821
yoyH	13678.5	10033.33333	1487.271022
yoyI	13053.5	9592.666667	1437.09227
yoyJ	13485	9545	1119.832747
yoyK	14646.6	10440.83333	1289.920861
yozB	12160.66667	8080.666667	482.9172728
yozC	17260.66667	10597	-187.1307799
yozD	13671	9551.666667	1010.29021
yozE	12567	9238	1386.381396
yozF	15045.33333	11053	1652.966651
yozG	16368.33333	11946.66667	1720.048499
yozH	24253.66667	13492.66667	-1660.555296
yozI	21868	12290	-1372.703559
yozJ	15602	11273.66667	1525.838748
yozK	14328.33333	10532.33333	1580.26772
yozL	14777.66667	10037.66667	804.8662753
yozM	0	311.6666667	311.6666667
yozN	19869.66667	12138	-276.183532
yozO	14503.66667	10715.66667	1654.056177
yozP	13158	9431	1210.136024
yozQ	14290.33333	10689	1760.676052
yozS	17247	11152.66667	377.0745559
yozT	19173	13246.33333	1267.413664
yozU	14026	10396.33333	1633.159741
yozV	13761	9414.666667	817.0599503
yozW	16990	11483.66667	868.6431865
yozX	14893	11279.33333	1974.474905
yozY	15528	10512	810.4058505
yozZ	19043.33333	11162.33333	-735.5731101
ypbB	13870	10145.66667	1479.958858
ypbD	12598.5	9317.333333	1446.034139
ypbE	16750.5	10374.33333	-91.05517781
ypbF	15792.5	10573	706.1513649
ypbG	16476.33333	9944.666667	-349.4278124
ypbQ	16235.5	11187.33333	1043.706864
ypbR	16654.66667	9999.333333	-406.1803642
ypbS	14555.5	10054.66667	960.6717128
ypdA	2159.5	0	-1349.213844
ypdP	16120	11020	948.5356975
ypdQ	15667	10085.33333	296.8946716
ypeB	276	0	-172.4394632
ypeP	21584.5	12985.33333	-500.2449072
ypeQ	20888.33333	13862.66667	812.0398983
ypfA	15944	9256	-705.5029057
ypfB	14253	9145.333333	240.3345304
ypfD	15824.33333	10939	1052.262514

ypgQ	17505	11958.66667	1021.881145
ypgR	13680.5	9300.333333	753.0214605
yphA	17084	10155.33333	-518.4195292
yphE	17582	11559	574.1063668
yphF	14679.33333	8842.333333	-329.0302927
yphP	19142	11553	-406.5514689
ypiA	33.5	0	-20.93015224
ypiB	14882	10729.66667	1431.680826
ypiF	16713	11567.66667	1125.70743
ypiP	1266	193	-597.9723205
ypjA	17618	12280	1272.614263
ypjB	107.5	0	-67.16392137
ypjC	15789.5	10817.66667	952.6923736
ypjD	29	1911.666667	1893.548027
ypjG	13527.5	9295	843.2795687
ypjH	15407.66667	10157.33333	530.9211237
ypjP	21768.5	8743.333333	-4857.204549
ypjQ	0	0	0
ypkP	14821	10202.33333	942.4591132
yplP	250	925.6666667	769.4715007
yplQ	0	153	153
ypmA	10620	8409.333333	1774.162682
ypmB	9333.5	5585.333333	-246.0569934
ypmP	416	0	-259.9087562
ypmR	17012.66667	9961	-668.1851752
ypmS	12575	9245.333333	1388.716484
ypmT	13200	9826.666667	1579.561903
ypoC	15555	10663.33333	944.8701059
ypoP	191.3333333	223.6666667	104.1252996
yppC	12394	6946.333333	-797.1982154
yppD	14773	9927.666667	697.7819184
yppE	15182.5	9618.333333	132.6009032
yppF	19961	13004	532.7531673
yppG	12788.5	9237	1246.992479
ypqA	16541	11150.66667	816.1697046
ypqE	11707	9284.666667	1970.359434
ypqP	21345.6	13448.83333	112.5151934
yprA	13291.5	9745.333333	1441.061139
yprB	15767	10758	907.0832718
ypsA	12237	9171	1525.559016
ypsC	17029	12113	1473.610074
yptA	15134	10827.33333	1371.902765
ypuA	19534.33333	11754.33333	-450.3404161
ypuB	15908.66667	10548.66667	609.2393444
ypuC	15085.83333	10658	1232.663034
ypuD	13180.33333	9478.333333	1243.515923
ypuF	18865.33333	10765	-1021.695485

ypuI	19720.66667	13025.33333	704.2421202
ypvA	331.5	0	-207.1147901
ypwA	17653.5	13316.66667	2287.101216
ypzA	17170	11562	834.5160003
ypzC	13413.66667	9740.333333	1359.733768
ypzD	12778	10180.66667	2197.219343
ypzE	21729.5	12683.66667	-892.5047702
ypzF	14223.5	9178.333333	291.7655599
ypzG	307	419.3333333	227.5256695
ypzH	15691	10352	548.5666023
ypzI	19279.5	14134.66667	2089.207856
ypzJ	15728.33333	10717.33333	890.5747908
ypzK	0	0	0
yqaB	12717	9394.666667	1449.330963
yqaC	11176.33333	9012	2029.24304
yqaD	0	463.6666667	463.6666667
yqaM	15953	11591.66667	1624.540735
yqaN	12757	8959.333333	989.0064036
yqaO	13230	9542	1276.151816
yqaP	15805	11688.33333	1813.67494
yqaQ	21963.33333	17626	3903.734018
yqaR	18167.66667	11802	451.193158
yqaS	12016.66667	9288.333333	1780.552355
yqaT	14236.33333	10404.66667	1510.080875
yqbA	0	0	0
yqbB	0	288.3333333	288.3333333
yqbC	14566	10521	1420.444849
yqbD	12513	8943	1125.119552
yqbE	15866.66667	12116.33333	2203.146799
yqbF	11326	8681.333333	1605.067534
yqbG	14212.33333	11576.66667	2697.075611
yqbH	30353.33333	16771	-2193.175752
yqbI	18142	12483.66667	1148.895862
yqbJ	17223.33333	11790.33333	1029.527698
yqbK	14982.66667	10495.33333	1134.452906
yqbM	19500.66667	11913.66667	-269.9728004
yqbN	18984.33333	12734.83333	873.7889491
yqbO	17662.66667	12248.66667	1213.37406
yqbP	18501	12550	990.9329366
yqbQ	21266	14069.33333	782.7477342
yqbR	17546.66667	12170.33333	1207.515284
yqbS	20807.66667	14366.66667	1366.438872
yqbT	24800	16591	1096.439535
yqcA	21132.66667	13829.33333	626.0518227
yqcB	20586	14028.66667	1166.931919
yqcC	20536.66667	14601.33333	1770.421098
yqcD	16459.33333	11426	1142.526792

yqcE	16241	10902.33333	755.2705705
yqcG	16195	11114.33333	996.010481
yqcI	15852.33333	9754.333333	-149.8980115
yqcK	18303.33333	15112.33333	3676.764581
yqdB	25741.66667	14760	-1322.895591
yqeB	15980.33333	10713.66667	729.4633969
yqeC	15935.66667	10070.66667	114.3702665
yqeD	13516.33333	10414.33333	1969.589619
yqeF	30971	16942.66667	-2407.415276
yqeH	6333.333333	4208.666667	251.7224618
yqeI	13645	10141	1615.867841
yqeK	14657.33333	9759.333333	601.7148819
yqeL	25144.33333	20256	4546.306726
yqeM	19618	12487.33333	230.3862684
yqeW	16101.33333	9393.333333	-666.4683968
yqeY	15926.33333	10922.66667	972.2015527
yqeZ	16125	9820	-254.5882059
yqfA	19308	11826.66667	-236.5983925
yqfB	16247.66667	10163.66667	12.43869937
yqfC	15993	9619.666667	-372.4504916
yqfD	22992	12173.33333	-2191.623692
yqfF	14479	9791	744.800767
yqfG	0	0	0
yqfL	0	0	0
yqfO	17486.33333	13082.66667	2157.543717
yqfQ	13747.33333	9769.333333	1180.265286
yqfT	19717.66667	10847	-1472.216871
yqfU	17159	12060	1339.388588
yqfW	14348	9229	264.6470339
yqfX	20593.33333	13682.66667	816.3501942
yqfZ	25049.66667	13462	-2188.547371
yqgA	19646.33333	10687	-1587.649184
yqgB	12629.33333	9889.666667	1999.103402
yqgC	0	0	0
yqgE	17128.66667	10487.33333	-214.3263989
yqgL	13236.33333	9601.333333	1331.528205
yqgM	15701	10984.33333	1174.652129
yqgN	18769.33333	13029	1302.283458
yqgO	13231.66667	10165	1898.110515
yqgQ	13585.66667	9379.333333	891.2714934
yqgS	16913.33333	9894	-673.1236293
yqgT	21618.33333	11635.33333	-1871.38332
yqgU	17501	10634.33333	-299.9530661
yqgV	15422	9974.666667	339.2992676
yqgW	12776.66667	9691	1708.385717
yqgX	19204.66667	13505.66667	1506.962276
yqgY	12589	10045	2179.636222

yqhB	13825.33333	9841.333333	1203.532394
yqhG	14805.33333	12129.33333	2879.247344
yqhH	17604	15041.66667	4043.027859
yqhL	16697.33333	11906.66667	1474.495661
yqhM	17061.66667	10526.66667	-133.1327611
yqhO	19682	12652.66667	355.7336392
yqhP	14596.66667	10474	1354.284909
yqhQ	17598	11509.33333	514.4432095
yqhR	14517.33333	10521.66667	1451.517508
yqhS	18239.66667	12973.66667	1577.875617
yqhT	17636	11699	680.3682109
yqhV	16848.66667	10053.66667	-473.0544797
yqhY	13249.66667	2610	-5668.135537
yqiG	19868	11545.66667	-867.4755643
yqiH	16528	9603	-723.3748134
yqiI	17547	9860.333333	-1102.692977
yqiK	18595.33333	11272.66667	-345.3380393
yqiW	870	0	-543.5591776
yqjA	0	0	0
yqjB	16333.66667	10853.33333	648.3742289
yqjC	14620	10091.66667	957.37336
yqjD	14571	10164	1060.320946
yqjE	14669	10627	1462.092441
yqjF	15362.33333	9530.666667	-67.42215283
yqjL	13670.33333	11211.33333	2670.373397
yqjM	0	36.33333333	36.33333333
yqjN	14859	9988.333333	704.717448
yqjP	18608.66667	13530	1903.664885
yqjQ	14108	10220.66667	1406.26106
yqjT	20450.33333	13554.33333	777.3604958
yqjU	15060.33333	11478.66667	2069.261608
yqjV	13334.33333	9843	1511.966367
yqjX	15705.33333	10113.33333	300.9447461
yqjY	13740.33333	9595.666667	1010.972084
yqjZ	15305.33333	10591	1028.523678
yqkA	17104	11228.33333	542.0848575
yqkB	15659	10659.66667	876.2262502
yqkC	19190	13162.66667	1173.125726
yqkD	18257.66667	13634.33333	2227.296232
yqkE	15467	10442.33333	778.8508043
yqkF	0	0	0
yqkK	13307	9056.666667	742.7103718
yqxA	15417.66667	10463.33333	830.6733171
yqxC	10160.66667	7625.666667	1277.478601
yqxD	13622.66667	7892.333333	-618.8453912
yqxG	17674.66667	12904.33333	1861.543359
yqxH	25757	13661.33333	-2431.142227

yqxI	16791.33333	9991	-499.9003883
yqxJ	18207	12890	1514.618452
yqxK	13605	9819.333333	1319.192401
yqxL	16601	10477.33333	105.3495314
yqxM	23067	10723	-3688.815575
yqzC	8207.333333	7856.333333	2728.550164
yqzD	8204.666667	7833.666667	2707.549579
yqzE	20436.66667	13151	382.5658315
yqzF	0	27.66666667	27.66666667
yqzG	15413	10614.33333	984.5889602
yqzH	15385.66667	10343	730.332965
yqzI	14448.66667	9673.666667	646.4191138
yqzJ	13629	9678	1162.864331
yqzK	14788	10742.33333	1503.076875
yqzL	13856.66667	9960.666667	1303.289267
yqzM	14672	9706.666667	539.8847655
yqzN	18649	12288.66667	637.1320651
yqzO	13210.66667	9386	1132.230909
yraA	19591.66667	11961.66667	-278.8278408
yraB	16742.66667	10754.33333	293.8389374
yraD	19531.66667	12481.66667	278.658999
yraE	15493.33333	10964	1284.064914
yraF	19358.33333	12232	137.2876474
yraG	27571.33333	14824.33333	-2401.702612
yraH	15036.66667	10290.33333	895.7147501
yraI	18921.66667	11659.66667	-162.224796
yraJ	17924	11029.66667	-168.9019536
yraK	16665.33333	10837	424.8219754
yraL	18275	11535.66667	117.8000334
yraM	17309	10477.66667	-336.6618453
yraN	16616	10366	-15.35551185
yraO	19644.66667	13601	1327.392117
yrbC	27743.33333	17751	417.5017804
yrbD	14879.66667	9688	391.471981
yrbE	15130.33333	10249.66667	796.5269612
yrbF	14509.33333	8991	-74.15091317
yrbG	20022	13557.66667	1048.308213
yrdA	0	120.6666667	120.6666667
yrdB	18996.66667	11828.66667	-40.08334577
yrdC	13971.66667	9946.666667	1217.43949
yrdD	17779.44444	11627.55556	519.3024513
yrdF	15232.66667	10473.66667	956.5910733
yrdK	13681.66667	10286.33333	1738.29255
yrdN	13874.33333	9864.666667	1196.251475
yrdP	17186	10646.66667	-90.81382362
yrdQ	28944	16865.33333	-1218.318203
yrdR	14624.66667	8645	-492.2089497

yrhC	14985.33333	9846	483.4534908
yrhD	14451	9652.333333	623.6279589
yrhE	16652.33333	11377.66667	973.6107907
yrhF	15574.66667	10368.66667	637.9160862
yrhG	17011.33333	11627.33333	998.981199
yrhH	21233.66667	13222.66667	-43.71769098
yrhK	19220	11523	-485.2843607
yrhO	15230.66667	10275.33333	759.5073013
yrhP	17976.33333	12496.66667	1265.401192
yrkA	19924.33333	13693.33333	1244.995125
yrkB	18440.33333	12133	611.8362969
yrkC	25100	10588	-5093.994665
yrkD	12850.66667	9840.333333	1811.485281
yrkE	0	996.3333333	996.3333333
yrkF	16948.33333	10635.66667	46.67571418
yrkH	13926	9760.333333	1059.637807
yrkI	18153.66667	10643.33333	-698.7265794
yrkJ	17606.66667	10634.33333	-365.9715562
yrkK	0	0	0
yrkL	13450.33333	9627	1223.49181
yrkN	1201.333333	1269.666667	519.0968291
yrkO	13898.66667	10083	1399.381812
yrkP	23068.33333	15161.33333	748.6847176
yrkQ	15995.33333	10386	392.4250202
yrkS	0	137.3333333	137.3333333
yrpB	18573.66667	13156	1551.532208
yrpC	14607	9129.333333	3.162175322
yrpD	15156.66667	9938.666667	469.0744037
yrpE	15814.33333	9532.333333	-348.1563463
yrpG	26287.66667	14858	-1566.025833
yrrB	12658.33333	9151.333333	1242.651429
yrrC	12291.33333	8978.666667	1299.279266
yrrD	0	0	0
yrrI	29150	16991.66667	-1220.689687
yrrK	6526	7107	3029.681387
yrrL	11844	8769.333333	1369.43115
yrrM	10059.66667	10026.33333	3741.248114
yrrN	14084	10323.66667	1524.255796
yrrO	14461	10126.66667	1091.713486
yrrS	15859	10826.33333	917.9367841
yrrT	14272	10512	1595.130364
yrvC	12164.33333	8943	1342.959744
yrvD	14864.66667	10565.33333	1278.177024
yrvJ	15728.66667	10229	402.0331973
yrvM	27175.33333	15837.66667	-1140.956136
yrvN	12487.66667	9791.333333	1989.280662
yrzA	15429	10916.33333	1276.59247

yrzB	13770	10261.33333	1658.103591
yrzE	12443.33333	9258.666667	1484.312605
yrzF	14457	9734.333333	701.8792749
yrzH	14702.66667	10605.33333	1419.391492
yrzI	13843.33333	10166	1516.953009
yrzK	17938	12935.66667	1728.351117
yrzL	12011	8354.666667	850.4261122
yrzM	17928.33333	10885	-316.2760031
yrzN	18903	12359	548.7711097
yrzO	0	0	0
yrzP	19384.66667	11559	-552.16491
yrzQ	20366.33333	14270.33333	1545.842072
yrzR	22072.66667	14466.33333	675.7579986
yrzS	0	1231	1231
yrzT	14474.66667	9886	842.5081498
ysaA	13816	9754.333333	1122.36368
yscB	21677.33333	11875	-1668.578712
ysdA	18412.66667	11428.33333	-75.54477137
ysdB	12201.66667	8811	1187.634599
ysdC	11880.66667	9497	2074.189192
ysfB	18620	11899	265.5840376
ysfE	21335.66667	13197.66667	-132.4453187
ysgA	17570.33333	12162.66667	1185.062141
yshA	26205.66667	17559.33333	1186.539515
yshB	25809	17524.66667	1399.702511
yshE	26436.66667	14241	-2276.118152
yslB	19199.33333	13434.66667	1439.29444
ysmA	15444.33333	11125.66667	1476.345833
ysmB	14045.66667	10599	1823.539055
ysnA	13643	9864	1340.117402
ysnB	18110	11323.66667	8.888842911
ysnD	14735.66667	10033.66667	827.1070632
ysnE	31531	17877	-1822.959114
ysnF	0	0	0
ysoA	13407.66667	9079.666667	702.8157849
ysxD	4290.333333	4262.333333	1581.816025
ysxE	14433	10136.66667	1119.207344
yszA	0	1108.666667	1108.666667
yszB	16875.66667	11130.33333	586.7431091
ytaB	23775	12552	-2302.160285
ytaF	0	464.6666667	464.6666667
ytaP	14375	10013.66667	1032.444623
ytbD	15703.33333	9425	-386.1390259
ytbE	13550	9567.666667	1101.88867
ytbQ	14944.33333	10032.33333	695.4028314
ytcA	16346.33333	9666	-546.8729928
ytcB	17097.66667	9930.666667	-751.624865

ytcC	17614.33333	11000.33333	-4.761541334
ytcD	15730	10303.66667	475.8668231
ytcI	14997	10567	1197.164383
ytcJ	18014.33333	11848.66667	593.6595264
ytcP	20208	10678	-1947.567657
ytcQ	16696	9549	-882.337965
ytdA	16853.66667	9713	-816.8450496
ytdP	13316.66667	9384.666667	1064.670825
yteA	13533.33333	10227.66667	1772.301681
yteJ	17724.66667	13047	1972.970992
yteP	19700.66667	12314.66667	6.071066822
yteR	14374.33333	8936.666667	-44.13885687
yteS	15535.66667	9038.333333	-668.0508012
yteT	12079.66667	8891.333333	1344.191173
yteU	15261	9071.666667	-463.1110456
yteV	11857	8591	1182.975668
ytfI	18072.33333	11355.33333	64.08891459
ytfJ	15811.66667	10297.33333	418.5097355
ytfP	11255.66667	8539.333333	1507.01044
ytgP	18243	11427.33333	29.45968128
ythA	14872.66667	10124.33333	832.1787789
ythB	12720.33333	9479.666667	1532.248361
ythP	13487.33333	8948.666667	522.041592
ythQ	17077.33333	10368	-301.5876581
ytiB	20858.66667	15377	2344.908391
ytjA	15709.66667	10764.33333	949.2373632
ytjP	19638.66667	13018.66667	748.807468
ytkA	13765.66667	9030.333333	429.8109739
ytkC	18825	10400.33333	-1361.162665
ytkD	13877	10482.66667	1812.585393
ytkK	17121.66667	11211	513.7137324
ytkL	15657	11135.66667	1353.475812
ytkP	16971.66667	11026.33333	422.7641654
ytlA	16717	9630	-814.4583589
ytlC	0	565.3333333	565.3333333
ytlD	0	0	0
ytlI	21684	13801.33333	253.5894167
ytlP	12332	9196	1491.204852
ytlQ	11286.33333	9181	2129.517167
ytlR	10887	9017	2215.012912
ytmA	12149.33333	9513.333333	1922.664787
ytmB	23760.33333	17542	2697.003165
ytmI	17224	12094.33333	1333.111178
ytmO	12582.66667	9605.666667	1744.259833
ytmP	15355.33333	10789	1195.284645
ytnA	12411.66667	9551	1796.43066
ytnI	13104.66667	10196.33333	2008.790993

ytnJ	16726.66667	12072.66667	1622.168761
ytnL	17696	13042.66667	1986.548038
ytnM	16328.66667	11510	1308.164799
ytnP	17937.33333	14059.33333	2852.434304
ytoA	17640.33333	12556.33333	1534.994161
ytoI	14796	10744.66667	1500.411963
ytoP	14772	10799.33333	1570.073366
ytoQ	15214	10449	943.586979
ytpA	12814	9367	1361.060572
ytpB	11494	9493	2311.771049
ytpI	14764.33333	11105.66667	1881.196684
ytpP	13895.66667	10842.33333	2160.589488
ytpQ	16316.66667	14952.33333	4757.9955
ytpR	14322	9880	931.8913312
ytpS	19125	10514.33333	-1434.596864
ytqA	15891.33333	11366.66667	1438.068876
ytqB	18543.66667	13254.33333	1668.608962
ytrA	8434.333333	6148	878.3916202
ytrB	17396.33333	10822.33333	-46.5593566
ytrC	12277.33333	7166.666667	-503.9738046
ytrD	12476.33333	8737.333333	942.3615099
ytrE	13605.33333	8622.333333	121.9841404
ytrF	15175.33333	10274.66667	793.4118313
ytrH	13530.66667	10121.66667	1667.967763
ytrI	14237.33333	10296	1400.789427
ytrP	16955	11268.33333	675.1771764
ytsJ	10506	8588.666667	2024.721011
ytsP	20403.33333	12735.66667	-11.9414797
yttA	12650.33333	9610	1706.316341
yttB	15084	8983.666667	-440.524868
yttP	14813.33333	11235.66667	1980.582432
ytvA	0	0	0
ytvB	12148.33333	9939.333333	2349.289568
ytvI	13550.66667	9970	1503.805483
ytwF	14628	10079.33333	940.0417814
ytwI	13089	9149.333333	971.5792232
ytxB	30265.33333	18185	-724.195054
ytxC	11934	9603	2146.867557
ytxD	15957.66667	11160.33333	1190.291759
ytxE	11444	9292.333333	2142.343415
ytxG	14096	8758	-48.90823874
ytxH	15304.33333	10313	751.148459
ytxJ	22399	13642.66667	-351.7954247
ytxK	16113.33333	11207.66667	1140.367569
ytxM	14853.33333	10011.33333	731.2578718
ytxO	14080	9898.333333	1101.421585
ytzA	17077	10598	-71.37939792

ytzB	14545	8633.666667	-453.7680902
ytzC	18687.33333	12287.33333	611.8488063
ytzD	21513.33333	17123	3681.885317
ytzE	20235	12832.66667	190.2299321
ytzG	17386	12992.66667	2130.230044
ytzH	13775.33333	9617.666667	1011.104761
ytzI	13999.66667	10727.66667	1980.945632
ytzJ	14793.33333	10562.66667	1320.078045
ytzK	13668.66667	10766.66667	2226.748032
ytzL	13362.33333	9887.333333	1538.805842
yuaB	16406	10938.33333	688.1817609
yuaC	21476.66667	14212.33333	794.1272744
yuaD	15846.33333	9938.666667	38.18400583
yuaE	15667.66667	10252.33333	463.4781511
yuaF	15073.33333	10143.33333	725.8061257
yuaG	14929	10427.66667	1100.316135
yuaI	14232.66667	9807.666667	915.3717372
yubA	16897.33333	11144.33333	587.2061947
yubD	17943.33333	11159	-51.6477131
yubF	14921.66667	10163	840.2311931
yueB	22582.33333	15240.66667	1131.661454
yueC	17280.33333	11101.66667	305.2485338
yueD	16118.33333	10735	664.5769986
yueE	16753	11710.66667	1243.716204
yueF	17160	12350.66667	1629.430474
yueG	18851.66667	11915.33333	137.1765172
yueH	27609	15934.66667	-1314.902684
yueI	15574	9952.333333	221.9992733
yufK	13278	9381.333333	1085.495678
yufL	20763	13521.66667	549.3457415
yufM	19388	14872.33333	2759.085821
yufN	15722	9380	-442.8015983
yufO	14892.66667	8849.333333	-455.3168343
yufQ	23435.66667	11248.33333	-3393.818046
yufS	11702	8952.333333	1641.150004
yugE	10940.66667	9153.333333	2317.81635
yugF	30134	16048	-2779.140527
yugG	21793.66667	12936.66667	-679.5948628
yugH	25029	14678.33333	-959.3019041
yugI	22286	12197.66667	-1726.19521
yugJ	20057.66667	13184.33333	652.6910365
yugK	16438	12176.33333	1906.18878
yugM	14646	9405.666667	255.1290628
yugN	25588.66667	13731	-2256.304149
yugO	24087.66667	14274	-775.5083725
yugP	25688.33333	14726.66667	-1322.907289
yugS	14733.33333	10487	1281.898218

yugT	20297.33333	13188	506.618604
yugU	31089.66667	17725.33333	-1698.889248
yuiA	32067	19048.66667	-986.1748835
yuiB	19211	12835.66667	833.0053319
yuiC	17956.66667	11924.33333	705.3552114
yuiD	17440.33333	12443.33333	1546.950294
yuiF	12883.33333	10468.66667	2419.409113
yuiH	12950.33333	9734.666667	1643.548809
yukB	15839.33333	8488.666667	-1407.44253
yukC	35242.66667	20643	-1375.936679
yukD	11541.33333	9285.333333	2074.531431
yukE	11596	9019	1774.043421
yukF	17082.66667	11697.33333	1024.413512
yukJ	14925.33333	11094.66667	1769.606997
yulB	12548.66667	9759	1918.835709
yulC	19471.66667	14234.33333	2068.812506
yulD	20668	16321	3408.033238
yulE	17771.66667	11351	247.6063009
yulF	16051	10152	123.6455633
yumB	18877.66667	13720.66667	1926.265553
yunB	16222.33333	11239.66667	1104.266476
yunC	15452.33333	10451	796.6809207
yunD	17514.66667	11634.66667	691.8415982
yunE	21152.66667	11757.33333	-1458.443791
yunF	19804.33333	13146.33333	772.9688047
yunG	15440.33333	12083.33333	2436.511622
yurJ	14010.66667	10596.33333	1842.739711
yurQ	10688.66667	9529.333333	2851.261077
yurR	14980.66667	10580.66667	1221.035801
yurT	12503.66667	8985.333333	1173.284172
yurZ	13392	8267.333333	-99.729318
yusD	16758.66667	12253.33333	1782.842447
yusE	13365.66667	9712	1361.389906
yusF	14195.33333	9796.333333	927.3635486
yusG	16467	11864	1575.736807
yusH	23106.33333	16042	1605.609719
yusI	13371.66667	9514	1159.641222
yusN	0	0	0
yusO	13627.66667	9222	707.6973722
yusP	15525	9749.666667	49.94685917
yusQ	13653	9295.333333	765.2029287
yusR	22096.33333	17514.33333	3708.971523
yusS	13502.66667	10728.33333	2292.128289
yusT	12248.66667	8358	705.2699078
yusU	13786.33333	10197.66667	1584.232174
yusV	16799.33333	4160.333333	-6335.5653
yusW	16637.33333	9609.333333	-785.3508327

yusZ	18185.66667	11293.66667	-68.38622733
yutD	12318.33333	9017	1320.743522
yutE	11544.66667	8811	1598.115495
yutF	14343.33333	10164.66667	1203.229344
yutG	11523.66667	9403	2203.235889
yutH	23877.33333	14617.66667	-300.4295062
yutI	12952	9448.333333	1356.174174
yutJ	18800	13389.33333	1643.456851
yutK	17806.66667	9865	-1260.261022
yutM	14926.33333	10558.33333	1232.648883
yuxG	11360	9527.666667	2430.158324
yuxH	17177.66667	11703.33333	971.0593486
yuxJ	13400.33333	9430	1057.730843
yuxK	11133.33333	5171.666667	-1784.224725
yuxL	14872	11171.33333	1879.595299
yuxN	14302.66667	10300.66667	1364.637091
yuxO	22513.66667	12696	-1370.103607
yuzA	20894.66667	12537	-517.5837126
yuzB	30894.66667	14972	-4330.390352
yuzC	12105.33333	9456.666667	1893.48847
yuzD	12011	9244.666667	1740.426112
yuzE	12650.66667	9937.333333	2033.441414
yuzF	15034.33333	10466.66667	1073.505905
yuzG	14519.66667	10780.66667	1709.059687
yuzH	17783.33333	11625	514.3171931
yuzI	27948.33333	14800	-2661.578256
yuzJ	15597.66667	10314	568.8794643
yuzK	10769	7534	805.7370301
yuzL	15186	10865.66667	1377.747504
yuzM	13313	9272.666667	954.9616878
yuzN	12854.66667	8924	892.6528254
yuzO	15508.66667	10260.33333	570.8182767
yvaA	26540.33333	14795.33333	-1786.553748
yvaB	13010	9974.333333	1845.936896
yvaC	21163.33333	13230	7.558549048
yvaE	21755.33333	12029.33333	-1562.978271
yvaF	15484.33333	11011	1336.68794
yvaG	13982	9640.333333	904.6500903
yvaK	13616.33333	8597.666667	90.44488639
yvaM	16419	10639.66667	381.3929456
yvaP	14088.66667	9753	950.6734861
yvaQ	14939.66667	10128.33333	794.3184745
yvaV	15283.66667	10862	1313.060593
yvbF	28392.66667	15432	-2307.189131
yvbG	11919	8196.333333	749.5726
yvbH	7877.666667	4281	-640.8138102
yvbI	15533.66667	10746.33333	1041.19876

yvbJ	12376.66667	9391.666667	1658.964649
yvbK	17308.5	10985.66667	171.6505451
yvbT	15223.33333	11029	1517.755693
yvbU	16894	13156.33333	2601.288797
yvbW	19902.66667	12957	522.198706
yvbX	208.3333333	243	112.8373617
yvbY	329.3333333	247	41.23890135
yvcA	655.5	1060.333333	650.7896081
yvcB	16895.33333	11236	680.1224227
yvcD	19607	12220	-30.07447766
yvcI	15844.66667	9626.666667	-272.7746931
yvcJ	12810.33333	9258.333333	1254.684768
yvcK	0	0	0
yvcN	17497.33333	11568.33333	636.3377963
yvcP	14762	11370	2146.987839
yvcQ	12852.5	9869.333333	1839.33985
yvcR	12400.5	9009.333333	1261.74071
yvcS	14276	10186	1266.631242
yvcT	15884.66667	10203	278.5674137
yvdA	21469	12332.66667	-1080.749407
yvdB	13669	9426.666667	886.5397714
yvdC	17420.33333	11420	536.1125741
yvdD	18658	8494.333333	-3162.824294
yvdJ	10013	8152.666667	1896.737879
yvdP	13765.33333	9555.333333	955.0192341
yvdQ	1012.666667	0	-632.6945523
yvdS	13437.66667	9484	1088.405698
yvdT	12534.66667	9553	1721.582638
yveA	15699	11205	1396.568357
yveF	16484.33333	10195.66667	-103.4260578
yveG	14445.33333	9556	530.8350493
yvfG	15915	11638.33333	1694.949067
yvfH	27424	15875	-1258.984928
yvfI	25601.33333	17009.33333	1014.115296
yvfR	0	122.3333333	122.3333333
yvfS	20466	12296.33333	-490.4277346
yvfT	18764	12843.66667	1120.282289
yvfU	26932.66667	15446.66667	-1380.342695
yvfV	21651.66667	12832.66667	-694.8760085
yvfW	24305.66667	14275.33333	-910.3772239
yvgJ	25660	11596.33333	-4435.538503
yvgK	15758.33333	11127.66667	1282.164704
yvgL	22030	11757.33333	-2006.584693
yvgM	22442	11572.66667	-2448.660993
yvgN	16074.33333	10224.33333	181.4006811
yvgO	30851.66667	17271	-2004.524783
yvgT	15289.66667	10638.66667	1085.978575

yvhJ	188	0	-117.4587648
yviA	15401	10654	1031.752995
yviE	18883.33333	13681.33333	1883.391796
yvjA	15530	10175.33333	472.4896225
yvkA	18587.5	13378	1764.889409
yvkB	18246.33333	12026.66667	626.7104124
yvkC	0	337.3333333	337.3333333
yvkN	15436	9671	26.88567159
yvlA	14087.33333	9093	291.506527
yvlB	17541.33333	10758	-201.4858862
yvlC	17644.33333	11087.66667	63.82837208
yvlD	13288.66667	9693.666667	1391.164684
yvmA	14129	9331.666667	504.140666
yvmB	17826.66667	11418.33333	280.5766977
yvmC	20943	13926.33333	841.5518887
yvnA	15209	11031	1528.710882
yvnB	14964.33333	9988.333333	638.9072181
yvoD	17958.33333	11055	-165.0194231
yvoF	21753.33333	14337.66667	746.604624
yvpB	16384	11017.33333	780.9269355
yvqJ	14277	8435	-484.9935389
yvqK	11543.33333	9680.666667	2468.615203
yvrA	13099.66667	9954.666667	1770.248229
yvrB	13670.66667	10630.66667	2089.49847
yvrC	21497	11340	-2090.909932
yvrD	14516.66667	10274.33333	1204.600695
yvrE	16752.33333	10890.33333	423.799391
yvrH	0	0	0
yvrJ	12600	9712.333333	1840.096968
yvrL	10543.66667	6092.666667	-494.8123936
yvrN	26554.33333	11961.66667	-4628.967344
yvsG	15870.33333	11231	1315.522603
yvyC	18848.33333	15192	3415.925786
yvyD	15270.33333	10483.33333	942.7243349
yvyE	16468	12331.66667	2042.778693
yvyF	17894.33333	11744	563.9665394
yvyG	11552.66667	10196	2978.11725
yvyI	15456	10054.33333	397.7233916
yvzA	12762	9140.666667	1167.215834
yvzB	16057	10133	100.8968793
yvzC	23424	13248.33333	-1386.528939
yvzE	10814.66667	6567.666667	-189.1279535
yvzF	15332	11245.66667	1666.529527
yvzH	15894.5	10724.66667	794.0904038
yvzI	9620	8178.666667	2168.27668
yvzJ	19056.5	12761.33333	855.2006112
ywaC	18594.66667	12908.33333	1290.745148

ywaD	15343.66667	9897	310.5737529
ywaE	12521.66667	10253	2429.704787
ywaF	27517.33333	17201	8.702210443
ywbA	16332	10693	489.0821967
ywbB	15698.66667	10756.66667	948.4432839
ywbC	15165.33333	10063.33333	588.3263046
ywbD	15276.66667	12031.66667	2487.100724
ywbE	26433.66667	15444	-1071.24381
ywbF	18850.33333	12445	667.6762247
ywbI	17128.66667	12002.66667	1301.006934
ywbO	12512.66667	9151.333333	1333.661146
ywcA	18032	11499.66667	233.6217347
ywcB	19277.66667	11806.33333	-237.9800457
ywcC	25077.66667	15236.33333	-431.7078965
ywcE	14463	9803	766.7972576
ywcH	15308	10963	1398.857597
ywcI	16573.66667	10882	527.0935363
ywcJ	21002	12075.33333	-1046.310171
ywdA	16164.33333	10690.33333	591.1704214
ywdD	18666.66667	10728.33333	-934.23906
ywdE	14976.33333	10811.66667	1454.743183
ywdF	14026.33333	10248.33333	1484.951481
ywdH	22178	13136	-720.3855646
ywdI	15436	10443.33333	799.2190049
ywdJ	17385	11071.66667	209.8548243
ywdK	146	468	376.7820231
ywdL	18195	12118.66667	750.7824865
yweA	17433.5	12178	1285.886295
ywfA	23397	10783.66667	-3834.326527
ywfH	18518.66667	14426.33333	2856.228478
ywfL	553	0	-345.5037072
ywfM	15768.5	10025.66667	173.8127675
ywfO	15697.66667	10631	823.4013979
ywgA	11964.66667	11216.66667	3741.374283
ywgB	25470	14227	-1686.16351
ywhA	19431	12526.66667	386.5535859
ywhB	17165.33333	10485.66667	-238.9016899
ywhC	14357.66667	9824	853.6074875
ywhH	12246.66667	9637.333333	1985.852802
ywhK	15382.66667	10388	777.207307
ywhL	21565.66667	13296.66667	-177.1448714
ywiC	11931.66667	9214.333333	1759.658712
ywiE	13239	9539.333333	1267.862124
ywjA	14475.66667	9721.333333	677.2167025
ywjC	17404.33333	12182.33333	1308.442398
ywjD	14302.66667	9831	894.970424
ywjG	22105.66667	13595.66667	-215.5264299

ywjH	20635.66667	11664.33333	-1228.432187
ywjI	15378.66667	10416.66667	808.3730963
ywkB	12487	11020.66667	3219.030516
ywkD	22143	14507.33333	672.815092
ywkF	24329	17000.33333	1800.044561
ywlA	30083	16874.33333	-1920.94338
ywlB	16736.66667	11204	747.254288
ywlC	12239.66667	12122.66667	4475.5596
ywlD	15160	10445.66667	973.9918015
ywlE	16979.33333	10682	73.64084693
ywlF	8427.333333	5742	476.7650848
ywlG	13557	9888	1417.848539
ywmA	15095	10569.33333	1138.269211
ywmB	15063.33333	10243.33333	832.0539323
ywmC	17464.33333	11367.33333	455.9555583
ywmD	16285.33333	10470.66667	295.9052944
ywmE	19768.33333	10183.33333	-2167.539091
ywmF	16121	10535.66667	463.5775835
ywnA	14037.33333	9779.333333	1009.078894
ywnB	15694.66667	10199.66667	393.9424065
ywnC	16790.33333	10811	320.7243924
ywnF	12696.66667	9341	1408.36817
ywnG	21185.33333	13120	-116.1866256
ywnJ	17741.66667	11503.66667	419.0163875
ywoB	16448.33333	10663.66667	387.0660461
ywoD	16864	10031.66667	-504.6344498
ywoF	14118	10021.66667	1201.013253
ywoG	15564.5	12158.66667	2434.268023
ywoH	22667.66667	15740.33333	1578.013504
ywpD	13918	11942.33333	3246.636053
ywpE	12298	9854	2170.447395
ywpF	16507.33333	10448.33333	134.8706536
ywpG	21472.66667	11745	-1670.706936
ywpJ	16483	11372.66667	1074.406983
ywqA	19542.33333	13630.66667	1420.994672
ywqC	0	1805.333333	1805.333333
ywqE	12635.33333	9706.333333	1812.021384
ywqG	15166.66667	10362	886.1599304
ywqH	14734.66667	9964.333333	758.3985106
ywqI	14428.33333	10018.33333	1003.789654
ywqJ	13841.66667	9739.333333	1091.327643
ywqL	19769.33333	12318.66667	-32.83053877
ywqM	17691.66667	11574.66667	521.2554207
ywqN	19364.66667	10589.66667	-1509.00263
ywqO	17863.66667	10567	-593.8735202
ywrA	117	0	-73.09933768
ywrD	17985.66667	11592.33333	355.2365721

ywrE	13771.66667	9596.666667	992.3956233
ywrF	13511.33333	9161.333333	719.7135228
ywrJ	14319	10687.33333	1741.099007
ywrK	13738	10093.33333	1510.096572
ywrO	15452.33333	9705	50.68092075
ywsA	16358	9984.333333	-235.8287672
ywsB	0	0	0
ywtE	12398.33333	9231.333333	1485.094402
ywtF	18411.66667	12197.66667	694.4133426
ywtG	13578	9551	1067.728145
ywzA	15542.33333	10462.66667	752.1173277
ywzB	15495.33333	10407.33333	726.1486855
ywzC	16651	12077	1673.777165
ywzD	15689	10374.33333	572.149497
ywzE	16774.66667	10418.33333	-62.15404386
ywzF	17205.66667	9324.333333	-1425.43451
ywzH	13802.33333	8671.666667	48.2356829
yxaB	14446	9701.333333	675.7518622
yxaC	14450	9724	695.9194062
yxaD	14498	10299.66667	1241.596601
yxaF	21395	13149	-218.1823048
yxaH	15409	10420.33333	793.0880829
yxaI	26591.33333	17482.66667	868.9157719
yxaJ	23875.33333	14526.33333	-390.5132782
yxaL	24944.66667	16715	1130.054599
yxaM	13461	9968.666667	1558.49415
yxbB	19767.66667	15208.66667	2858.210762
yxbC	5777	2170.666667	-1438.691229
yxbD	28770.66667	18309	333.6437783
yxbF	14319.33333	10571.66667	1625.22408
yxbG	16088	11293.33333	1241.862012
yxcA	21784.66667	14711.66667	1101.028163
yxcD	15835	10351.33333	457.93152
yxcE	16556.33333	10468	123.9230678
yxdJ	14379	11044	2060.278833
yxdK	23254.33333	14046.66667	-482.1911526
yxdL	29223.66667	13556.66667	-4701.715196
yxdM	17330.33333	10028.33333	-799.3238328
yxeA	17297.66667	11523	715.7523356
yxeB	15246.33333	10433.66667	908.0524042
yxeC	13768	9513	911.019819
yxeD	15488.33333	10116.33333	439.5221502
yxeE	15093.66667	10586	1155.768919
yxeF	15321.33333	8823	-749.4728123
yxeG	15740.66667	10686.33333	851.8691626
yxeH	20042.33333	13132	609.9376733
yxeJ	13404	8294.666667	-79.89335264

yxeK	13986.33333	9939.666667	1201.276041
yxeL	22528.33333	13438	-637.2670572
yxeM	18559.33333	9365	-2230.512602
yxeN	18067.33333	9156	-2132.120515
yxeO	16447.33333	8844.666667	-1431.309173
yxeP	16315.33333	10488.33333	294.8285411
yxeQ	14409.66667	10265.33333	1262.452226
yxeR	23217	12332.33333	-2173.199341
yxiB	0	0	0
yxiC	16212.66667	10770.66667	641.3060226
yxiE	26561	16775.66667	180.867452
yxiF	15879.66667	11109.33333	1188.02465
yxiG	14999.33333	10274.33333	903.0398948
yxiH	15019.33333	10065.66667	681.8776149
yxiI	15726	10544.33333	719.0326124
yxiJ	18593.66667	13331.66667	1714.703262
yxiK	25538	14654	-1301.648595
yxiM	27162.33333	13729.33333	-3241.167321
yxiO	16250.33333	7675.333333	-2477.560716
yxiP	15131.33333	9761	307.2355139
yxiS	14952.33333	9797.666667	455.7379194
yxiT	15303.83333	10022.66667	461.127516
yxjA	19983	11211	-1273.992007
yxjB	17643.33333	11460.66667	437.4531527
yxjC	13430	9388.666667	997.8623501
yxjG	27933.66667	16640	-812.414806
yxjH	23323.33333	14915.66667	343.6989816
yxjI	15266	9941.666667	403.7650511
yxjJ	15671	9746	-44.93778443
yxjL	17049	10457	-194.8855393
yxjM	22945.66667	12760	-1576.008854
yxjN	16099	10110.66667	52.32275807
yxjO	16794	10665.33333	172.7668633
yxkA	15919.33333	10871.66667	925.5750174
yxkC	16846.33333	14576.33333	4051.070009
yxkD	21792	11425.66667	-2189.553562
yxkF	15854.66667	10652.33333	746.644167
yxkH	15294	8802	-753.3954741
yxkI	14286.33333	9469	543.1751749
yxkO	12951.33333	9088.666667	996.9240279
yxlA	18961.66667	12471	624.1173108
yxlC	17204.33333	9201.666667	-1547.268136
yxlE	28207.66667	14470.66667	-3152.938041
yxlF	15175	9367	-114.0465751
yxlH	21246	11235.66667	-2038.423319
yxnA	19664.66667	13242	955.8965041
yxnB	22798.66667	16220	1975.833903

yxxB	18838	11937.66667	168.0485196
yxxE	18532.33333	11583	4.356475845
yxxF	17832.66667	10896	-245.5053196
yxxG	19916.33333	12612.33333	168.9933703
yxzC	20754	12473	-493.6978992
yxzE	14667.33333	9605.666667	441.8004086
yxzF	13262	9988.333333	1702.492168
yxzG	15872.66667	9889.666667	-27.26855166
yxzI	16874	10893.66667	351.1177435
yxzJ	22497.33333	9906.333333	-4149.565523
yxzK	12930	9578	1499.586015
yxzL	14207.66667	9888.333333	1011.65792
yyaD	16644	12662.33333	2263.483963
yyaE	12838.66667	8249	227.649316
yyaJ	15344.33333	11324.33333	1737.490566
yyaM	14548.33333	10389	1299.482641
yyaO	7551.666667	3807	-911.1353138
yyaQ	14889.33333	10768.33333	1465.765768
yybF	21506.66667	14310.33333	873.3838544
yybJ	16220.33333	11580	1445.849371
yycA	15545.66667	10296	583.3680588
yycB	10874.33333	6183.333333	-610.7398665
yycI	13634	9517.333333	999.0737613
yycJ	17641	12626	1604.244308
yycN	14447.33333	10637.33333	1610.918821
yycO	13489.33333	9956.666667	1528.792031
yycP	14296	10273	1341.135628
yycQ	15158.66667	11087	1616.158176
yycR	15777.33333	9021.333333	-836.0394617
yycS	14964	9806	456.782145
yydD	15502.33333	11136.66667	1451.108554
yydF	13479.66667	10209	1787.16491
yydG	13214.66667	9502.666667	1246.398453
yydI	12665.66667	8806	892.7363709
yydK	2122.666667	1350.333333	24.13224403
yyzE	10175	7606	1248.856745
yyzG	17489	12131.33333	1204.544302
yyzN	9822.666667	4609.666667	-1527.345535
yyzO	789	0	-492.9519438
zosA	19024	9753	-2132.827351
zur	14924	9030.333333	-293.8932951
zwf	18374	10531	-948.719919
