# Supplementary material for: Mirubactin C rescues the lethal effect of cell wall biosynthesis mutations in Bacillus subtilis
Source: Front Microbiol. 2022 Oct 13;13:1004737. doi: 10.3389/fmicb.2022.1004737 (PMC9609785; doi:10.3389/fmicb.2022.1004737)
Supplement: Supplementary file 2 [file Data_Sheet_2.pdf]

Supplementary Information

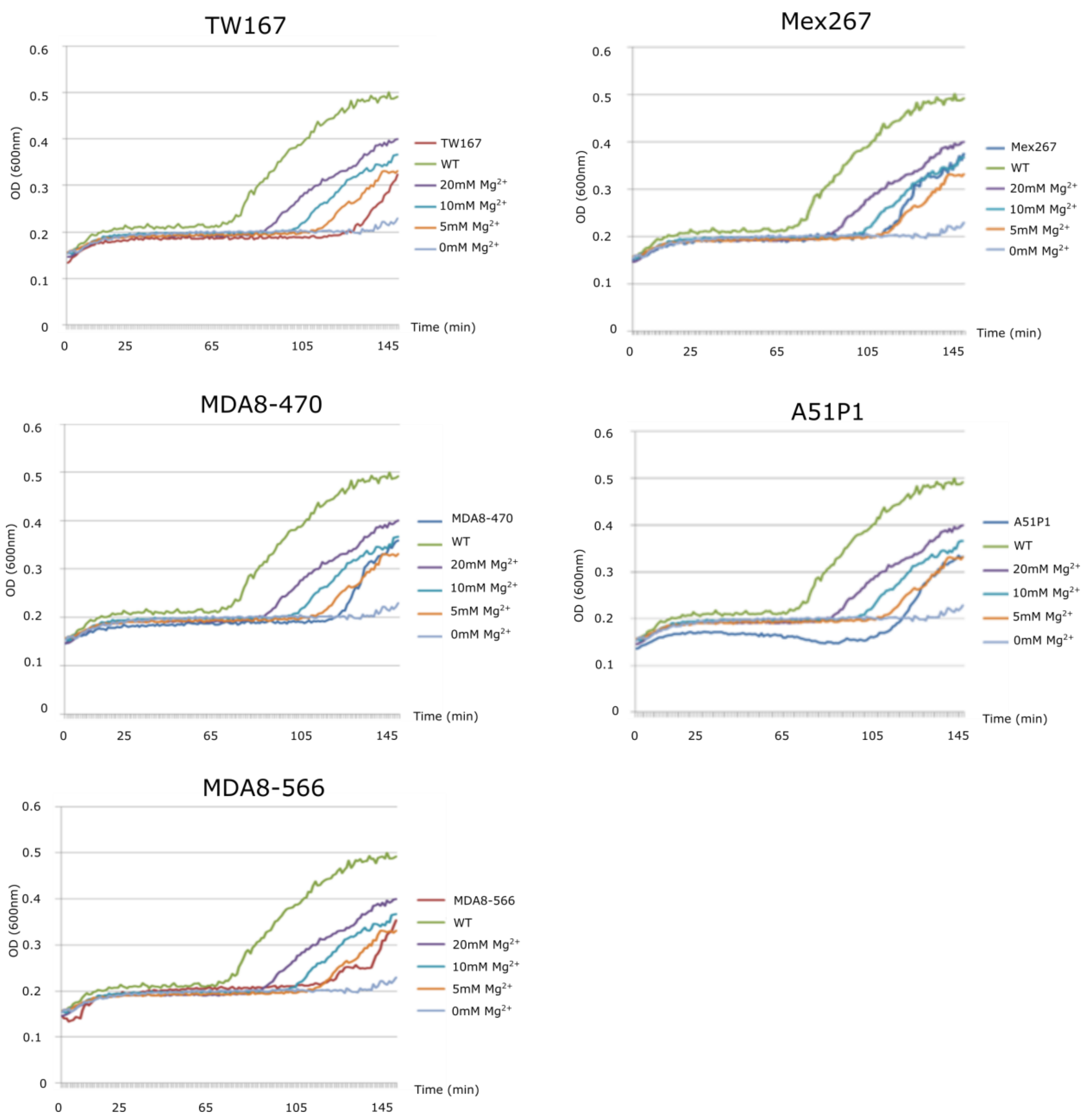

Supplementary Figure 1 Confirmation of the *mbl*-rescuing activity of hits from primary extract screening. The *mbl* mutant and wild type control strains were cultured in NB (96 well plates) with added Mg^{2+} as indicated (mM). Fresh agar crush extracts from the indicated strains were added to a concentration of 6% (vol/vol). Growth was recorded as OD_{600}.

Supplementary Table 1 antiSMASH output of the prioritised strains

MD8A-566						
Region	Type	From	To	Most similar known cluster		Similarity
Region 1	lassopeptide	49,959	72,289	kanamycin	Saccharide	1%
Region 2	lanthipeptide-class-iv	159,332	181,995	venezuelin	RiPP:Lanthipeptide	100%
Region 3	NRPS,T3PKS	211,829	268,782	violapyrone B	Polyketide	28%
Region 4	NRPS	414,129	477,202	atratumycin	NRP	7%
Region 5	terpene	727,150	752,475	hopene	Terpene	76%
Region 6	RiPP-like	1,268,156	1,279,454			
Region 7	NRPS	1,493,212	1,580,665	friulimicin A / friulimicin B / friulimicin C / friulimicin D	NRP	21%
Region 8	siderophore	1,708,307	1,722,357	ficellomycin	NRP	3%
Region 9	terpene	2,087,836	2,108,076	BD-12	NRP	17%
Region 10	LAP,thiopeptide	4,436,710	4,471,437			
Region 11	butyrolactone	4,984,835	4,994,482			
Region 12	ectoine	5,453,618	5,463,115	ectoine	Other	100%
Region 13	terpene	5,863,622	5,882,692	steffimycin D	Polyketide:Type II + Saccharide:Hybrid/tailoring	19%
Region 14	blactam	6,137,403	6,159,229	tabtoxin	Other	13%
Region 15	NRPS,betalactone	6,319,170	6,389,723	coelichelin	NRP	100%
Region 16	T2PKS,terpene,NRPS	6,476,466	6,602,937	spore pigment	Polyketide	83%
Region 17	T2PKS,terpene	6,620,932	6,691,617	lugdunomycin	Polyketide	55%
Region 18	T1PKS,PKS-like,NRPS	6,940,306	7,056,880	salinomycin	Polyketide:Modular type I	32%
Region 19	T1PKS,butyrolactone,oligosaccharide	7,056,948	7,120,066	lidamycin	NRP + Polyketide	39%
Region 20	ectoine,T2PKS,butyrolactone	7,148,573	7,229,947	kosinostatin	NRP + Polyketide	22%

TW167						
Region	Type	From	To	Most similar known cluster		Similarity
Region 1.1	T2PKS,butyrolactone,ectoine	1,882	83,259	kosinostatin	NRP + Polyketide	22%
Region 1.2	oligosaccharide,T1PKS,butyrolactone,PKS-like,NRPS	111,705	291,764	C-1027	Polyketide	42%
tig00000012						
Region 3.1	T2PKS,terpene	252,049	322,751	lugdunomycin	Polyketide	55%
Region 3.2	NRPS,T2PKS,terpene	343,158	467,350	spore pigment	Polyketide	83%
Region 3.3	NRPS,betalactone	553,868	624,767	coelichelin		
Region 3.4	blactam	784,515	806,353	tabtoxin	Other	13%
Region 3.5	terpene	1,060,336	1,081,358	steffimycin D	Polyketide:Type II + Saccharide:Hybrid/tailoring	19%
Region 3.6	ectoine	1,480,132	1,490,530	ectoine	Other	100%
Region 3.7	thiopeptide,LAP	2,471,932	2,506,665			

tig00000014						
**Region 5.1**	terpene	1,176,059	1,196,304	BD-12	NRP	17%
**Region 5.2**	siderophore	1,561,489	1,575,905	ficellomycin	NRP	3%
**Region 5.3**	NRPS	1,703,576	1,791,102	friulimicin A / friulimicin B / friulimicin C / friulimicin D	NRP	24%
**Region 5.4**	RiPP-like	2,004,590	2,015,888			
**Region 5.5**	terpene	2,531,096	2,557,711	hopene	Terpene	76%
**Region 5.6**	NRPS	2,817,761	2,870,070	cadaside A / cadaside B	NRP	14%
**Region 5.7**	T3PKS,NRPS,NRPS-like	3,015,453	3,072,699	violapyrone B	Polyketide	28%
**Region 5.8**	lanthipeptide-class-iv	3,102,253	3,124,916	venezuelin	RiPP:Lanthipeptide	100%
**Region 5.9**	lassopeptide	3,211,737	3,234,185			

Mex267						
Region	Type	From	To	Most similar known cluster		Similarity
**Region 1.1**	NRPS,terpene	507,421	618,704	nocobactin NA	NRP + Polyketide	87%
**Region 1.2**	NAPAA	968,144	1,002,052	isatropolone A / isotropolone B / isotropolone C	Polyketide	9%
**Region 1.3**	T1PKS	1,204,735	1,249,486	clorobiocin	Saccharide:Hybrid/tailoring + Other:Aminocoumarin	7%
**Region 1.4**	RiPP-like	1,543,949	1,553,780			
**Region 1.5**	ranthipeptide	2,000,320	2,021,825	2'-chloropentostatin / 2'-amino-2'-deoxyadenosine	Other	6%
**Region 1.6**	NRPS,RiPP-like	2,050,975	2,168,960	Sch-47554 / Sch-47555		
**Region 1.7**	NRPS,T1PKS	3,843,816	3,925,042	pepticinnamin E	NRP + Polyketide	6%
**Region 1.8**	NRPS-like	4,109,167	4,152,023	platensimycin / platencin	Terpene	5%
**Region 1.9**	NRPS	4,348,301	4,390,052	diisonitrile antibiotic SF2768	NRP	22%
**Region 1.10**	T1PKS	4,441,229	4,487,630			
**Region 1.11**	ectoine	4,508,666	4,519,061	ectoine		
**Region 1.12**	NRPS	4,595,594	4,651,731			
**Region 1.13**	NRPS	4,955,436	5,019,662	herboxidiene	Polyketide	5%
**Region 1.14**	NRPS	5,111,873	5,170,064	pentalenolactone	Terpene	15%
tig00000002						
**Region 2.1**	betalactone	284,078	315,945			
**Region 2.2**	NRPS	342,406	387,295	heterobactin A / heterobactin S2	NRP	63%
**Region 2.3**	terpene	645,041	664,258	carotenoid	Terpene	27%
**Region 2.4**	T3PKS	739,330	780,499			
**Region 2.5**	arylpolyene	803784	844956	echoside A / echoside B / echoside C / echoside D / echoside E		11%
**Region 2.6**	terpene	1,244,472	1,263,989			
**Region 2.7**	terpene	1,277,197	1,303,501	A54145	NRP	5%
**Region 2.8**	NRPS-like,NRPS,	1,363,396	1,437,883			

	betalactone					
Region 2.9	terpene	1,900,050	1,918,393	carotenoid	Terpene	18%
Region 2.10	terpene	1,949,854	1,970,960			
Region 2.11	betalactone	2,184,602	2,210,492			
tig00000006						
Region 6.1	NAPAA	101,882	135,916			
tig00000007						
Region 7.1	terpene	133,600	154,517	lavendiol	Polyketide	6%
tig00000008						
Region 8.1	terpene	1	13,490	carotenoid	Terpene	50%
tig00000009						
Region 9.1	NAGGN	3,031	18,158	bottromycin A2	RiPP:Bottromycin	6%

Supplementary Table 2 BlastP analysis of the mirubactin gene cluster identified in strain MD8A-470 against the mirubactin gene cluster (BC0000392 MIBIG accession number; NCBI GenBank: CP001630.1)

ID mirubactin cluster	Max Score	Total Score	Query Cover	E value	Per. Ident	Acc. Len
mrbA	244	244	96%	8.00E-86	55.89%	279
mrbB	296	342	72%	5.00E-99	57.39%	399
mrbC	588	588	98%	0	57.19%	553
mrbD	210	210	92%	7.00E-74	54.50%	211
mrbE	407	407	89%	7.00E-147	73.74%	297
mrbF	426	426	97%	2.00E-153	69.67%	334
mrbG	348	348	91%	1.00E-122	71.15%	342
mrbH	444	444	88%	3.00E-160	78.07%	332
mrbI	417	417	99%	6.00E-152	77.31%	262
mrbJ	3705	3705	99%	0	62.76%	3328
mrbK	566	566	97%	0	68.05%	467
mrbL			no significant hit			
mrbM			no significant hit			
mrbN			no significant hit			
mrbO	30.4	30.4	28%	8.00E-04	42.86%	203

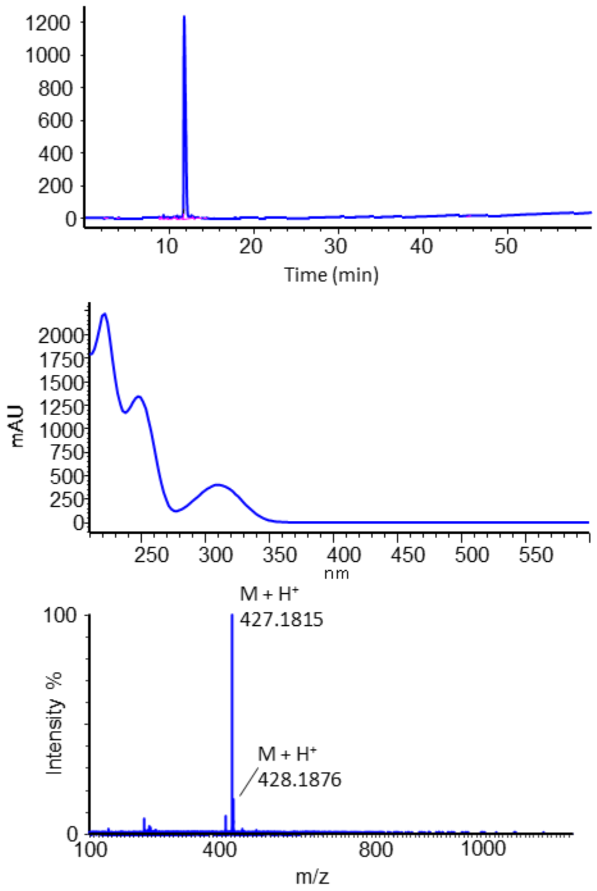

Supplementary Figure 2 (A) HPLC trace of purified mirubactin C. (B) UV-Vis spectrum of mirubactin C. (C) HRMS spectrum showing molecular ion $[M+H]^+$ = 427.1815.

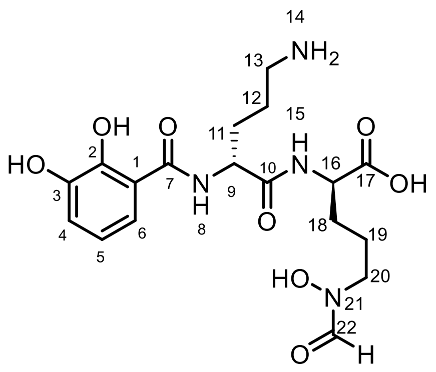

Supplementary Table 3 NMR data for mirubactin C in D_2O at 298 K, 700 MHz, ^1H NMR collected at 700 MHz, $^{13}C\{^1H\}$ NMR collected at 176 MHz, ^{15}N shifts were measured indirectly by ^1H-^{15}N HMBC performed at both 8 and 12 Hz, referencing against nitromethane

Position	$\delta_{^1H}$ multi, (*J* in Hz)	$\delta_{^{13}C}$	$\delta_{^{15}N}$	HMBC
1	-	116.4		
2	-	146.3 or		
3	-	144.4		
4	6.98 (dd, *J* = 7.9, 1.5 Hz, 1H)	119.5		H to C1, 2, 3, 5, 6
5	6.76 (t, *J* = 8.0 Hz, 1H)	119.6		H to C1, 2, 3, 4, 6, 7
6	7.18 (dd, *J* = 8.1, 1.6 Hz, 1H)	119.2		H to C1, 2, 3, 4, 5, 7
7	-	169.8		
8	-	-	-260.5	
9	4.53 (dd, *J* = 7.8, 6.4 Hz, 1H)	53.2		H to C1, 7, 10, 11, 12
10	-	172.6		-
11	1.98 – 1.91 (m, 1H)<br>1.89 – 1.82 (m, 1H)	27.8		H to C9, 10, 12, 13
12	1.80 – 1.71 (m, 2H) [overlap C18]	23.1		*
13	2.99 (ddd, *J* = 8.6, 6.9, 2.1 Hz, 2H)	38.8		H to C11, 12
14	-	-	-348.8	
15	-	-	-252.9	
16	4.21 – 4.13 (m, 1H)	54.6		H to C10, 17 18, 19
17	-	178.1		
18	1.80 – 1.71 (m, 1H) [overlap C12]<br>1.69 – 1.60 (m, 1H) [overlap C19]	28.2		*
19	1.69 – 1.60 (m, 2H) [overlap C18]	22.7		*
20	3.56 – 3.44 (m, 2H)	50.0		H to C16, 18, 19, 22
21	-	-	-199.3	
22	7.83 and 8.18 (2 x s, 1H)	159.4		H to C20

* Unknown due to signal overlaps

All reagents were purchased from TCI UK Ltd (N^{α}-(5-fluoro-2,4-dinitrophenyl)-L-leucinamide, N^{α}-(5-fluoro-2,4-dinitrophenyl)-D-leucinamide), Alfa Aesar Ltd (L-ornithine hydrochloride, 57% hydriodic acid) and Fluorochem Ltd (D-ornithine hydrochloride). LCMS was performed on a Waters LCMS system, ACQUITY Ultra Performance LC™ (UPLC™) coupled to ACQUITY Photodiode Array (PDA) UV detector and a Xevo TQ-S mass detector.

LCMS Method for Marfey's Analysis

HPLC separation was carried out using a linear gradient of 5 – 70 % MeCN:H_2O (0.1 % formic acid) over 15 minutes and a hold time at 100% of MeCN (0.1 % formic acid) for 1 minute, with an injection volume 1 µL, and a volume flow rate of 0.25 mL/min. Detection was carried out by UV (200-500 nm) and ESI-MS. Column (temp): ACQUITY UPLC® BEH C_{18} 1.7 µm, 2.1 x 150 mm Column (40 °C)

Marfey's Analysis of mirubactin C

To determine the absolute stereochemistry of mirubactin C by Marfey's analysis, 500 µg of mirubactin C was dissolved in 400 µL of 57 % HI and the reaction mixture heated to 110 °C in an oil bath for 3 hours. After this time the reaction mixture was evaporated to dryness under a gentle stream of nitrogen gas overnight.

The residue material was dissolved in 240 µL of 1 M aq. $NaHCO_3$, to which was added 170 µL 1 % N^{α}-(5-fluoro-2,4-dinitrophenyl)-L-leucinamide in acetone and the reaction mixture stirred for 1 hour at 40 °C. After which the reaction was quenched with 60 µL of 2 M aq. HCl.

20 µL of this sample was diluted with 980 µL of HPLC grade water and analysed following the above LCMS method, versus standards.

Preparation of Standards for Marfey's Analysis

Marfey's standards for D-ornithine and L-ornithine were prepared by mixing 50 µL of a 50 mM aq. solution of either D-ornithine or L-ornithine hydrochloride with 20 µL of a 1 M aq. solution of $NaHCO_3$ and 100 µL of 1 % L-FDLA in acetone. The reaction mixtures were heated to 40 °C in a water bath for 1 hour to form the corresponding L-FDLA/D-ornithine and L-FDLA/L-ornithine adducts. 17 µL of each of these samples was diluted with 983 µL of HPLC grade water and analysed following the above LCMS method.

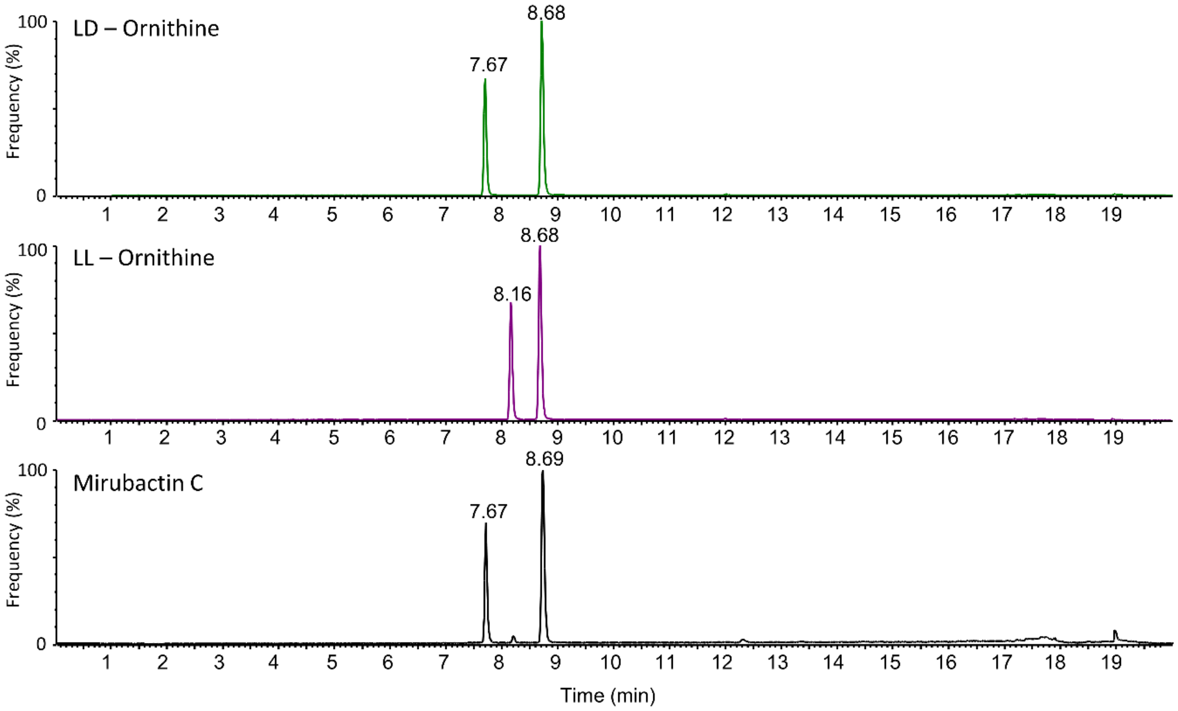

Supplementary Figure 3 LCMS extracted ion chromatogram (EIC m/z = 427.2, +/- 0.25 Da) showing the samples of N^{α}-(5-fluoro-2,4-dinitrophenyl)-L-leucinamide (L-FDLA) derivatized mirubactin C (bottom) in comparison to L-FDLA/D-Ornithine (top) and L-FDLA/L-Ornithine standards (middle). LCMS Extracted ion chromatogram showing the sample of L-FDLA + mirubactin C (bottom) matched with L-FDLA + D-Ornithine standard (top)

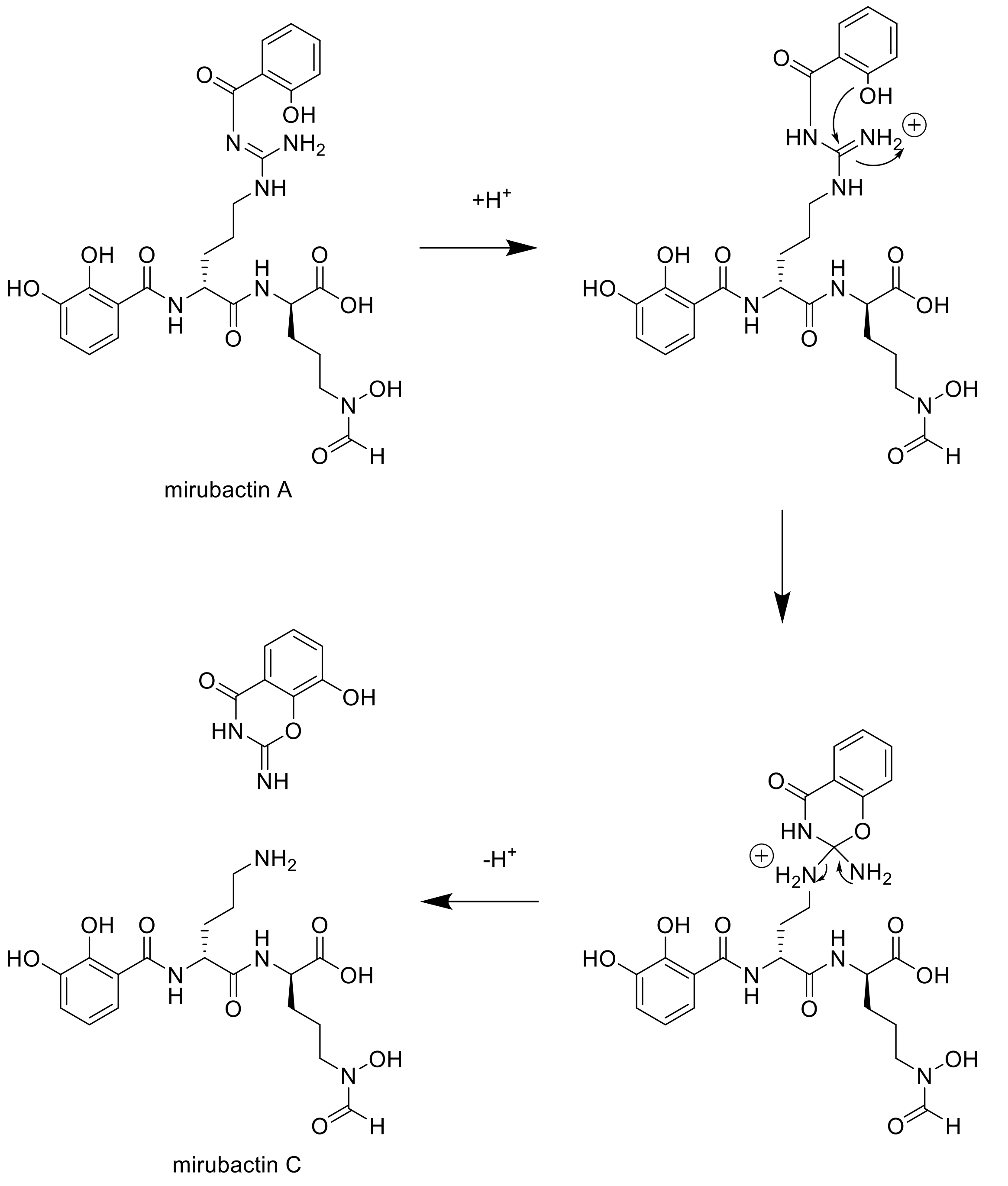

Supplementary Figure 4 Proposed degradation pathway of mirubactin A to mirubactin C based on Kishimoto et al (2014).[34]

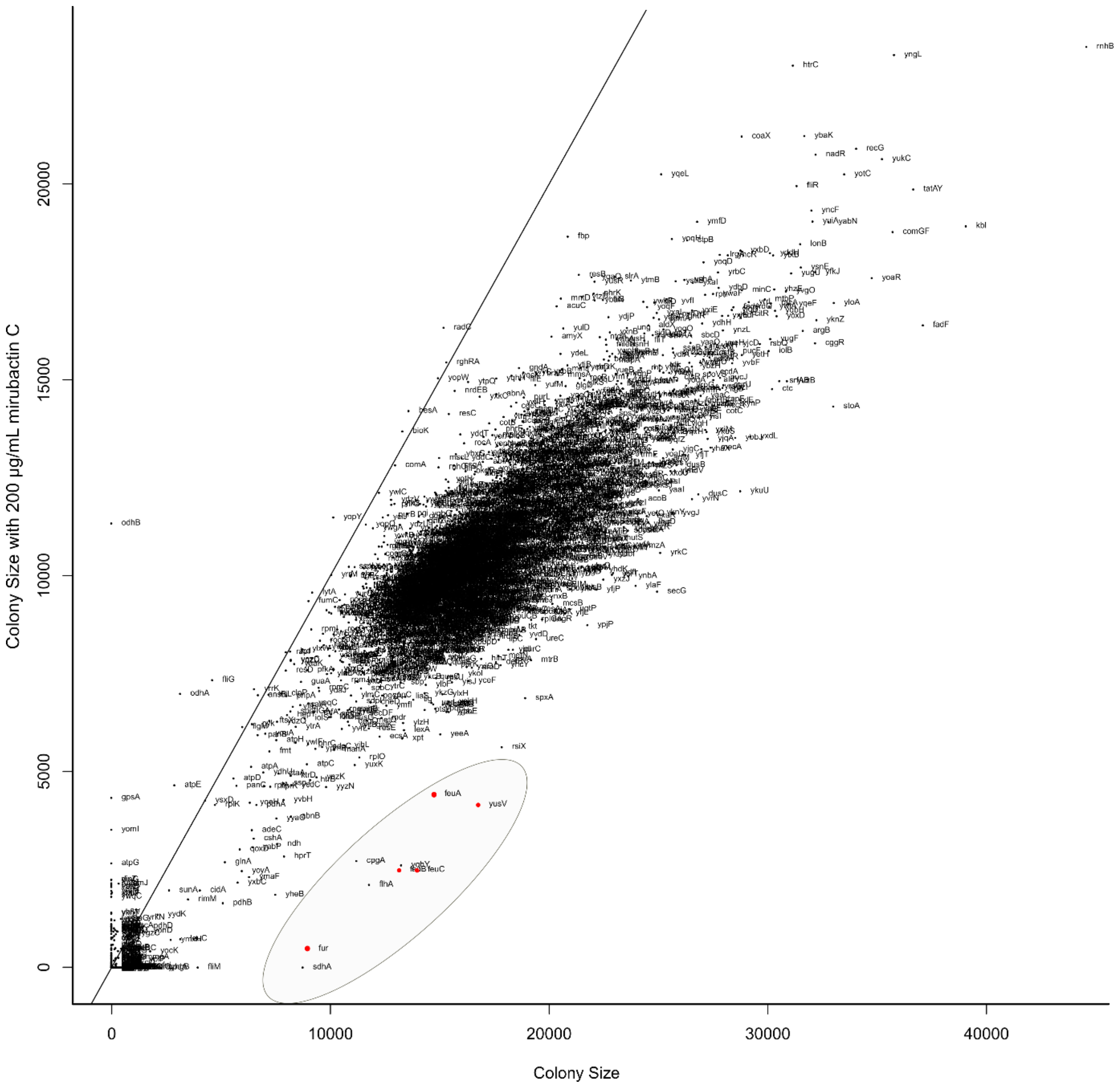

Supplementary Figure 5 Effects of mirubactin C on the *B. subtilis* global mutant collection. Most affected genes involved in iron homeostasis are marked on in red. The cut-off for table 1 was arbitrarily drawn and indicated by an oval.
